# Association of a Positive Drug Screening for Cannabis With Mortality and Hospital Visits Among Veterans Affairs Enrollees Prescribed Opioids

**DOI:** 10.1001/jamanetworkopen.2022.47201

**Published:** 2022-12-16

**Authors:** Salomeh Keyhani, Samuel Leonard, Amy L. Byers, Tauheed Zaman, Erin Krebs, Peter C. Austin, Tristan Moss-Vazquez, Charles Austin, Friedhelm Sandbrink, Dawn M. Bravata

**Affiliations:** 1 Department of Medicine, University of California, San Francisco; 2 San Francisco VA Medical Center, San Francisco, California; 3 Northern California Institute for Research and Education, San Francisco; 4 Department of Psychiatry and Behavioral Sciences, University of California, San Francisco; 5 Addiction Recovery and Treatments Services, San Francisco VA Health Care System, San Francisco, California; 6 Center for Care Delivery and Outcomes Research, Minneapolis VA Health Care System, Minneapolis, Minnesota; 7 Department of Medicine, University of Minnesota Medical School, Minneapolis; 8 Institute of Health Policy, Management and Evaluation, University of Toronto; 9 Richard L. Roudebush VA Medical Center, Indianapolis, Indiana; 10 National Pain Management, Opioid Safety and Prescription Drug Monitoring Program, Veterans Health Administration, Washington, District of Columbia; 11 Department of Neurology, George Washington University, Washington District of Columbia; 12 Departments of Medicine and Neurology, Indiana University School of Medicine, Indianapolis; 13 Regenstreif Institute, Indianapolis Indiana

## Abstract

**Importance:**

Cannabis has been proposed as a therapeutic with potential opioid-sparing properties in chronic pain, and its use could theoretically be associated with decreased amounts of opioids used and decreased risk of mortality among individuals prescribed opioids.

**Objective:**

To examine the risks associated with cannabis use among adults prescribed opioid analgesic medications.

**Design, Setting, and Participants:**

This cohort study was conducted among individuals aged 18 years and older who had urine drug screening in 2014 to 2019 and received any prescription opioid in the prior 90 days or long-term opioid therapy (LTOT), defined as more than 84 days of the prior 90 days, through the Veterans Affairs health system. Data were analyzed from November 2020 through March 2022.

**Exposures:**

Biologically verified cannabis use from a urine drug screen.

**Main Outcomes and Measures:**

The main outcomes were 90-day and 180-day all-cause mortality. A composite outcome of all-cause emergency department (ED) visits, all-cause hospitalization, or all-cause mortality was a secondary outcome. Weights based on the propensity score were used to reduce confounding, and hazard ratios [HRs] were estimated using Cox proportional hazards regression models. Analyses were conducted among the overall sample of patients who received any prescription opioid in the prior 90 days and were repeated among those who received LTOT. Analyses were repeated among adults aged 65 years and older.

**Results:**

Among 297 620 adults treated with opioids, 30 514 individuals used cannabis (mean [SE] age, 57.8 [10.5] years; 28 784 [94.3%] men) and 267 106 adults did not (mean [SE] age, 62.3 [12.3] years; *P* < .001; 247 684 [92.7%] men; *P* < .001). Among all patients, cannabis use was not associated with increased all-cause mortality at 90 days (HR, 1.07; 95% CI, 0.92-1.22) or 180 days (HR, 1.00; 95% CI, 0.90-1.10) but was associated with an increased hazard of the composite outcome at 90 days (HR, 1.05; 95% CI, 1.01-1.07) and 180 days (HR, 1.04; 95% CI, 1.01-1.06). Among 181 096 adults receiving LTOT, cannabis use was not associated with increased risk of all-cause mortality at 90 or 180 days but was associated with an increased hazard of the composite outcome at 90 days (HR, 1.05; 95% CI, 1.02-1.09) and 180 days (HR, 1.05; 95% CI, 1.02-1.09). Among 77 791 adults aged 65 years and older receiving LTOT, cannabis use was associated with increased 90-day mortality (HR, 1.55; 95% CI, 1.17-2.04).

**Conclusions and Relevance:**

This study found that cannabis use among adults receiving opioid analgesic medications was not associated with any change in mortality risk but was associated with a small increased risk of adverse outcomes and that short-term risks were higher among older adults receiving LTOT.

## Key Points

**Question:**

Is cannabis use associated with increased risk of mortality among patients prescribed opioid analgesics?

**Findings:**

In this cohort study among 297 620 patients in the Veterans Affairs health system treated with opioids in the prior 90 days, cannabis use was not associated with increased mortality among participants aged less than 65 years, but was associated with increased 90-day mortality among those aged 65 years and older receiving long-term opioid therapy. No increase in mortality was found at 180 days.

**Meaning:**

This study found that cannabis use was associated with increased short-term mortality risk among adults aged 65 years and older receiving long-term prescription opioids.

## Teaser

This cohort study investigates the association of cannabis use with mortality among veterans receiving opioids.

## Introduction

Receipt of prescription opioids is associated with adverse events and mortality. Cannabis has been proposed as a therapeutic with potential opioid-sparing properties that could be associated with reduced risk of adverse events among patients treated with prescription opioids. However, tetrahydrocannabinol (THC), the active compound in cannabis, is a psychoactive drug with known adverse effects. In randomized clinical trials examining THC-based pharmaceuticals in the management of pain, adverse events included dizziness, sedation, confusion, loss of balance, nausea, vomiting, and hallucination.^1^ Opioids have similar adverse effects but can also cause respiratory depression and death, so the combination of cannabis and opioids could theoretically be associated with greater harm than either drug alone.^2^

Pain is among the most common reasons for use of medical cannabis, and national surveys suggest that cannabis use is common among people prescribed opioids for chronic pain.^3^ Although prior studies^4,5^ have examined self-reported outcomes associated with cannabis use among patients prescribed opioids, no study to our knowledge has examined potential adverse effects (such as emergent care, hospitalization, or mortality) associated with cannabis use in this population. Understanding outcomes associated with cannabis use among patients using opioids is important to patients, clinicians, and policymakers. The US Department of Veterans Affairs (VA) provides a unique opportunity to study the association of cannabis use with health outcomes among individuals prescribed opioids. Urine drug screening among patients prescribed opioids for pain has been used as a risk mitigation strategy. Compliance with urine drug screening in this population has been tracked since 2013 and was mandated in 2014.^6^ More than 90% of patients prescribed long-term opioids for chronic pain received urine drug screening.^7^ Cannabis is measured in the urine drug screening, providing an opportunity to classify patients who received opioids for chronic pain by exposure status (concomitant cannabis use vs no cannabis use).

To better understand health outcomes in this population, we developed a retrospective cohort to examine the association of cannabis use with health outcomes among individuals who received any prescription opioid in the prior 90 days. We repeated this analysis among individuals who received long-term opioid therapy (LTOT; ≥84 of the prior 90 days). Because older adults may experience worse side effects associated with psychoactive medications, we repeated analyses among patients who were aged 65 years or older.

## Methods

The institutional review board of the University of California, San Francisco, approved this study and waived the need for patient consent because the research involved no more than minimal risk to participants. We followed the Strengthening the Reporting of Observational Studies in Epidemiology (STROBE) reporting guideline.

### Study Design, Setting, Participants, and Procedures

Using national VA and Medicare data,^8^ we designed a cohort study of adults who received their first urine drug screening in the VA outpatient setting between January 1, 2014, and December 31, 2019. Adults were eligible if they received any prescribed opioid analgesic in the 90 days prior to the drug screening. We excluded anyone potentially at the end of life (eg, residing in a VA community living center [nursing home], receiving palliative care, or receiving inpatient cancer chemotherapy) or with a Care Assessment Needs (CAN) score greater than 99. The CAN score is a validated measure, updated weekly, that estimates 90-day mortality among patients in VA primary care; the score is the patient’s risk percentile (range, 0 [low risk] to 99 [highest risk]),^9^ so a CAN score of 99 suggests a very high likelihood of death within 90 days. In addition, we excluded individuals prescribed THC-containing medications (eg, dronabinol) and anyone treated in an opiate treatment program (eg, methadone maintenance clinic). We identified 312 164 veterans receiving opioids who met inclusion criteria (30 514 adults who used cannabis and 267 106 adults who did not use cannabis) (Figure). The index date for cohort entry was defined as the date of the first urine drug screening. We followed up veterans for 180 days after their index date. We limited follow-up to 180 days because the exposure was based on a single biologic measurement of cannabis use and we wanted to ensure that outcomes were temporally near the exposure assessment. All data on exposure, outcome, and covariate measurements were retrieved from the VA Corporate Data Warehouse, VA community care files, Centers for Medicare & Medicaid Services (CMS) data, and Mortality Data Repository.^10,11^

**Figure.**
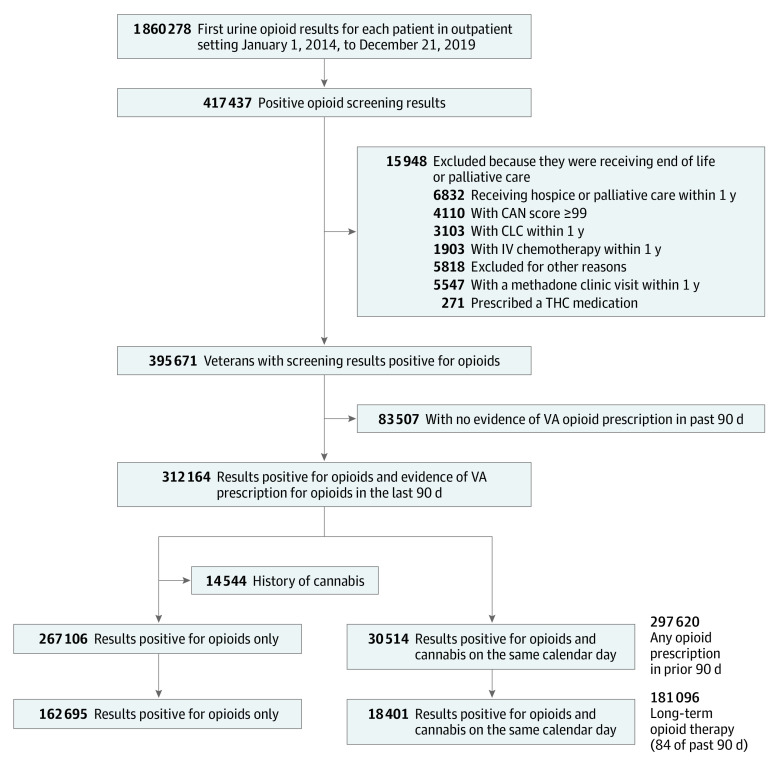
Cohort Construction There were 297 620 patients with any opioid prescription in the prior 90 days and 181 096 patients with long-term opioid therapy (ie, 84 of past 90 days). CAN indicates Care Assessment Needs; CLC, community living center; IV, intravenous; THC, tetrahydrocannabinol; VA, US Department of Veterans Affairs.

### Outcomes

Our primary outcome was death due to any cause within 90 or 180 days of the index urine drug screening. A primary outcome of mortality was chosen because other psychoactive drugs in combination with opioids (eg, benzodiazepines and gabapentinoids) have been associated with increased mortality.^12,13^ The VA Mortality Data Repository and VA Vital Status Files were used to identify mortality.

Our secondary outcome was the composite end point of all-cause hospitalization, all-cause emergency department (ED) visit, or all-cause mortality within 90 or 180 days of the urine drug screening. We chose this secondary composite outcome (time to first event) because findings from several studies^14,15^ suggested that cannabis use was associated with increased rates of ED visits and hospitalization. In addition, the composite outcome captures all 3 possible harms in the period under observation.

### Covariates

Demographic variables included age, sex (men or women as recorded in administrative files), marital status, race and ethnicity (as recorded in VA administrative files), and VA priority score (a measure that includes income and disability).^16^ Recorded race variables in VA data include American Indian or Alaskan Native, Asian, Black, Native Hawaiian or other Pacific Islander, and White. Hispanic ethnicity is also recorded in VA data. We included race and ethnicity data given that the prevalence of cannabis use in the US population varies by race and ethnicity. Medical conditions included hypertension, hyperlipidemia, diabetes, stroke, transient ischemic attack, paralytic syndromes, ischemic heart disease, myocardial infarction, peripheral vascular disease, abdominal aortic aneurysm, heart failure, atrial fibrillation, other cardiac arrythmia, chronic kidney disease, asthma, bronchiectasis, pulmonary embolism, deep venous thrombosis, cirrhosis, hepatitis, Parkinson disease, other extrapyramidal disease, multiple sclerosis, seizure disorder, fall, orthostatic hypotension, rheumatoid arthritis or other inflammatory condition, cancer, HIV/AIDS, lung disease due to external cause, bronchitis, upper respiratory infection, necrotic lung, respiratory failure, chronic obstructive lung disease, pulmonary fibrosis, pneumonia, sleep apnea, dementia, mental health (ie, depression, bipolar disorder, psychosis, and anxiety disorder), and pain-related diagnosis (ie, back and spine disorder, neck and spine disorder, osteoarthritis, neuropathy, headache, and traumatic brain injury). We used at least 1 inpatient *International Classification of Diseases, Ninth Revision *(*ICD-9*) or *International Statistical Classification of Diseases and Related Health Problems, Tenth Revision* (*ICD-10*) code or 2 outpatient diagnosis codes in the 2 years prior to the index urine drug screening to identify the presence of comorbidities. We constructed a Charlson comorbidity index based on relevant medical conditions.^17^

We also collected data on procedures in the past 2 years, including dialysis, coronary intervention, defibrillator use, coronary artery bypass graft, receipt of tracheostomy, receipt of oxygen, and history of lung resection. We classified veterans who were enrolled in home-based primary care, used adult day care services, or received a long-term care assessment as functionally impaired.

We used a combination of *ICD-9* and *ICD-10* codes on housing instability and receipt of VA housing services to identify veterans with evidence of housing insecurity. Current tobacco use was identified using a published, existing VA algorithm.^18^ We extracted data on substance use (alcohol or drug) using *ICD-9* and *ICD-10* codes. The presence of a single drug or alcohol use disorder or dependence code in the prior 2 years was considered indicative of the presence of a substance use condition. We also extracted the Alcohol Use Disorders Identification Test (AUDIT-C) score for each veteran and defined an AUDIT-C score as elevated if it was greater than 4 for women and greater than 5 for men. The AUDIT-C is scored on a scale of 0 to 12; scores of 0 reflect no alcohol use. Data on the presence of drugs (eg, cocaine or benzodiazepine) in the index urine drug screening were also extracted.

Body mass index (calculated as weight in kilograms divided by height in meters squared), blood pressure levels, kidney function, hemoglobin levels, and albumin levels were extracted from the Corporate Data Warehouse.^19^ Data on ED visits, hospitalizations, and intensive care unit admissions in the past year were also collected. We extracted the CAN score for 90-day mortality risk.^20^ Additionally, we extracted data on psychoactive drug dispensing (ie, opioid analgesic, long-acting opioid analgesic, opioid dose in morphine-milligram equivalents per past 90 days, muscle relaxant, antidepressant, antipsychotic, sedative, and gabapentinoid) and constructed a count for each patient on the number of psychoactive medications dispensed in the prior 90 days.

Over the study period, opioid prescribing practices changed in the VA.^7^ To account for these secular differences, we included the year the urine drug screening was administered as a covariate in analyses. To account for potential geographic differences in clinician prescribing practices and environmental differences associated with the legalization status of the state in which each adult resided, we included a variable representing the facility where the urine drug screening was administered in analyses. There were 130 facilities included in the analysis.

### Statistical Analysis

Baseline covariates were compared between adults who used cannabis and those who did not. Standardized differences were used to quantify differences in means and prevalence of binary covariates between exposure groups. Standardized differences (for comparing means), variance ratios (for comparing variances), and plots of empirical cumulative distribution functions were used to quantify differences in the distribution of continuous covariates between exposure groups.

Separately among LTOT and any-opioid groups, we used logistic regression to estimate the propensity score for cannabis use. Variables listed in eTable 1 in Supplement 1 were included in propensity models. Weights that allowed us to estimate the average treatment effect in the treated (ATT) were computed as *Z* + (1 − *Z*) × PS/(1 − PS), where *Z* denotes treatment status (*Z* = 1 for cannabis use and *Z* = 0 for nonuse) and PS denotes estimated propensity score. Weighted (adjusted) hazard ratios (HRs) were estimated using Cox regression models that incorporated PS weights. Robust variance estimators were used to account for within-person homogeneity induced by weighting.^20^ We examined the association of cannabis use with 90-day and 180-day mortality and the combined outcome at 90 days and 180 days. We repeated analyses in the LTOT and any-opioid groups after restriction to older veterans (ie, those aged ≥65 years). The propensity-weighting approach was prespecified prior to analyses. As a sensitivity analysis of results, we repeated analyses described previously using a propensity-matching approach, with 1:1 nearest-neighbor matching without replacement. Findings were considered statistically significant if the CI did not cross the null value or if the 2-sided *P* value was less than .05.

Missing data were minimized by including multiple sources of data to construct the cohort. Race was missing in approximately 5% of the cohort (16 679 of 297 620 individuals [5.6%]) and was included as a separate category in the propensity model. Statistical analyses were performed with RStudio version 2022.02.0 + 443 (RStudio), and packages included dplyr, cobalt, matchit, splines, tableone, and survey. Data were analyzed from November 2020 through March 2022.

To examine baseline characteristics that were not well captured in structured data and better understand how individuals receiving LTOT who also used cannabis may have been different from individuals who did not use cannabis, we conducted electronic health record (EHR) review of a random stratified sample of cohort-eligible patients with and without cannabis use. We stratified the sample by the presence of cannabis in the urine drug screening and randomly sampled 1219 patients who were receiving prescription LTOT in the VA. The sample included veterans from all 18 Veterans Integrated Services Networks in the VA. We collected data on 39 characteristics that encompassed health behaviors, adverse outcomes, and clinician actions associated with opioid use from the EHR in the year prior to in the index urine drug screening. Characteristics were first defined by the investigative team and then expanded upon through EHR review as previously unidentified behaviors, outcomes, or clinician actions were encountered in EHRs. We categorized information collected on the 39 characteristics into 6 domains: substance use–associated findings and behaviors, alcohol-associated behaviors, treatment-associated behaviors, adverse outcomes associated with opioid use, clinician actions associated with opioid use, and other potential high-risk behaviors. We trained 3 research assistants in medical record review prior to conducting data abstraction. They met weekly with investigators (S.K., A.B., T.Z., and D.M.B.) to review disagreements and resolve questions. Records were reviewed in duplicate until agreement of greater than 90% was achieved across domains. Then, 10% of subsequent records were reviewed in duplicate. Agreement across duplicates was greater than 90.0% across 6 domains, with a mean level of agreement of 96.2%.

## Results

### Baseline Characteristics of Total Sample

Among 297 620 adults who received any opioid prescription, 30 514 adults used cannabis (mean [SE] age, 57.8 [10.5] years; 28 784 [94.3%] men; 4686 Black [15.4%]; 23 076 White [75.6%]; and 1321 Hispanic [4.3%]) and 267 106 adults did not use cannabis (mean [SE] age, 62.3 [12.3] years; *P* < .001; 247 684 [92.7%] men; *P* < .001; 33 719 Black [12.6%] and 212 316 White [79.5%]; and 9901 Hispanic [3.7%]) (Table 1). Medical conditions, including hypertension (17 351 patients [56.9%] vs 184 938 patients [69.2%]; *P* < .001), diabetes (6973 patients [22.9%] vs 93 990 patients [35.2%]; *P* < .001), ischemic heart disease (4546 patients [14.9%] vs 65 171 patients [24.4%]; *P* < .001), heart failure (1573 patients [5.2%] vs 26 014 patients [9.7%]; *P* < .001), chronic kidney disease (2405 patients [7.9%] vs 38 420 patients [14.4%]; *P* < .001), and chronic obstructive lung disease (5775 patients [18.9%] vs 58 882 patients [22.0%]; *P* < .001) were less common among individuals who used cannabis compared with those who did not use cannabis. Adults who used cannabis more commonly had a diagnosis of psychosis (1067 patients [3.5%] vs 7298 patients [2.7%]; *P* < .001), depression (11 659 patients [38.2%] vs 95 580 patients [35.8%]; *P* < .001), posttraumatic stress disorder (7312 patients [24.0%] vs 54 253 patients [20.3%]; *P* < .001), anxiety (6252 patients [20.5%] vs 52 693 patients [19.7%]; *P* = .002), and alcohol use disorder (3467 patients [11.4%] vs 17 246 patients [6.5%]; *P* < .001) at baseline. Adverse health behaviors, including tobacco use (16 863 patients [55.3%] vs 106 201 patients [39.8%]; *P* < .001), were more common among individuals who used cannabis. Adults who used cannabis also received a higher dose of prescription opioids compared with individuals who did not use cannabis (mean [SE] morphine-equivalents per 90 days, 194.2 [5.14] g vs 188.3 [5.68] g; *P* < .001). The expanded version of Table 1 with all 107 variables is in eTable 1 in Supplement 1.

**Table 1.** Baseline Patient Characteristics

Characteristic^a^	Patients, No. (%) (N = 297 620)		*P* value
	No cannabis use (n = 267 106)	Cannabis use (n = 30 514)	
Age, mean (SE)	62.3 (12.3)	57.8 (10.5)	<.001
Sex			
Men	247 684 (92.7)	28 784 (94.3)	<.001
Women	19 422 (7.3)	1730 (5.7)	<.001
Married	144 741 (54.2)	11 948 (39.2)	<.001
Race			
American Indian or Alaska Native	3021 (1.1)	481 (1.6)	<.001
Asian	930 (0.3)	110 (0.4)	.77
Black or African American	33 719 (12.6)	4686 (15.4)	<.001
Native Hawaiian or other Pacific Islander	2328 (0.9)	274 (0.9)	.66
White	212 316 (79.5)	23 076 (75.6)	<.001
Missing	14792 (5.5)	1887 (6.2)	NA
Hispanic	9901 (3.7)	1321 (4.3)	<.001
Comorbid condition			
Hypertension	184 938 (69.2)	17 351 (56.9)	<.001
Hyperlipidemia	163 767 (61.3)	14 297 (46.9)	<.001
Diabetes	93 990 (35.2)	6973 (22.9)	<.001
Stroke	6949 (2.6)	586 (1.9)	<.001
Ischemic heart disease	65 171 (24.4)	4546 (14.9)	<.001
Heart failure	26 014 (9.7)	1573 (5.2)	<.001
Atrial fibrillation	24 898 (9.3)	1570 (5.1)	<.001
Asthma	16 978 (6.4)	1573 (5.2)	<.001
Pulmonary embolism	4275 (1.6)	315 (1.0)	<.001
Deep venous thrombosis	6330 (2.4)	515 (1.7)	<.001
Cirrhosis	8488 (3.2)	1175 (3.9)	<.001
Parkinson disease	2554 (1.0)	121 (0.4)	<.001
Multiple sclerosis	1195 (0.4)	161 (0.5)	.05
Seizure disorder	4472 (1.7)	611 (2.0)	<.001
Rheumatoid arthritis	6989 (2.6)	590 (1.9)	<.001
Chronic kidney disease	38 420 (14.4)	2405 (7.9)	<.001
Dialysis	847 (0.3)	61 (0.2)	.001
AIDS	1144 (0.4)	287 (0.9)	<.001
Lung disease due to external agents	7560 (2.8)	1199 (3.9)	<.001
Bronchitis	4994 (1.9)	403 (1.3)	<.001
Tracheostomy	1069 (0.4)	80 (0.3)	<.001
History of mechanical ventilation	18 967 (7.1)	1364 (4.5)	<.001
Respiratory failure	17 525 (6.6)	1326 (4.3)	<.001
Home oxygen within 1 y	8153 (3.1)	485 (1.6)	<.001
Chronic obstructive lung disease	58 882 (22.0)	5775 (18.9)	<.001
Pneumonia	16 968 (6.4)	1290 (4.2)	<.001
Sleep apnea	38 746 (14.5)	2618 (8.6)	<.001
Dementia	19 662 (7.4)	1383 (4.5)	<.001
Head and neck cancer	2151 (0.8)	310 (1.0)	<.001
Respiratory tract cancer	5696 (2.1)	627 (2.1)	.38
Prostate cancer	12 741 (4.8)	973 (3.2)	<.001
Charlson comorbidity index score, mean (SE)	3.33 (2.34)	2.50 (1.91)	<.001
Psychosis	7298 (2.7)	1067 (3.5)	<.001
Depression	95 580 (35.8)	11 659 (38.2)	<.001
Bipolar	9724 (3.6)	1701 (5.6)	<.001
Posttraumatic stress disorder	54 253 (20.3)	7312 (24.0)	<.001
Anxiety	52 693 (19.7)	6252 (20.5)	.002
Self-harm	585 (0.2)	131 (0.4)	<.001
Substance use			
Opioid use disorder	7881 (3.0)	1291 (4.2)	<.001
Benzodiazepine use	984 (0.4)	178 (0.6)	<.001
Amphetamine use	1852 (0.7)	488 (1.6)	<.001
Other drug use	5096 (1.9)	1412 (4.6)	<.001
Alcohol use disorder	17 246 (6.5)	3467 (11.4)	<.001
Current tobacco use	10 6201 (39.8)	16 863 (55.3)	<.001
Homeless or marginally housed	10 594 (4.0)	2330 (7.6)	<.001
Pain diagnosis			
Back and spine disorder	17 5145 (65.6)	18 826 (61.7)	<.001
Neck and spine disorder	59 197 (22.2)	6747 (22.1)	.84
Osteoarthritis	95 523 (35.8)	8303 (27.2)	<.001
Neuropathy	53 123 (19.9)	4116 (13.5)	<.001
Headache	41 002 (15.4)	5563 (18.2)	<.001
Traumatic brain injury	453 (0.2)	53 (0.2)	.93
Psychoactive drug			
Sum of morphine milligram equivalents per day for prescriptions within 90 d, mean (SE)	188.3 (5.68)	194.2 (5.14)	<.001
Long-acting opioid	59 696 (22.3)	6476 (21.2)	<.001
Benzodiazepine	56 294 (21.1)	6604 (21.6)	.02
GABA drug	72 909 (27.3)	7188 (23.6)	<.001
Muscle relaxant	55 865 (20.9)	6668 (21.9)	<.001
Antidepressant	87 747 (32.9)	9913 (32.5)	.20
Antipsychotic	18 379 (6.9)	2691 (8.8)	<.001

Abbreviation: GABA, γ-aminobutyric acid.

^a^ The full table, including all 107 baseline variables, is available in eTable 1 in Supplement 1.

### Baseline Characteristics of LTOT Group

Among 181 096 adults receiving LTOT, 18 401 adults who used cannabis were younger than 162 695 adults who did not use cannabis (mean [SE] age, 58.4 [9.5] years vs 61.9 [11.6] years; *P* < .001) (eTable 2 in Supplement 1) and had fewer medical conditions. Differences in mental health conditions were small, but these conditions were more common among adults who used cannabis. Adverse health behaviors, including tobacco use (10 485 individuals [57.0%] vs 70 764 individuals [43.5%]; *P* < .001), were more common among adults who used cannabis. Adults who used cannabis also received more prescription opioids compared with adults who did not use cannabis (mean [SE] morphine equivalents per 90 days, 268.6 [542.7] g vs 277.6 [561.3] g; *P* = .03). Opioid use disorder was more common among adults who used cannabis than adults who did not use cannabis (872 individuals [4.7%] vs 5773 individuals [3.5%]; *P* < .001).

### Differences in Propensity-Weighted and Matched Groups

Standardized differences for variables were small in weighted and matched samples, with differences between groups not clinically meaningful, suggesting successful weighting and matching for the overall sample (Table 2, eTable 3 in Supplement 1). Standardized differences in weighted samples and matched samples were 0.022 or less across variables (the maximum difference was found, for example, for propensity-matched mean [SE] age in nonuse vs cannabis use groups: 57.62 [12.37] years vs 57.87 [10.56] years; difference, 0.022). The complete list of 107 variables included in the propensity model is presented in eTable 3 in Supplement 1.

**Table 2.** Propensity-Weighted and Matched Adults With Prescription Opioid Therapy in Past 90 Days^a^

Characteristic	Patients, No. (%)				
	Propensity weighted^b^			Propensity matched^c^	
	No cannabis use (n = 30 923)	Cannabis use (n = 30 514)	No cannabis use (n = 30 514)		Cannabis use (n = 30 514)
Age, mean (SE), y	57.84 (10.51)	57.87 (10.56)	57.62 (12.37)		57.87 (10.56)
Sex					
Men	29 179 (94.36)	28 784 (94.33)	28 702 (94.06)		28 784 (94.33)
Women	1744 (5.64)	1730 (5.67)	1812 (5.94)		1730 (5.67)
Married	11 896 (38.47)	11 948 (39.16)	11 927 (39.09)		11 948 (39.16)
Race					
American Indian or Alaska Native	488 (1.58)	481 (1.58)	479 (1.57)		481 (1.58)
Asian	112 (0.36)	110 (0.36)	108 (0.35)		110 (0.36)
Black or African American	4891 (15.82)	4686 (15.36)	4682 (15.34)		4686 (15.36)
Native Hawaiian or Other Pacific Islander	279 (0.9)	274 (0.9)	276 (0.9)		274 (0.9)
White	23 239 (75.15)	23 076 (75.62)	23 046 (75.53)		23 076 (75.62)
Hispanic	1358 (4.39)	1321 (4.33)	1321 (4.33)		1321 (4.33)
Comorbid condition					
Hypertension	17 581 (56.86)	17 351 (56.86)	17 341 (56.83)		17 351 (56.86)
Hyperlipidemia	14 337 (46.36)	14 297 (46.85)	14 337 (46.98)		14 297 (46.85)
Diabetes	7012 (22.68)	6973 (22.85)	6887 (22.57)		6973 (22.85)
Stroke	602 (1.95)	586 (1.92)	596 (1.95)		586 (1.92)
Ischemic heart disease	4553 (14.72)	4546 (14.9)	4440 (14.55)		4546 (14.9)
Heart failure	1584 (5.12)	1573 (5.16)	1530 (5.01)		1573 (5.16)
Atrial fibrillation	1577 (5.1)	1570 (5.15)	1522 (4.99)		1570 (5.15)
Asthma	1583 (5.12)	1573 (5.16)	1527 (5)		1573 (5.16)
Pulmonary embolism	318 (1.03)	315 (1.03)	297 (0.97)		315 (1.03)
Deep venous thrombosis	531 (1.72)	515 (1.69)	543 (1.78)		515 (1.69)
Cirrhosis	1222 (3.95)	1175 (3.85)	1116 (3.66)		1175 (3.85)
Parkinson disease	121 (0.39)	121 (0.4)	106 (0.35)		121 (0.4)
Multiple sclerosis	159 (0.51)	161 (0.53)	187 (0.61)		161 (0.53)
Seizure disorder	636 (2.06)	611 (2)	622 (2.04)		611 (2)
Rheumatoid arthritis	600 (1.94)	590 (1.93)	575 (1.88)		590 (1.93)
Chronic kidney disease	2419 (7.82)	2405 (7.88)	2407 (7.89)		2405 (7.88)
Dialysis	61 (0.2)	61 (0.2)	70 (0.23)		61 (0.2)
AIDS	316 (1.02)	287 (0.94)	300 (0.98)		287 (0.94)
Lung disease due to an external agent	1262 (4.08)	1199 (3.93)	1149 (3.77)		1199 (3.93)
Bronchitis	404 (1.31)	403 (1.32)	369 (1.21)		403 (1.32)
Tracheostomy	83 (0.27)	80 (0.26)	88 (0.29)		80 (0.26)
Mechanical ventilation in past year	1380 (4.46)	1364 (4.47)	1391 (4.56)		1364 (4.47)
Respiratory failure	1367 (4.42)	1326 (4.35)	1316 (4.31)		1326 (4.35)
Home oxygen within 1 y	488 (1.58)	485 (1.59)	476 (1.56)		485 (1.59)
Chronic obstructive lung disease	5894 (19.06)	5775 (18.93)	5663 (18.56)		5775 (18.93)
Pneumonia	1325 (4.29)	1290 (4.23)	1219 (3.99)		1290 (4.23)
Sleep apnea	2629 (8.5)	2618 (8.58)	2576 (8.44)		2618 (8.58)
Dementia	1391 (4.5)	1383 (4.53)	1341 (4.39)		1383 (4.53)
Cancer					
Head and neck	318 (1.03)	310 (1.02)	284 (0.93)		310 (1.02)
Lung respiratory tract	637 (2.06)	627 (2.05)	620 (2.03)		627 (2.05)
Prostate	994 (3.21)	973 (3.19)	975 (3.2)		973 (3.19)
Mental health					
Psychosis	1099 (3.55)	1067 (3.5)	1032 (3.38)		1067 (3.5)
Depression	11 781 (38.1)	11 659 (38.21)	11 543 (37.83)		11 659 (38.21)
Bipolar disorder	1754 (5.67)	1701 (5.57)	1727 (5.66)		1701 (5.57)
PTSD	7370 (23.83)	7312 (23.96)	7238 (23.72)		7312 (23.96)
Anxiety	6314 (20.42)	6252 (20.49)	6177 (20.24)		6252 (20.49)
Self-harm	139 (0.45)	131 (0.43)	127 (0.42)		131 (0.43)
Substance use					
Disorder					
Opioid	1347 (4.36)	1291 (4.23)	1271 (4.17)		1291 (4.23)
Benzodiazepine	184 (0.6)	178 (0.58)	176 (0.58)		178 (0.58)
Amphetamine	534 (1.73)	488 (1.6)	448 (1.47)		488 (1.6)
Other drug	1552 (5.02)	1412 (4.63)	1333 (4.37)		1412 (4.63)
Alcohol	3664 (11.85)	3467 (11.36)	3387 (11.1)		3467 (11.36)
Current tobacco use	17 294 (55.92)	16 863 (55.26)	16 911 (55.42)		16 863 (55.26)
Alcohol abuse according to AUDIT-C score	2188 (7.07)	2073 (6.79)	2039 (6.68)		2073 (6.79)
Homelessness	2495 (8.07)	2330 (7.64)	2315 (7.59)		2330 (7.64)
Pain diagnosis					
Back and spine disorder	18 943 (61.26)	18 826 (61.7)	18 762 (61.49)		18 826 (61.7)
Neck and spine disorder	6779 (21.92)	6747 (22.11)	6700 (21.96)		6747 (22.11)
Osteoarthritis	8333 (26.95)	8303 (27.21)	8172 (26.78)		8303 (27.21)
Neuropathy	4112 (13.3)	4116 (13.49)	4042 (13.25)		4116 (13.49)
Headache	5727 (18.52)	5563 (18.23)	5576 (18.27)		5563 (18.23)
Traumatic brain injury	55 (0.18)	53 (0.17)	55 (0.18)		53 (0.17)
Psychoactive medication use					
Sum of morphine milligram equivalents/d for prescriptions within 90 d	191.96 (451.68)	194.22 (456.72)	191.98 (469.17)		194.22 (456.72)
Long-acting opioid	6424 (20.77)	6476 (21.22)	6354 (20.82)		6476 (21.22)
Benzodiazepine	6675 (21.59)	6604 (21.64)	6583 (21.57)		6604 (21.64)
GABA drug	7202 (23.29)	7188 (23.56)	7134 (23.38)		7188 (23.56)
Muscle relaxant	6735 (21.78)	6668 (21.85)	6687 (21.91)		6668 (21.85)
Antidepressant	9934 (32.13)	9913 (32.49)	9906 (32.46)		9913 (32.49)
Antipsychotic	2753 (8.9)	2691 (8.82)	2705 (8.86)		2691 (8.82)
Charlson comorbidity index score, mean (SE)	2.51 (1.91)	2.5 (1.91)	2.47 (2.04)		2.5 (1.91)

Abbreviations: AUDIT-C, Alcohol Use Disorders Identification Test; GABA, γ-aminobutyric acid; PTSD, posttraumatic stress disorder.

^a^ The full table, including all 107 baseline variables, is available in eTable 3 in Supplement 1.

^b^ Standardized differences in weighted samples were 0.007 or less for all variables (eTable 3 in Supplement 1).

^c^ Standardized differences for matched samples were 0.022 or less for all variables (eTable 3 in Supplement 1).

Successful weighting and matching among adults receiving LTOT are presented in eTable 4 in Supplement 1. Standardized differences for variables were below accepted thresholds and were 0.018 or less (the maximum difference was found, for example, for propensity-matched low BMI [ie, <25.0] in nonuse vs cannabis use groups: 5124 patients [27.9%] vs 5274 [28.7%]; difference, 0.018).

### Cannabis Use and 90- and 180-Day Outcomes With Propensity Weighting

#### Adults Receiving Any Opioid Prescription in Past 90 Days

Among adults receiving any prescription opioid therapy, cannabis use was not associated with increased mortality at 90 days (HR,1.07; 95% CI, 0.92-1.22) or 180 days (HR, 1.00; 95% CI, 0.90-1.10) (Table 3, eFigure 1 in Supplement 1). However, it was associated with an increased hazard of the composite outcome at 90 days in propensity-weighted groups (HR, 1.05; 95% CI, 1.01-1.07) and 180 days (HR, 1.04; 95% CI, 1.01, 1.06) (Table 3, eFigure 2 in Supplement 1).

**Table 3.** Association of Cannabis Use With Adverse Events in Propensity-Weighting Approach

Outcome	Event No. (%)^b^	90-d All-cause mortality		Events No. (%)^b^	180-d All-cause mortality		Events, No. (%)^b^	90-d Composite outcome^a^		Events, No. (%)^b^	180-d Composite outcome^a^	
		HR (95% CI)	*P* value		HR (95% CI)	*P* value		HR (95% CI)	*P* value		HR (95% CI)	*P* value
Any prescription opioid in past 90 d												
Total, No.	2703	NA	NA	5581	NA	NA	63 023	NA	NA	97 597	NA	NA
Opioid only	2404 (0.9)	1 [Reference]	.36	5021 (1.88)	1 [Reference]	.94	56 356 (21.62)	1 [Reference]	.001	87 619 (33.61)	1 [Reference]	.002
Opioid and cannabis	299 (0.98)	1.07 (0.92-1.22)		560 (1.84)	1.00 (0.90-1.10)		6667 (22.59)	1.05 (1.01-1.07)		9978 (33.81)	1.04 (1.01-1.06)	
LTOT^c^												
Total, No.	1347	NA	NA	2997			37 406	NA	NA	59 021	NA	NA
Opioid only	1197 (0.74)	1 [Reference]	.09	2707 (1.66)	1 [Reference]	.78	33 622 (20.87)	1 [Reference]	.003	53 150 (32.99)	1 [Reference]	.002
Opioid and cannabis	150 (0.82)	1.18 (0.97-1.43)		290 (1.58)	1.02 (0.88-1.17)		3784 (20.85)	1.05 (1.02-1.09)		5871 (32.34)	1.05 (1.02-1.09)	

Abbreviations: HR, hazard ratio; LTOT, long-term opioid therapy; NA, not applicable.

^a^ Composite outcome is 90-day emergency department visits, hospitalization, or all-cause mortality.

^b^ No. of events is unadjusted.

^c^ Opioids on more than 84 of the past 90 days.

#### Adults Receiving LTOT in Past 90 Days

Among adults receiving LTOT, cannabis use was not associated with increased risk of mortality at 90 days (HR, 1.18; 95% CI, 0.97-1.43) or 180 days (HR, 1.02; 95% CI, 0.88-1.17). However, it was associated with an increased hazard of the composite outcome at 90 days (HR, 1.05; 95% CI, 1.02-1.09) and 180 days (HR, 1.05; 95% CI, 1.02-1.09) in propensity-weighted samples (Table 3).

### Cannabis Use and 90- and 180-Day Outcomes With Propensity-Matching

Results were consistent using a propensity-matching approach for adults receiving any prescription opioid therapy and adults receiving LTOT (eTable 5 in Supplement 1). In 1 analysis, estimated HRs were of similar magnitude and in the same direction in both approaches but were statistically significant in the matching approach but not the weighting approach. In the propensity-matching approach, cannabis use was associated with an increased hazard of mortality at 180 days (HR, 1.13; 95% CI, 1.01-1.27) among adults receiving any prescription opioid therapy.

### Analyses Among Adults Aged 65 Years and Older

Baseline characteristics of 135 413 adults aged 65 years and older receiving any prescription opioid therapy in the past 90 days are displayed in eTable 6 in Supplement 1. Baseline characteristics of 77 791 adults aged 65 years and older receiving LTOT in the past 90 days are displayed in eTable 7 in Supplement 1. The distributions of medical and mental health comorbidities and health behaviors were similar to those in the overall sample. Successful weighting and matching, with standardize differences of 0.035 or less across variables for adults aged 65 years and older receiving any prescription opioid and adults aged 65 years and older receiving LTOT are presented in eTable 8 and eTable 9 in Supplement 1.

#### Outcomes at 90 and 180 Days With Propensity Weighting

Among adults aged 65 years and older receiving any prescription opioids in the past 90 days, cannabis use was not associated with increased mortality at 90 days or 180 days in propensity-weighted samples. Similarly, it was not associated with an increased hazard of the composite outcome at 90 days or 180 days in propensity-weighted samples (eTable 10 in Supplement 1).

In analyses restricted to adults aged 65 years and older receiving LTOT, cannabis use was associated with mortality at 90 days (HR, 1.55; 95% CI, 1.17-2.04) but not 180 days (HR, 1.17; 95% CI, 0.95-1.44) in propensity-weighted samples. Among adults aged 65 years and older receiving LTOT, cannabis use was not associated with the composite outcome at 90 days (HR, 1.05; 95% CI, 0.98-1.13) or 180 days (HR, 1.03; 95% CI, 0.97-1.08) (eTable 10 in Supplement 1).

#### Outcomes at 90 and 180 Days With Propensity Matching

In the propensity-matching approach, cannabis use was again associated with increased short-term risks among adults aged 65 years and older receiving LTOT who used cannabis. In this population, cannabis use was associated with increased mortality at 90 days (HR, 1.57, 95% CI, 1.07-2.29). Also consistent with the propensity-weighting approach, there was no association between cannabis use and mortality at 180 days (HR, 1.18; 95% CI, 0.90-1.50) (eTable 10 in Supplement 1).

Remaining results using a propensity-matching approach were consistent with those of the propensity-weighting approach. In 3 analyses, estimated HRs were of similar magnitude and in the same direction in both approaches but were statistically significant in the matching approach but not the weighting approach. In the propensity-matching approach, cannabis use was associated with an increased hazard of mortality at 90 days (HR, 1.41; 95% CI, 1.09-1.82) among adults aged 65 years and older receiving opioid therapy in the prior 90 days. Among adults aged 65 years and older receiving LTOT, cannabis use was also associated with increased hazard of the composite outcome at 90 days (HR, 1.09; 95% CI, 1.01-1.19) and 180 days (HR, 1.08; 95% CI, 1.01-1.16).

### Characteristics Associated With Prescription Opioid Use Extracted From EHR Review

Among 1219 veterans randomly selected for EHR review (eTable 11 in Supplement 1), 556 individuals used cannabis and 663 individuals did not use cannabis. Substance use–related behaviors (123 individuals [22.1%] vs 64 individuals [9.7%], *P* < .001), alcohol use–related behaviors (334 individuals [60.1%] vs 335 individuals [50.5%]; *P* = .001), adverse treatment behaviors (254 individuals [45.7%] vs 256 individuals [38.6%]; *P* = .01), adverse outcomes related to opioid use (24 individuals [7.7%] vs 9 individuals [1.4%]; *P* = .003), and clinician actions related to opioid use (258 individuals [46.4%] vs 96 individuals [14.5%]; *P* < .001) were more common among adults who used cannabis than among adults who did not use cannabis. For example, in the substance use–related domain, adults who used cannabis were more likely to screen positive for other substance use (eg, cocaine) (46 individuals [8.3%] vs 32 individuals [4.8%]), request specific opioid dosages (96 individuals [17.3%] vs 83 individuals [12.5%]), run out of opioids or request early refills (94 individuals [16.9%] vs 84 individuals [12.7%]), have unauthorized dose escalation (85 individuals [15.3%] vs 73 individuals [11.0%]), and have evidence of self-inflicted harm or threats of self-inflicted harm associated to opioids (19 individuals [3.4%] vs 5 individuals [0.8%]) compared with adults who did not use cannabis (Table 4). In an analysis among adults aged 65 years and older, we found similar results (eTable 12 in Supplement 1).

**Table 4.** Behaviors, Adverse Outcomes, and Clinician Actions Associated With Opioid Use From Record Review

Factor	Patients, No. (%) (N = 1219)			*P* value
	No cannabis use (n = 663)	Cannabis use (n = 556)	Total (n = 1219)	
Substance use–related findings and behaviors	64 (9.7)	123 (22.1)	187 (15.3)	<.001
Cannabis use disorder diagnosis, abuse, addiction, or dependence	2 (0.3)	61 (11)	63 (5.2)	
Urine drug screening positive for substance other than cannabis	32 (4.8)	46 (8.3)	78 (6.4)	
Active drug use disorder diagnosis, abuse, addiction, or dependence	17 (2.6)	24 (4.3)	41 (3.4)	
Using substance not prescribed by clinician to control pain	19 (2.9)	19 (3.4)	38 (3.1)	
Active illicit drug use in past year (other than cannabis)	8 (1.2)	14 (2.5)	22 (1.8)	
DUI, trauma, crash, or arrest associated with intoxication or substance use	0	1 (0.2)	1 (0.1)	
Alcohol use–related behaviors	335 (50.5)	334 (60.1)	669 (54.9)	.001
Alcohol use within abstraction period	335 (50.5)	333 (59.9)	668 (54.8)	
Active alcohol use disorder or dependence	16 (2.4)	19 (3.4)	35 (2.9)	
Adverse treatment behaviors	256 (38.6)	254 (45.7)	510 (41.8)	.01
Requesting specific opioid or dosage	83 (12.5)	96 (17.3)	179 (14.7)	
Running out of opioid or requesting early refill	84 (12.7)	94 (16.9)	178 (14.6)	
Unauthorized or unsanctioned dose escalation	73 (11.0)	85 (15.3)	158 (13)	
ED visit to get opioid	31 (4.7)	35 (6.3)	66 (5.4)	
Resisting therapy changes or alternative therapy	33 (5)	34 (6.1)	67 (5.5)	
Obtaining opioids from non-VA clinician	34 (5.1)	32 (5.8)	66 (5.4)	
Multiple phone calls or visits requesting opioid	31 (4.7)	31 (5.6)	62 (5.1)	
Reporting lost, ruined, or stolen prescription	25 (3.8)	25 (4.5)	50 (4.1)	
Seeking new clinician due to disagreement over opioid prescription	21 (3.2)	20 (3.6)	41 (3.4)	
Refusing request to taper	17 (2.6)	16 (2.9)	33 (2.7)	
Saving or hoarding unused medication	21 (3.2)	16 (2.9)	37 (3)	
Obtaining opioid from street or other nonmedical source	5 (0.8)	15 (2.7)	20 (1.6)	
Resisting referral to other pain specialist	12 (1.8)	12 (2.2)	24 (2)	
Soliciting opioids from multiple clinicians in VA	4 (0.6)	10 (1.8)	14 (1.1)	
Diagnosed with opioid use disorder	4 (0.6)	9 (1.6)	13 (1.1)	
Obtaining opioids from multiple non-VA clinicians	4 (0.6)	7 (1.3)	11 (0.9)	
STORM note from pharmacy indicating moderate or high risk of harm from opioid use	3 (0.5)	2 (0.4)	5 (0.4)	
Adverse outcomes related to opioid use	9 (1.4)	24 (7.73)	40 (27.1)	.003
Evidence of self-inflicted harm, threats, or self-inflicted harm associated with opioids	5 (0.8)	19 (3.4)	24 (2)	
Opioid overdose or use of naloxone	3 (0.5)	3 (0.5)	6 (0.5)	
Injury or intoxication from opioid	3 (0.5)	3 (0.5)	6 (0.5)	
Legal issues associated with opioid	0	1 (0.2)	1 (0.1)	
Clinician actions related to opioid use	96 (14.5)	258 (46.4)	354 (29.0)	<.001
Clinician acted on polydrug use	0	211 (37.9)	211 (17.3)	
Clinician documented plans to terminate or reduce opioids	93 (14)	179 (32.2)	272 (22.3)	
Patient discharged from practice for aberrant behavior or placed on watch list	2 (0.3)	4 (0.7)	6 (0.5)	
Patient referred to disruptive behavior committee	2 (0.3)	4 (0.7)	6 (0.5)	
Patient told to leave or been banned from clinic, hospital, or ED due to behavior associated with an opioid	0	1 (0.2)	1 (0.1)	
Other high-risk behaviors	30 (4.5)	42 (7.6)	72 (5.9)	.03
Patient threat associated with opioids	14 (2.1)	19 (3.4)	33 (2.7)	
>2 Missed primary care appointments	10 (1.5)	15 (2.7)	35 (2.9)	
Patient or family reports problems with opioid addiction	7 (1.1)	9 (1.6)	16 (1.3)	
Requesting non-VA opioid prescription	0 (0)	2 (0.4)	2 (0.2)	
Selling or giving away prescription drugs	1 (0.2)	1 (0.2)	2 (0.2)	

Abbreviations: DUI, driving under the influence; ED, emergency department; STORM, Stratification Tool for Opioid Risk Mitigation; VA, US Department of Veterans Affairs.

## Discussion

In this cohort study, we did not find evidence of reduced harms associated with cannabis use among individuals prescribed opioids. However, we found that cannabis use was associated with increased risk of ED visits, hospitalization, or mortality at 90 days and 180 days among adults prescribed prescription opioids. In individuals aged 65 years or older receiving LTOT, we found evidence of significantly increased short-term risks. These data suggest that cannabis use, like other psychoactive drugs, may be associated with harms if used in combination with prescription opioids.

Several population-based studies examining the association of medical and recreational legalization of cannabis with various metrics of health status have suggested that state-based legalization of cannabis may be associated with decreased opioid prescribing^21,22^ and decreased opioid-associated deaths at the population level.^21,23,24^ Another study^25^ found no association of cannabis legalization with opioid overdose rates. A 2022 study^26^ found that increasing prevalence of retail cannabis dispensaries was associated with increased opioid-associated mortality. While the literature on the public health outcomes associated with legalization is evolving and yet unclear, these studies did not examine the association of cannabis use with health outcomes among individuals prescribed opioids at the patient level. Although we are unaware of other studies that have examined the association of cannabis use with clinical outcomes, such as ED visits, hospitalization, or mortality, among individuals prescribed opioids, several studies have examined the association of cannabis use with other health metrics in this population at increased risk. A cross-sectional study^4^ of 186 patients with chronic back pain found that medical cannabis use was associated with reduced prescription opioid use. However, larger prospective studies in select populations have not confirmed those findings.^27^ A prospective cohort study^5^ of 1514 patients with chronic pain found that cannabis use among patients using opioids was not associated with reduced opioid use or opioid discontinuation. Similarly we did not find an association with a reduction in harms.

In our 2 largest analytic samples (ie, all patients receiving opioid therapy and all patients receiving LTOP), we found that cannabis use was associated with some risks among individuals prescribed opioids. There was a small increased risk of the composite outcome of ED visits, hospitalization, or mortality in both analytic samples. We also found evidence of increased risks among adults aged 65 years and older receiving LTOT. In this high-risk group, cannabis use was associated with a higher risk of mortality at 90 days in both propensity method approaches. Our finding of statistically significant harms at 90 but not 180 days among adults aged 65 years and older receiving LTOT may be associated with changes in opioid prescribing as a result of findings in the urine drug screening. This older population receiving LTOT had increased risk, and clinicians may have responded to dual cannabis and opioid use. While the VA has no formal policy promoting such a practice, it is possible that some clinicians made changes in opioid prescribing in response to information provided by the urine drug screening. However, it is also possible that cannabis use patterns by the patients changed over time. Nonetheless, short-term increases in harms in adults aged 65 and older are concerning and merit attention and further study. Our findings are consistent with those of other studies^28,29^ that have found that using multiple psychoactive agents was associated with increased risk of adverse events, particularly in older populations. Similar to other psychoactive drugs, cannabis may be associated with significant risks among older adults.^30,31^

Results from our EHR review also suggested that adults who engaged in cannabis use were more likely to engage in other adverse health behaviors, experience adverse treatment–related behaviors, and experience clinician actions related to opioid use. These findings are consistent with those of other research that has found that adults who used cannabis in the general population were more likely to use tobacco, alcohol, and other drugs and have substance use disorders and other mental health conditions.^32,33,34,35,36^ Patients engaging in concomitant cannabis and prescription opioid use are a higher-risk group and may benefit from counseling and screening for substance use disorders.

### Limitations

This study has several limitations. First, it was conducted among veterans, and our findings may not be generalizable to nonveteran populations, although older male veterans are similar to male Medicare enrollees in terms of overall comorbidities.^37,38^ Second, the success of our approach rests in part on the assumption that we have measured an adequate set of variables to account for baseline differences in health status. Adults who used cannabis were younger, had a lower prevalence of medical conditions associated with mortality, and had a higher prevalence of mental health conditions. Adults who engaged in cannabis use more commonly engaged in other substance use. Although we had access to a data source with detailed clinical data and included more than 107 variables across multiple domains of health to characterize baseline characteristics of the cohort, it is possible that we have not accounted for all confounders. In addition, given that these data were collected as part of routine care, there may be data entry errors. Third, although we used the criterion standard assessment of cannabis use (ie, biologically verified use), we had only 1 measurement of cannabis use. To respond to this potential limitation, we chose outcomes sufficiently close to our index date. Fourth, we did not have information on frequency, duration, or form of use. Fifth, while we used *ICD-9* and *ICD-10* codes to capture the most common pain diagnoses and accounted for differences in use of long-acting pain medications and daily dosage of opioids in propensity models, we did not account for differences in baseline pain scores. Sixth, we did not have data on reasons for cannabis use. Some data suggest that individuals who use medical cannabis may be different from those who use cannabis recreationally.^39,40^ However, we included variables outlined in the literature that are associated with differences between medical and recreational cannabis use (eg, race and ethnicity; tobacco, alcohol, and drug use; mental health and pain conditions; and geographic location) in the propensity model.^39,40^ Seventh, in the interest of comprehensiveness, we examined 2 outcomes at 2 time points in 2 samples. This may need to be taken into account when interpreting study findings.

## Conclusions

This cohort study found that cannabis use was associated with a small increased hazard of the composite outcome of ED visits, hospitalization, or mortality and increased short-term risks among adults aged 65 years and older receiving LTOT. Patients engaging in concomitant cannabis and prescription opioid use were a higher-risk group, and our findings suggest that they may benefit from closer monitoring, counseling, and screening for substance use disorders.

## Notes

Supplement 1. **eTable 1.** Baseline Characteristics of 297 620 Patients Receiving Any Prescription Opioid in 90 Days Before Urine Drug Screening by Cannabis Use (All Baseline Variables) **eTable 2.** Baseline Characteristics of 181 096 Adults Receiving Long-Term Prescription Opioid Therapy (>84 Days) in 90 Days Before Urine Drug Screening by Cannabis Use (All Baseline Variables) **eTable 3.** Propensity-Weighted and Matched Adults Receiving Any Prescription Opioid Therapy in Past 90 Days (Full List of Variables) **eTable 4.** Propensity-Weighted and Matched Adults Receiving Long-Term (>84 Days) Prescription Opioid Therapy in Past 90 Days (Full List Of Variables) **eFigure 1.** Survival Probability Among Adults Followed up for 180 Days Receiving Prescription Opioid Therapy in Prior 90 Days by Cannabis Use **eFigure 2.** Probability of Emergency Department Visits, Hospitalization, or Mortality Among Adults Followed up for 180 Days Receiving Any Prescription Opioid Therapy by Cannabis Use **eTable 5.** Association of Cannabis Use With 90- and 180-Day Adverse Events Among Adults Receiving Prescription Opioids (Propensity Matching Approach) **eTable 6.** Baseline Characteristics of 135 413 Adults Aged 65 Years and Older Receiving Any Prescription Opioid in 90 Days Before Urine Drug Screening by Cannabis Use (All Baseline Variables) **eTable 7.** Baseline Characteristics of 77 791 Adults Aged 65 Years and Older Receiving Long-Term Prescription Opioid Therapy (>84 Days) in 90 Days Before Urine Drug Screening by Cannabis Use (All Baseline Variables) **eTable 8.** Propensity-Weighted and Matched Adults Aged 65 Years and Older Receiving Any Prescription Opioid Therapy in Past 90 Days (Full List of Variables) **eTable 9.** Propensity-Weighted and Matched Veterans Aged 65 Years and Older Receiving Long-Term (>84 Days) Prescription Opioid Therapy in Past 90 Days (Full List of Variables) **eFigure 3.** Survival Probability Among Adults Ages 65 Years and Older Followed up for 180 Days Receiving Prescription Opioid Therapy in Prior 90 Days By Cannabis Use **eFigure 4.** Probability of Emergency Department Visits, Hospitalization, or Mortality Among Adults Aged 65 Years and Older Followed up for 180 Days Receiving Any Prescription Opioid Therapy By Cannabis Use **eTable 10.** Association of Cannabis Use With 90- and 180-Day Adverse Events Among Adults Ages 65 Years and Older Receiving Prescription Opioids (Propensity-Weighting and Matching Approach) **eTable 11.** Abstraction Tool for Chart Review **eTable 12.** High-Risk Behaviors, Adverse Outcomes, and Clinician Actions Associated With Opioid Use Among Adults Aged 65 Years and Older

Supplement 2.
Data Sharing Statement
