# Supplementary material for: Association of a Positive Drug Screening for Cannabis With Mortality and Hospital Visits Among Veterans Affairs Enrollees Prescribed Opioids
Source: JAMA Netw Open. 2022 Dec 16;5(12):e2247201. doi: 10.1001/jamanetworkopen.2022.47201 (PMC9856228; doi:10.1001/jamanetworkopen.2022.47201)
Supplement: Supplement 1. — eTable 1. Baseline Characteristics of 297 620 Patients Receiving Any Prescription Opioid in 90 Days Before Urine Drug Screening by Cannabis Use (All Baseline Variables) eTable 2. Baseline Characteristics of 181 096 Adults Receiving Long-Term Prescription Opioid Therapy (>84 Days) in 90 Days Before Urine Drug Screening by Cannabis Use (All Baseline Variables) eTable 3. Propensity-Weighted and Matched Adults Receiving Any Prescription Opioid Therapy in Past 90 Days (Full List of Variables) eTable 4. Propensity-Weighted and Matched Adults Receiving Long-Term (>84 Days) Prescription Opioid Therapy in Past 90 Days (Full List Of Variables) eFigure 1. Survival Probability Among Adults Followed up for 180 Days Receiving Prescription Opioid Therapy in Prior 90 Days by Cannabis Use eFigure 2. Probability of Emergency Department Visits, Hospitalization, or Mortality Among Adults Followed up for 180 Days Receiving Any Prescription Opioid Therapy by Cannabis Use eTable 5. Association of Cannabis Use With 90- and 180-Day Adverse Events Among Adults Receiving Prescription Opioids (Propensity Matching Approach) eTable 6. Baseline Characteristics of 135 413 Adults Aged 65 Years and Older Receiving Any Prescription Opioid in 90 Days Before Urine Drug Screening by Cannabis Use (All Baseline Variables) eTable 7. Baseline Characteristics of 77 791 Adults Aged 65 Years and Older Receiving Long-Term Prescription Opioid Therapy (>84 Days) in 90 Days Before Urine Drug Screening by Cannabis Use (All Baseline Variables) eTable 8. Propensity-Weighted and Matched Adults Aged 65 Years and Older Receiving Any Prescription Opioid Therapy in Past 90 Days (Full List of Variables) eTable 9. Propensity-Weighted and Matched Veterans Aged 65 Years and Older Receiving Long-Term (>84 Days) Prescription Opioid Therapy in Past 90 Days (Full List of Variables) eFigure 3. Survival Probability Among Adults Ages 65 Years and Older Followed up for 180 Days Receiving Prescription Opioid Therapy in Prior 90 Days B [file jamanetwopen-e2247201-s001.pdf]

Supplemental Online Content

Keyhani S, Leonard S, Byers AL, et al. Association of a positive drug screening for cannabis with mortality and hospital visits among Veterans Affairs enrollees prescribed opioids. *JAMA Netw Open.* 2022;5(12):e2247201. doi:10.1001/jamanetworkopen.2022.47201

eTable 1. Baseline Characteristics of 297 620 Patients Receiving Any Prescription Opioid in 90 Days Before Urine Drug Screening by Cannabis Use (All Baseline Variables)

eTable 2. Baseline Characteristics of 181 096 Adults Receiving Long-Term Prescription Opioid Therapy (>84 Days) in 90 Days Before Urine Drug Screening by Cannabis Use (All Baseline Variables)

eTable 3. Propensity-Weighted and Matched Adults Receiving Any Prescription Opioid Therapy in Past 90 Days (Full List of Variables)

eTable 4. Propensity-Weighted and Matched Adults Receiving Long-Term (>84 Days) Prescription Opioid Therapy in Past 90 Days (Full List Of Variables)

eFigure 1. Survival Probability Among Adults Followed up for 180 Days Receiving Prescription Opioid Therapy in Prior 90 Days by Cannabis Use

eFigure 2. Probability of Emergency Department Visits, Hospitalization, or Mortality Among Adults Followed up for 180 Days Receiving Any Prescription Opioid Therapy by Cannabis Use

eTable 5. Association of Cannabis Use With 90- and 180-Day Adverse Events Among Adults Receiving Prescription Opioids (Propensity Matching Approach)

eTable 6. Baseline Characteristics of 135 413 Adults Aged 65 Years and Older Receiving Any Prescription Opioid in 90 Days Before Urine Drug Screening by Cannabis Use (All Baseline Variables)

eTable 7. Baseline Characteristics of 77 791 Adults Aged 65 Years and Older Receiving Long-Term Prescription Opioid Therapy (>84 Days) in 90 Days Before Urine Drug Screening by Cannabis Use (All Baseline Variables)

eTable 8. Propensity-Weighted and Matched Adults Aged 65 Years and Older Receiving Any Prescription Opioid Therapy in Past 90 Days (Full List of Variables)

eTable 9. Propensity-Weighted and Matched Veterans Aged 65 Years and Older Receiving Long-Term (>84 Days) Prescription Opioid Therapy in Past 90 Days (Full List of Variables)

eFigure 3. Survival Probability Among Adults Ages 65 Years and Older Followed up for 180 Days Receiving Prescription Opioid Therapy in Prior 90 Days By Cannabis Use

© 2022 Keyhani S et al. *JAMA Network Open.*

eFigure 4. Probability of Emergency Department Visits, Hospitalization, or Mortality Among Adults Aged 65 Years and Older Followed up for 180 Days Receiving Any Prescription Opioid Therapy By Cannabis Use

eTable 10. Association of Cannabis Use With 90- and 180-Day Adverse Events Among Adults Ages 65 Years and Older Receiving Prescription Opioids (Propensity-Weighting and Matching Approach)

eTable 11. Abstraction Tool for Chart Review

eTable 12. High-Risk Behaviors, Adverse Outcomes, and Clinician Actions Associated With Opioid Use Among Adults Aged 65 Years and Older

This supplemental material has been provided by the authors to give readers additional information about their work.

© 2022 Keyhani S et al. *JAMA Network Open.*

eTable 1. Baseline Characteristics of 297 620 Patients Receiving Any Prescription Opioid in 90 Days Before Urine Drug Screening by Cannabis Use (All Baseline Variables)*

	Non-use	Cannabis use	P-value
N	267106	30514	
Age (SE)	62.33 (12.34)	57.87 (10.56)	<0.001
Male	247684 (92.7)	28784 (94.3)	<0.001
Female	19422 (7.3)	1730 (5.7)	<0.001
Married	144741 (54.2)	11948 (39.2)	<0.001
**Race**			
White	212316 (79.5)	23076 (75.6)	<0.001
Black or African American	33719 (12.6)	4686 (15.4)	<0.001
Asian	930 (0.3)	110 (0.4)	0.77
Native Hawaiian/Pacific Islander	2328 (0.9)	274 (0.9)	0.66
American Indian or Alaska Native	3021 (1.1)	481 (1.6)	<0.001
Race unknown	14792 (5.5)	1887 (6.2)	<0.001
**Hispanic**	9901 (3.7)	1321 (4.3)	<0.001
**Clinical Conditions**			
Hypertension	184938 (69.2)	17351 (56.9)	<0.001
Hyperlipidemia	163767 (61.3)	14297 (46.9)	<0.001
Diabetes	93990 (35.2)	6973 (22.9)	<0.001
Stroke	6949 (2.6)	586 (1.9)	<0.001
Transient Ischemic Attack	4830 (1.8)	302 (1.0)	<0.001
Paralytic syndromes	7132 (2.7)	755 (2.5)	0.046
Ischemic heart disease	65171 (24.4)	4546 (14.9)	<0.001
Severe ischemic heart disease	13178 (4.9)	994 (3.3)	<0.001
Myocardial infarction	19039 (7.1)	1539 (5.0)	<0.001
Coronary Artery Bypass Graft	1661 (0.6)	168 (0.6)	0.14
Percutaneous Coronary Intervention	5151 (1.9)	418 (1.4)	<0.001
Peripheral vascular disease	10167 (3.8)	999 (3.3)	<0.001
Abdominal aortic aneurysm	7089 (2.7)	516 (1.7)	<0.001
Heart Failure (CHF)	26014 (9.7)	1573 (5.2)	<0.001
Severe CHF	4562 (1.7)	287 (0.9)	<0.001

© 2022 Keyhani S et al. *JAMA Network Open.*

	Non-use	Cannabis use	P-value
Defibrillator	1273 (0.5)	88 (0.3)	<0.001
Atrial fibrillation	24898 (9.3)	1570 (5.1)	<0.001
Cardiac arrhythmia	21335 (8.0)	1601 (5.2)	<0.001
Chronic Kidney Disease (CKD)	38420 (14.4)	2405 (7.9)	<0.001
Severe CKD	2765 (1.0)	147 (0.5)	<0.001
Dialysis	847 (0.3)	61 (0.2)	0.001
Asthma	16978 (6.4)	1573 (5.2)	<0.001
Bronchiectasis	820 (0.3)	38 (0.1)	<0.001
Pulmonary embolism	4275 (1.6)	315 (1.0)	<0.001
Deep Venous Thrombosis	6330 (2.4)	515 (1.7)	<0.001
Cirrhosis	8488 (3.2)	1175 (3.9)	<0.001
Decompensated cirrhosis	2421 (0.9)	342 (1.1)	<0.001
Hepatitis	5380 (2.0)	867 (2.8)	<0.001
Parkinson’s Disease	2554 (1.0)	121 (0.4)	<0.001
Extrapyramidal disease	10424 (3.9)	785 (2.6)	<0.001
Multiple Sclerosis	1195 (0.4)	161 (0.5)	0.054
Seizure	4472 (1.7)	611 (2.0)	<0.001
Falls	10718 (4.0)	917 (3.0)	<0.001
Orthostatic hypotension	3441 (1.3)	220 (0.7)	<0.001
Rheumatoid arthritis	6989 (2.6)	590 (1.9)	<0.001
Other inflammatory conditions	11269 (4.2)	922 (3.0)	<0.001
AIDS	1144 (0.4)	287 (0.9)	<0.001
Defibrillator	1273 (0.5)	88 (0.3)	<0.001
External lung disease	7560 (2.8)	1199 (3.9)	<0.001
Bronchitis	4994 (1.9)	403 (1.3)	<0.001
Lung resection	634 (0.2)	85 (0.3)	0.18
Influenza	1942 (0.7)	128 (0.4)	<0.001
Tracheostomy	1069 (0.4)	80 (0.3)	<0.001
Upper respiratory	25540 (9.6)	1849 (6.1)	<0.001
Ventilation	18967 (7.1)	1364 (4.5)	<0.001
Necrotic lung	773 (0.3)	122 (0.4)	0.001

© 2022 Keyhani S et al. *JAMA Network Open.*

	Non-use	Cannabis use	P-value
Respiratory failure	17525 (6.6)	1326 (4.3)	<0.001
Home oxygen within one year	8153 (3.1)	485 (1.6)	<0.001
Chronic Obstructive Lung Disease (COPD)	58882 (22.0)	5775 (18.9)	<0.001
Severe COPD	6967 (2.6)	727 (2.4)	0.020
Pulmonary fibrosis	2695 (1.0)	143 (0.5)	<0.001
Pneumonia	16968 (6.4)	1290 (4.2)	<0.001
Sleep apnea	38746 (14.5)	2618 (8.6)	<0.001
Dementia	19662 (7.4)	1383 (4.5)	<0.001
**Cancer**			
Head and neck	2151 (0.8)	310 (1.0)	<0.001
Respiratory tract	5696 (2.1)	627 (2.1)	0.38
Gastrointestinal tract	3466 (1.3)	358 (1.2)	0.072
Other Gastrointestinal tract	1875 (0.7)	333 (1.1)	<0.001
Skin	11094 (4.2)	671 (2.2)	<0.001
Genitourinary	5643 (2.1)	517 (1.7)	<0.001
Prostate	12741 (4.8)	973 (3.2)	<0.001
Central Nervous System	489 (0.2)	64 (0.2)	0.34
Bone limb connective tissue	1724 (0.6)	135 (0.4)	<0.001
Lymphoma/Leukemia	3172 (1.2)	319 (1.0)	0.031
Other Cancer	5967 (2.2)	791 (2.6)	<0.001
Charlson comorbidity index	3.33 (2.34)	2.50 (1.91)	<0.001
Functional impairment	37197 (13.9)	3958 (13.0)	<0.001
**Body Mass Index (BMI)**			<0.001
High BMI	134052 (50.2)	10883 (35.7)	
Middle BMI	85045 (31.8)	10630 (34.8)	
Low BMI	45068 (16.9)	8664 (28.4)	
Unknown BMI	2941 (1.1)	337 (1.1)	
**Systolic blood pressure**			<0.001
Systolic blood pressure < 100	5525 (2.1)	699 (2.3)	
Systolic blood pressure 100 - 119.9	56481 (21.1)	6613 (21.7)	
Systolic blood pressure 120 - 139.9	127304 (47.7)	14194 (46.5)	

© 2022 Keyhani S et al. *JAMA Network Open.*

	Non-use	Cannabis use	P-value
Systolic blood pressure 140 - 149.9	34879 (13.1)	3832 (12.6)	
Systolic blood pressure 150 - 159.9	20008 (7.5)	2295 (7.5)	
Systolic blood pressure 160 - 179.9	14984 (5.6)	1838 (6.0)	
Systolic blood pressure ≥ 180	3332 (1.2)	421 (1.4)	
Systolic blood pressure missing	4593 (1.7)	622 (2.0)	
**Albumin**			<0.001
Albumin < 2.5	367 (0.1)	63 (0.2)	
Albumin 2.5 to 3.5	9909 (3.7)	996 (3.3)	
Albumin > 3.5	83417 (31.2)	8994 (29.5)	
Albumin missing	173413 (64.9)	20461 (67.1)	
**Mental Health Conditions**			
Psychosis	7298 (2.7)	1067 (3.5)	<0.001
Depression	95580 (35.8)	11659 (38.2)	<0.001
Bipolar	9724 (3.6)	1701 (5.6)	<0.001
PTSD	54253 (20.3)	7312 (24.0)	<0.001
Anxiety	52693 (19.7)	6252 (20.5)	0.002
Self-harm	585 (0.2)	131 (0.4)	<0.001
**Health Behaviors**			
Opioid use disorder/dependence	7881 (3.0)	1291 (4.2)	<0.001
Benzodiazepines use disorder/dependence	984 (0.4)	178 (0.6)	<0.001
Amphetamine use disorder/dependence	1852 (0.7)	488 (1.6)	<0.001
Other drug use disorder use disorder/dependence	5096 (1.9)	1412 (4.6)	<0.001
Alcohol use disorder	17246 (6.5)	3467 (11.4)	<0.001
Alcohol abuse according to audit C score	10130 (3.8)	2073 (6.8)	<0.001
Current tobacco use	106201 (39.8)	16863 (55.3)	<0.001
Positive amphetamine lab	4533 (1.7)	935 (3.1)	<0.001
Positive benzodiazepine lab	30192 (11.3)	4201 (13.8)	<0.001
Positive cocaine lab	1742 (0.7)	693 (2.3)	<0.001
**Pain Disorders**			
Back and spine disorders	175145 (65.6)	18826 (61.7)	<0.001

© 2022 Keyhani S et al. *JAMA Network Open.*

	Non-use	Cannabis use	P-value
Neck and spine disorders	59197 (22.2)	6747 (22.1)	0.84
Osteoarthritis	95523 (35.8)	8303 (27.2)	<0.001
Neuropathy	53123 (19.9)	4116 (13.5)	<0.001
Headache	41002 (15.4)	5563 (18.2)	<0.001
Traumatic brain injury	453 (0.2)	53 (0.2)	0.93
**Social Risk**			
Homelessness/marginal housing	10594 (4.0)	2330 (7.6)	<0.001
Lack of social support	126 (0.0)	20 (0.1)	0.22
**VA Priority group**			<0.001
VA Priority group 1	54175 (20.3)	5770 (18.9)	
VA Priority group 2	18367 (6.9)	1889 (6.2)	
VA Priority group 3	26751 (10.0)	3162 (10.4)	
VA Priority group 4	4219 (1.6)	432 (1.4)	
VA Priority group 5	76760 (28.7)	11187 (36.7)	
VA Priority group 6	6700 (2.5)	661 (2.2)	
VA Priority group 7	3995 (1.5)	436 (1.4)	
VA Priority group 8	27516 (10.3)	2275 (7.5)	
VA Priority unknown	48623 (18.2)	4702 (15.4)	
**Utilization in past year**			
ICU visits within six months			<0.001
ICU visits within six month (none)	260694 (97.6)	29972 (98.2)	
ICU visits within six months (one)	5531 (2.1)	471 (1.5)	
ICU visits within six months (two or more)	881 (0.3)	71 (0.2)	
ICU visits within one year			<0.001
ICU visits within one year (none)	255891 (95.8)	29618 (97.1)	
ICU visits within one year (one)	9163 (3.4)	728 (2.4)	
ICU visits within one year (two or more)	2052 (0.8)	168 (0.6)	
**Hospitalized within one year**	34547 (12.9)	4084 (13.4)	0.027
ED visits within six months			<0.001
ED visits within six months (none)	196955 (73.7)	21606 (70.8)	
ED visits within six months (one)	38742 (14.5)	4930 (16.2)	

© 2022 Keyhani S et al. *JAMA Network Open.*

	Non-use	Cannabis use	P-value
ED visits within six months (two or more)	31409 (11.8)	3978 (13.0)	
ED visits within one year			<0.001
ED visits within one year (none)	169201 (63.3)	18395 (60.3)	
ED visits within one year (one)	44473 (16.6)	5627 (18.4)	
ED visits within one year (two or more)	53432 (20.0)	6492 (21.3)	
VA visit count	33.48 (24.43)	31.30 (23.35)	<0.001
**Psychoactive Drugs in past year**			
Morphine Equivalents over prior 90-day period	188.3 (447.5)	194.2 (456.7)	<0.001
Long-acting opioid	59696 (22.3)	6476 (21.2)	<0.001
Alcohol use disorder treatment medication	262 (0.1)	53 (0.2)	<0.001
Benzodiazepines	56294 (21.1)	6604 (21.6)	0.022
Gaba drugs	72909 (27.3)	7188 (23.6)	<0.001
Muscle relaxants	55865 (20.9)	6668 (21.9)	<0.001
Antidepressants	87747 (32.9)	9913 (32.5)	0.20
Antipsychotics	18379 (6.9)	2691 (8.8)	<0.001
Sedatives	613 (0.2)	61 (0.2)	0.33
**Number of psychoactive drugs in past year**			0.26
1	96167 (36.0)	11145 (36.5)	
2	87090 (32.6)	9796 (32.1)	
3	54189 (20.3)	6230 (20.4)	
4	23006 (8.6)	2580 (8.5)	
5 or more	6654 (2.5)	763 (2.5)	
**Care Assessment Need (CAN) Score**			<0.001
CAN Score < 10	18724 (7.0)	2019 (6.6)	
CAN Score 10 - 19.9	21134 (7.9)	2733 (9.0)	
CAN Score 20 - 29.9	21596 (8.1)	3015 (9.9)	
CAN Score 30 - 39.9	26031 (9.7)	3700 (12.1)	
CAN Score 40 - 49.9	27679 (10.4)	3819 (12.5)	
CAN Score 50 - 59.9	28978 (10.8)	3774 (12.4)	
CAN Score 60 - 69.9	28781 (10.8)	3372 (11.1)	
CAN Score 70 - 79.9	31566 (11.8)	3231 (10.6)	

© 2022 Keyhani S et al. *JAMA Network Open.*

	Non-use	Cannabis use	P-value
CAN Score 80 - 89.9	29843 (11.2)	2472 (8.1)	
CAN Score 90 - 98.9	26253 (9.8)	1522 (5.0)	
CAN Score Missing	6521 (2.4)	857 (2.8)	
**Year Index Urine Drug Screen was ordered**			
2014	159276 (59.6)	16400 (53.7)	<0.001
2015	61051 (22.9)	7518 (24.6)	<0.001
2016	25485 (9.5)	3451 (11.3)	<0.001
2017	11244 (4.2)	1633 (5.4)	<0.001
2018	6623 (2.5)	928 (3.0)	<0.001
2019	3427 (1.3)	584 (1.9)	<0.001

* Facility variable not shown given that 130 facilities were included in the analysis

© 2022 Keyhani S et al. *JAMA Network Open.*

eTable 2. Baseline Characteristics of 181 096 Adults Receiving Long-Term Prescription Opioid Therapy (>84 Days) in 90 Days Before Urine Drug Screening by Cannabis Use (All Baseline Variables)*

	Non-use	Cannabis Use	P-value
N	162695	18401	
Age (SE)	61.9 (11.61)	58.4 (9.53)	<0.001
Male	151486 (93.1)	17449 (94.8)	<0.001
Female	11209 (6.9)	952 (5.2)	<0.001
Married	85944 (52.8)	7173 (39.0)	<0.001
**Race**			
White	131206 (80.6)	14326 (77.9)	<0.001
Black or African American	19014 (11.7)	2406 (13.1)	<0.001
Asian	436 (0.3)	59 (0.3)	0.22
Native Hawaiian/Pacific Islander	1377 (0.8)	161 (0.9)	0.72
American Indian or Alaska Native	1870 (1.1)	298 (1.6)	<0.001
Race unknown	8792 (5.4)	1151 (6.3)	<0.001
**Hispanic**	5442 (3.3)	722 (3.9)	<0.001
**Clinical Conditions**			
Hypertension	113270 (69.6)	10785 (58.6)	<0.001
Hyperlipidemia	100185 (61.6)	8985 (48.8)	<0.001
Diabetes	56748 (34.9)	4325 (23.5)	<0.001
Stroke	3925 (2.4)	351 (1.9)	<0.001
Transient Ischemic Attack	2883 (1.8)	182 (1.0)	<0.001
Paralytic syndromes	4420 (2.7)	492 (2.7)	0.75
Ischemic heart disease	38776 (23.8)	2792 (15.2)	<0.001
Severe ischemic heart disease	7104 (4.4)	512 (2.8)	<0.001
Myocardial infarction	11337 (7.0)	917 (5.0)	<0.001
Coronary Artery Bypass Graft	892 (0.5)	88 (0.5)	0.24
Percutaneous Coronary Intervention	3018 (1.9)	244 (1.3)	<0.001
Peripheral vascular disease	5462 (3.4)	504 (2.7)	<0.001
Abdominal aortic aneurysm	4163 (2.6)	290 (1.6)	<0.001
Heart Failure (CHF)	15122 (9.3)	938 (5.1)	<0.001
Severe CHF	2339 (1.4)	136 (0.7)	<0.001

© 2022 Keyhani S et al. *JAMA Network Open.*

	Non-use	Cannabis Use	P-value
Defibrillator	736 (0.5)	50 (0.3)	0.001
Atrial fibrillation	13843 (8.5)	913 (5.0)	<0.001
Cardiac arrhythmia	12410 (7.6)	945 (5.1)	<0.001
Chronic Kidney Disease (CKD)	21834 (13.4)	1456 (7.9)	<0.001
Severe CKD	1416 (0.9)	79 (0.4)	<0.001
Dialysis	438 (0.3)	29 (0.2)	0.006
Asthma	10335 (6.4)	982 (5.3)	<0.001
Bronchiectasis	510 (0.3)	17 (0.1)	<0.001
Pulmonary embolism	2505 (1.5)	186 (1.0)	<0.001
Deep Venous Thrombosis	3601 (2.2)	299 (1.6)	<0.001
Cirrhosis	5273 (3.2)	738 (4.0)	<0.001
Decompensated cirrhosis	1424 (0.9)	211 (1.1)	<0.001
Hepatitis	2706 (1.7)	368 (2.0)	0.001
Parkinson's Disease	1411 (0.9)	66 (0.4)	<0.001
Extrapyramidal disease	6243 (3.8)	511 (2.8)	<0.001
Multiple Sclerosis	739 (0.5)	99 (0.5)	0.13
Seizure	2768 (1.7)	381 (2.1)	<0.001
Falls	6148 (3.8)	529 (2.9)	<0.001
Orthostatic hypotension	1978 (1.2)	126 (0.7)	<0.001
Rheumatoid arthritis	4346 (2.7)	370 (2.0)	<0.001
Other inflammatory conditions	7223 (4.4)	576 (3.1)	<0.001
AIDS	758 (0.5)	179 (1.0)	<0.001
Defibrillator	736 (0.5)	50 (0.3)	0.001
External lung disease	4378 (2.7)	658 (3.6)	<0.001
Bronchitis	2929 (1.8)	232 (1.3)	<0.001
Lung resection	369 (0.2)	48 (0.3)	0.41
Influenza	1106 (0.7)	83 (0.5)	<0.001
Tracheostomy	624 (0.4)	40 (0.2)	0.001
Upper respiratory	15527 (9.5)	1118 (6.1)	<0.001
Ventilation	10908 (6.7)	774 (4.2)	<0.001
Necrotic lung	481 (0.3)	75 (0.4)	0.011

© 2022 Keyhani S et al. *JAMA Network Open.*

	Non-use	Cannabis Use	P-value
Respiratory failure	10678 (6.6)	811 (4.4)	<0.001
Home oxygen within one year	4989 (3.1)	318 (1.7)	<0.001
Chronic Obstructive Lung Disease (COPD)	37815 (23.2)	3762 (20.4)	<0.001
Severe COPD	4063 (2.5)	408 (2.2)	0.022
Pulmonary fibrosis	1575 (1.0)	79 (0.4)	<0.001
Pneumonia	10543 (6.5)	816 (4.4)	<0.001
Sleep apnea	22711 (14.0)	1551 (8.4)	<0.001
Dementia	11856 (7.3)	878 (4.8)	<0.001
**Cancer**			
Head and neck	1310 (0.8)	187 (1.0)	0.003
Respiratory tract	3396 (2.1)	375 (2.0)	0.68
Gastrointestinal tract	1981 (1.2)	209 (1.1)	0.35
Other Gastrointestinal tract	1045 (0.6)	179 (1.0)	<0.001
Skin	6142 (3.8)	410 (2.2)	<0.001
Genitourinary	3296 (2.0)	309 (1.7)	0.002
Prostate	7085 (4.4)	553 (3.0)	<0.001
Central Nervous System	263 (0.2)	37 (0.2)	0.25
Bone limb connective tissue	969 (0.6)	79 (0.4)	0.006
Lymphoma/Leukemia	1682 (1.0)	189 (1.0)	0.96
Other Cancer	3357 (2.1)	461 (2.5)	<0.001
Charlson comorbidity index	3.25 (2.26)	2.55 (1.85)	<0.001
Functional impairment	22902 (14.1)	2393 (13.0)	<0.001
**Body Mass Index (BMI)**			<0.001
High BMI	80721 (49.6)	6593 (35.8)	
Middle BMI	51815 (31.8)	6389 (34.7)	
Low BMI	28653 (17.6)	5274 (28.7)	
Unknown BMI	1506 (0.9)	145 (0.8)	
**Systolic blood pressure**			<0.001
Systolic blood pressure< 100	3413 (2.1)	447 (2.4)	
Systolic blood pressure 100 - 119.9	34451 (21.2)	3974 (21.6)	
Systolic blood pressure 120 - 139.9	78030 (48.0)	8668 (47.1)	

© 2022 Keyhani S et al. *JAMA Network Open.*

	Non-use	Cannabis Use	P-value
Systolic blood pressure 140 - 149.9	21007 (12.9)	2264 (12.3)	
Systolic blood pressure 150 - 159.9	12090 (7.4)	1332 (7.2)	
Systolic blood pressure 160 - 179.9	9012 (5.5)	1101 (6.0)	
Systolic blood pressure ≥ 180	1977 (1.2)	229 (1.2)	
Systolic blood pressure missing	2715 (1.7)	386 (2.1)	
**Albumin**			<0.001
Albumin < 2.5	183 (0.1)	28 (0.2)	
Albumin 2.5 to 3.5	5670 (3.5)	537 (2.9)	
Albumin > 3.5	51788 (31.8)	5454 (29.6)	
Albumin missing	105054 (64.6)	12382 (67.3)	
**Mental Health Conditions**			
Psychosis	4775 (2.9)	655 (3.6)	<0.001
Depression	61776 (38.0)	7437 (40.4)	<0.001
Bipolar	6509 (4.0)	1071 (5.8)	<0.001
PTSD	34054 (20.9)	4483 (24.4)	<0.001
Anxiety	34371 (21.1)	3935 (21.4)	0.42
Self-harm	397 (0.2)	82 (0.4)	<0.001
**Health Behaviors**			
Opioid use disorder/dependence	5773 (3.5)	872 (4.7)	<0.001
Benzodiazepines use disorder/dependence	686 (0.4)	102 (0.6)	0.011
Amphetamine use disorder/dependence	1236 (0.8)	283 (1.5)	<0.001
Other drug use disorder use disorder/dependence	3479 (2.1)	853 (4.6)	<0.001
Alcohol use disorder	10660 (6.6)	1983 (10.8)	<0.001
Elevated audit C score	5605 (3.4)	1040 (5.7)	<0.001
Current tobacco use	70764 (43.5)	10485 (57.0)	<0.001
Positive amphetamine lab	2883 (1.8)	543 (3.0)	<0.001
Positive benzodiazepine lab	20450 (12.6)	2737 (14.9)	<0.001
Positive cocaine lab	990 (0.6)	334 (1.8)	<0.001
**Pain Disorders**			
Back and spine disorders	111939 (68.8)	12108 (65.8)	<0.001
Neck and spine disorders	38103 (23.4)	4346 (23.6)	0.55

© 2022 Keyhani S et al. *JAMA Network Open.*

	Non-use	Cannabis Use	P-value
Osteoarthritis	58710 (36.1)	5282 (28.7)	<0.001
Neuropathy	32820 (20.2)	2587 (14.1)	<0.001
Headache	25485 (15.7)	3342 (18.2)	<0.001
Traumatic brain injury	277 (0.2)	33 (0.2)	0.85
**Social Risk**			
Homelessness/marginal housing	6418 (3.9)	1320 (7.2)	<0.001
Lack of social support	43 (0.0)	10 (0.1)	0.061
**VA Priority group**			<0.001
VA Priority group 1	32935 (20.2)	3459 (18.8)	
VA Priority group 2	10938 (6.7)	1103 (6.0)	
VA Priority group 3	15968 (9.8)	1896 (10.3)	
VA Priority group 4	2779 (1.7)	295 (1.6)	
VA Priority group 5	49250 (30.3)	6926 (37.6)	
VA Priority group 6	3496 (2.1)	312 (1.7)	
VA Priority group 7	2130 (1.3)	227 (1.2)	
VA Priority group 8	15634 (9.6)	1333 (7.2)	
VA Priority unknown	29565 (18.2)	2850 (15.5)	
**Utilization in past year**			
ICU visits within six months			<0.001
ICU visits within six months (none)	159221 (97.9)	18098 (98.4)	
ICU visits within six months (one)	3043 (1.9)	258 (1.4)	
ICU visits within six months (two or more)	431 (0.3)	45 (0.2)	
ICU visits within one year			<0.001
ICU visits within one year (none)	156307 (96.1)	17885 (97.2)	
ICU visits within one year (one)	5246 (3.2)	415 (2.3)	
ICU visits within one year (two or more)	1142 (0.7)	101 (0.5)	
**Hospitalized within one year**	19894 (12.2)	2243 (12.2)	0.89
ED visits within six months			0.002
ED visits within six months (none)	123503 (75.9)	14015 (76.2)	
ED visits within six months (one)	22353 (13.7)	2623 (14.3)	
ED visits within six months (two or more)	16839 (10.4)	1763 (9.6)	

© 2022 Keyhani S et al. *JAMA Network Open.*

	Non-use	Cannabis Use	P-value
ED visits within one year			<0.001
ED visits within one year (none)	105298 (64.7)	11891 (64.6)	
ED visits within one year (one)	26746 (16.4)	3199 (17.4)	
ED visits within one year (two or more)	30651 (18.8)	3311 (18.0)	
VA visit count	34.90 (24.08)	32.57 (23.27)	<0.001
**Psychoactive Drugs in past year**			
Morphine Equivalents over prior 90-day period	268.6 (542.7)	277.6 (561.3)	0.03
Long-acting opioid	51385 (31.6)	5534 (30.1)	<0.001
Alcohol use disorder treatment medication	130 (0.1)	30 (0.2)	0.001
Benzodiazepines	39106 (24.0)	4549 (24.7)	0.040
Gaba drugs	46675 (28.7)	4496 (24.4)	<0.001
Muscle relaxants	35051 (21.5)	3983 (21.6)	0.76
Antidepressants	56380 (34.7)	6310 (34.3)	0.33
Antipsychotics	12451 (7.7)	1769 (9.6)	<0.001
Sedatives	285 (0.2)	27 (0.1)	0.43
**Number of psychoactive drugs in past year**			0.13
1	53996 (33.2)	6262 (34.0)	
2	53193 (32.7)	5942 (32.3)	
3	35069 (21.6)	3968 (21.6)	
4	15664 (9.6)	1715 (9.3)	
5 or more	4773 (2.9)	514 (2.8)	
**Care Assessment Need (CAN) score**			<0.001
CAN Score < 10	10833 (6.7)	1061 (5.8)	
CAN Score 10 - 19.9	12812 (7.9)	1527 (8.3)	
CAN Score 20 - 29.9	13289 (8.2)	1754 (9.5)	
CAN Score 30 - 39.9	16339 (10.0)	2249 (12.2)	
CAN Score 40 - 49.9	17484 (10.7)	2410 (13.1)	
CAN Score 50 - 59.9	18410 (11.3)	2417 (13.1)	
CAN Score 60 - 69.9	18186 (11.2)	2173 (11.8)	
CAN Score 70 - 79.9	19796 (12.2)	2097 (11.4)	
CAN Score 80 - 89.9	18057 (11.1)	1556 (8.5)	

© 2022 Keyhani S et al. *JAMA Network Open.*

	Non-use	Cannabis Use	P-value
CAN Score 90 - 98.9	15005 (9.2)	886 (4.8)	
CAN Score Missing	2484 (1.5)	271 (1.5)	
**Year Index Urine Drug Screen was ordered**			<0.001
2014	109971 (67.6)	11264 (61.2)	
2015	34211 (21.0)	4521 (24.6)	
2016	11747 (7.2)	1637 (8.9)	
2017	3982 (2.4)	563 (3.1)	
2018	2110 (1.3)	295 (1.6)	
2019	674 (0.4)	121 (0.7)	

* Facility level variable not shown

© 2022 Keyhani S et al. *JAMA Network Open.*

eTable 3. Propensity-Weighted and Matched Adults Receiving Any Prescription Opioid Therapy in Past 90 Days (Full List of Variables)

	Propensity Weighted			Propensity Matched		
	**Non-use**	**Cannabis use**	**Diff weighted**	**Non-use**	**Cannabis use**	**Diff matched**
N	30923.26	30514		30514	30514	
Age	57.84 (10.51)	57.87 (10.56)	0.003	57.62 (12.37)	57.87 (10.56)	0.022
Male	29179 (94.36)	28784 (94.33)	0	28702 (94.06)	28784 (94.33)	0.011
Female	1744 (5.64)	1730 (5.67)	0	1,812 (5.94)	1,730 (5.67)	-0.011
Married	11896 (38.47)	11948 (39.16)	0.007	11927 (39.09)	11948 (39.16)	0.001
**Race**						
White	23239 (75.15)	23076 (75.62)	0.005	23046 (75.53)	23076 (75.62)	0.002
Black or African American	4891 (15.82)	4686 (15.36)	-0.005	4682 (15.34)	4686 (15.36)	0
Asian	112 (0.36)	110 (0.36)	0	108 (0.35)	110 (0.36)	0.001
Native Hawaiian or other Pacific Islander	279 (0.9)	274 (0.9)	0	276 (0.9)	274 (0.9)	-0.001
American Indian or Alaska Native	488 (1.58)	481 (1.58)	0	479 (1.57)	481 (1.58)	0.001
Race unknown	1915 (6.19)	1887 (6.18)	0	1923 (6.3)	1887 (6.18)	-0.005
**Hispanic**	1358 (4.39)	1321 (4.33)	-0.001	1321 (4.33)	1321 (4.33)	0
**Clinical Conditions**						
Hypertension	17581 (56.86)	17351 (56.86)	0	17341 (56.83)	17351 (56.86)	0.001
Hyperlipidemia	14337 (46.36)	14297 (46.85)	0.005	14337 (46.98)	14297 (46.85)	-0.003
Diabetes	7012 (22.68)	6973 (22.85)	0.002	6887 (22.57)	6973 (22.85)	0.007
Stroke	602 (1.95)	586 (1.92)	0	596 (1.95)	586 (1.92)	-0.002
Transient Ischemic Attack	306 (0.99)	302 (0.99)	0	299 (0.98)	302 (0.99)	0.001
Paralytic syndromes	773 (2.5)	755 (2.47)	0	772 (2.53)	755 (2.47)	-0.004
Ischemic heart disease	4553 (14.72)	4546 (14.9)	0.002	4440 (14.55)	4546 (14.9)	0.01
Severe ischemic heart disease	1006 (3.25)	994 (3.26)	0	964 (3.16)	994 (3.26)	0.006
Myocardial infarction	1543 (4.99)	1539 (5.04)	0.001	1465 (4.8)	1539 (5.04)	0.011
Coronary Artery Bypass Graft	170 (0.55)	168 (0.55)	0	181 (0.59)	168 (0.55)	-0.006
Percutaneous Coronary Intervention	418 (1.35)	418 (1.37)	0	396 (1.3)	418 (1.37)	0.006
Peripheral vascular disease	1005 (3.25)	999 (3.27)	0	952 (3.12)	999 (3.27)	0.009
Abdominal aortic aneurysm	512 (1.66)	516 (1.69)	0	508 (1.66)	516 (1.69)	0.002
Heart Failure (CHF)	1584 (5.12)	1573 (5.16)	0	1530 (5.01)	1573 (5.16)	0.006
Severe CHF	288 (0.93)	287 (0.94)	0	276 (0.9)	287 (0.94)	0.004
Defibrillator	88 (0.28)	88 (0.29)	0	83 (0.27)	88 (0.29)	0.003
Atrial fibrillation	1577 (5.1)	1570 (5.15)	0	1522 (4.99)	1570 (5.15)	0.007

© 2022 Keyhani S et al. *JAMA Network Open.*

	Propensity Weighted			Propensity Matched		
	Non-use	Cannabis use	Diff weighted	Non-use	Cannabis use	Diff matched
Cardiac arrhythmia	1610 (5.21)	1601 (5.25)	0	1547 (5.07)	1601 (5.25)	0.008
Chronic Kidney Disease (CKD)	2419 (7.82)	2405 (7.88)	0.001	2407 (7.89)	2405 (7.88)	0
Severe CKD	150 (0.48)	147 (0.48)	0	148 (0.49)	147 (0.48)	0
Dialysis	61 (0.2)	61 (0.2)	0	70 (0.23)	61 (0.2)	-0.006
Asthma	1583 (5.12)	1573 (5.16)	0	1527 (5)	1573 (5.16)	0.007
Bronchiectasis	40 (0.13)	38 (0.12)	0	34 (0.11)	38 (0.12)	0.004
Pulmonary embolism	318 (1.03)	315 (1.03)	0	297 (0.97)	315 (1.03)	0.006
Deep Venous Thrombosis	531 (1.72)	515 (1.69)	0	543 (1.78)	515 (1.69)	-0.007
Cirrhosis	1222 (3.95)	1175 (3.85)	-0.001	1116 (3.66)	1175 (3.85)	0.01
Decompensated cirrhosis	358 (1.16)	342 (1.12)	0	331 (1.08)	342 (1.12)	0.003
Hepatitis	941 (3.04)	867 (2.84)	-0.002	870 (2.85)	867 (2.84)	-0.001
Parkinson's Disease	121 (0.39)	121 (0.4)	0	106 (0.35)	121 (0.4)	0.008
Extrapyramidal disease	795 (2.57)	785 (2.57)	0	769 (2.52)	785 (2.57)	0.003
Multiple Sclerosis	159 (0.51)	161 (0.53)	0	187 (0.61)	161 (0.53)	-0.011
Seizure	636 (2.06)	611 (2)	-0.001	622 (2.04)	611 (2)	-0.003
Falls	950 (3.07)	917 (3.01)	-0.001	884 (2.9)	917 (3.01)	0.006
Orthostatic hypotension	225 (0.73)	220 (0.72)	0	210 (0.69)	220 (0.72)	0.004
Rheumatoid arthritis	600 (1.94)	590 (1.93)	0	575 (1.88)	590 (1.93)	0.004
Other inflammatory conditions	922 (2.98)	922 (3.02)	0	899 (2.95)	922 (3.02)	0.004
AIDS	316 (1.02)	287 (0.94)	-0.001	300 (0.98)	287 (0.94)	-0.004
External lung disease	1262 (4.08)	1199 (3.93)	-0.002	1149 (3.77)	1199 (3.93)	0.009
Bronchitis	404 (1.31)	403 (1.32)	0	369 (1.21)	403 (1.32)	0.01
Lung resection	85 (0.27)	85 (0.28)	0	87 (0.29)	85 (0.28)	-0.001
Influenza	138 (0.45)	128 (0.42)	0	137 (0.45)	128 (0.42)	-0.004
Tracheostomy	83 (0.27)	80 (0.26)	0	88 (0.29)	80 (0.26)	-0.005
Upper respiratory	1858 (6.01)	1849 (6.06)	0.001	1870 (6.13)	1849 (6.06)	-0.003
Ventilation	1380 (4.46)	1364 (4.47)	0	1391 (4.56)	1364 (4.47)	-0.004
Necrotic lung	124 (0.4)	122 (0.4)	0	137 (0.45)	122 (0.4)	-0.008
Respiratory failure	1367 (4.42)	1326 (4.35)	-0.001	1316 (4.31)	1326 (4.35)	0.002
Home oxygen within one year	488 (1.58)	485 (1.59)	0	476 (1.56)	485 (1.59)	0.002
Chronic Obstructive Lung Disease (COPD)	5894 (19.06)	5775 (18.93)	-0.001	5663 (18.56)	5775 (18.93)	0.009
Severe COPD	749 (2.42)	727 (2.38)	0	740 (2.43)	727 (2.38)	-0.003

© 2022 Keyhani S et al. *JAMA Network Open.*

	Propensity Weighted			Propensity Matched		
	**Non-use**	**Cannabis use**	**Diff weighted**	**Non-use**	**Cannabis use**	**Diff matched**
Pulmonary fibrosis	150 (0.48)	143 (0.47)	0	142 (0.47)	143 (0.47)	0
Pneumonia	1325 (4.29)	1290 (4.23)	-0.001	1219 (3.99)	1290 (4.23)	0.012
Sleep apnea	2629 (8.5)	2618 (8.58)	0.001	2576 (8.44)	2618 (8.58)	0.005
Dementia	1391 (4.5)	1383 (4.53)	0	1341 (4.39)	1383 (4.53)	0.007
**Cancer**						
Head and neck	318 (1.03)	310 (1.02)	0	284 (0.93)	310 (1.02)	0.009
Respiratory tract	637 (2.06)	627 (2.05)	0	620 (2.03)	627 (2.05)	0.002
Gastrointestinal tract	364 (1.18)	358 (1.17)	0	362 (1.19)	358 (1.17)	-0.001
Other Gastrointestinal tract	361 (1.17)	333 (1.09)	-0.001	326 (1.07)	333 (1.09)	0.002
Skin	669 (2.16)	671 (2.2)	0	656 (2.15)	671 (2.2)	0.003
Genitourinary	529 (1.71)	517 (1.69)	0	496 (1.63)	517 (1.69)	0.005
Prostate	994 (3.21)	973 (3.19)	0	975 (3.2)	973 (3.19)	0
Central Nervous System	65 (0.21)	64 (0.21)	0	67 (0.22)	64 (0.21)	-0.002
Bone limb connective tissue	139 (0.45)	135 (0.44)	0	138 (0.45)	135 (0.44)	-0.001
Lymphoma/Leukemia	326 (1.06)	319 (1.05)	0	304 (1)	319 (1.05)	0.005
Other Cancer	826 (2.67)	791 (2.59)	-0.001	784 (2.57)	791 (2.59)	0.001
Charlson comorbidity index	2.51 (1.91)	2.5 (1.91)	-0.001	2.47 (2.04)	2.5 (1.91)	0.018
Functional impairment	3989 (12.9)	3958 (12.97)	0.001	3933 (12.89)	3958 (12.97)	0.002
**Body Mass Index (BMI)**						
High BMI	10926 (35.33)	10883 (35.67)	0.003	10931 (35.82)	10883 (35.67)	-0.003
Middle BMI	10765 (34.81)	10630 (34.84)	0	10882 (35.66)	10630 (34.84)	-0.017
Low BMI	8889 (28.74)	8664 (28.39)	-0.004	8365 (27.41)	8664 (28.39)	0.022
Unknown BMI	343 (1.11)	337 (1.1)	0	336 (1.1)	337 (1.1)	0
**Systolic blood pressure**						
Systolic blood pressure < 100	712 (2.3)	699 (2.29)	0	681 (2.23)	699 (2.29)	0.004
Systolic blood pressure 100 - 119.9	6672 (21.58)	6613 (21.67)	0.001	6681 (21.89)	6613 (21.67)	-0.005
Systolic blood pressure 120 - 139.9	14361 (46.44)	14194 (46.52)	0.001	14139 (46.34)	14194 (46.52)	0.004
Systolic blood pressure 140 - 149.9	3905 (12.63)	3832 (12.56)	-0.001	3827 (12.54)	3832 (12.56)	0
Systolic blood pressure 150 - 159.9	2325 (7.52)	2295 (7.52)	0	2284 (7.49)	2295 (7.52)	0.001
Systolic blood pressure 160 - 179.9	1875 (6.06)	1838 (6.02)	0	1832 (6)	1838 (6.02)	0.001
Systolic blood pressure ≥ 180	435 (1.41)	421 (1.38)	0	427 (1.4)	421 (1.38)	-0.002
Systolic blood pressure missing	638 (2.06)	622 (2.04)	0	643 (2.11)	622 (2.04)	-0.005

© 2022 Keyhani S et al. *JAMA Network Open.*

	Propensity Weighted			Propensity Matched		
	Non-use	Cannabis use	Diff weighted	Non-use	Cannabis use	Diff matched
**Albumin**						
Albumin < 2.5	62 (0.2)	63 (0.21)	0	67 (0.22)	63 (0.21)	-0.003
Albumin 2.5 to 3.5	1031 (3.33)	996 (3.26)	-0.001	1004 (3.29)	996 (3.26)	-0.001
Albumin > 3.5	9075 (29.35)	8994 (29.47)	0.001	8954 (29.34)	8994 (29.47)	0.003
Albumin missing	20756 (67.12)	20461 (67.05)	-0.001	20489 (67.15)	20461 (67.05)	-0.002
Low hemoglobin	423 (1.37)	395 (1.29)	-0.001	433 (1.42)	395 (1.29)	-0.011
**Mental Health Conditions**						
Psychosis	1099 (3.55)	1067 (3.5)	-0.001	1032 (3.38)	1067 (3.5)	0.006
Depression	11781 (38.1)	11659 (38.21)	0.001	11543 (37.83)	11659 (38.21)	0.008
Bipolar	1754 (5.67)	1701 (5.57)	-0.001	1727 (5.66)	1701 (5.57)	-0.004
PTSD	7370 (23.83)	7312 (23.96)	0.001	7238 (23.72)	7312 (23.96)	0.006
Anxiety	6314 (20.42)	6252 (20.49)	0.001	6177 (20.24)	6252 (20.49)	0.006
Self-harm	139 (0.45)	131 (0.43)	0	127 (0.42)	131 (0.43)	0.002
**Health Behaviors**						
Opioid use disorder/dependence	1347 (4.36)	1291 (4.23)	-0.001	1271 (4.17)	1291 (4.23)	0.003
Benzodiazepine use/dependence	184 (0.6)	178 (0.58)	0	176 (0.58)	178 (0.58)	0.001
Amphetamine use disorder/dependence	534 (1.73)	488 (1.6)	-0.001	448 (1.47)	488 (1.6)	0.011
Other drug use disorder/dependence	1552 (5.02)	1412 (4.63)	-0.004	1333 (4.37)	1412 (4.63)	0.012
Alcohol use disorder	3664 (11.85)	3467 (11.36)	-0.005	3387 (11.1)	3467 (11.36)	0.008
Elevated audit C score	2188 (7.07)	2073 (6.79)	-0.003	2039 (6.68)	2073 (6.79)	0.004
Current tobacco use	17294 (55.92)	16863 (55.26)	-0.007	16911 (55.42)	16863 (55.26)	-0.003
Positive amphetamine lab	997 (3.23)	935 (3.06)	-0.002	953 (3.12)	935 (3.06)	-0.003
Positive benzodiazepine lab	4291 (13.88)	4201 (13.77)	-0.001	4152 (13.61)	4201 (13.77)	0.005
Positive cocaine lab	807 (2.61)	693 (2.27)	-0.003	648 (2.12)	693 (2.27)	0.01
**Pain Disorders**						
Back and spine disorders	18943 (61.26)	18826 (61.7)	0.004	18762 (61.49)	18826 (61.7)	0.004
Neck and spine disorders	6779 (21.92)	6747 (22.11)	0.002	6700 (21.96)	6747 (22.11)	0.004
Osteoarthritis	8333 (26.95)	8303 (27.21)	0.003	8172 (26.78)	8303 (27.21)	0.01
Neuropathy	4112 (13.3)	4116 (13.49)	0.002	4042 (13.25)	4116 (13.49)	0.007
Headache	5727 (18.52)	5563 (18.23)	-0.003	5576 (18.27)	5563 (18.23)	-0.001
Traumatic brain injury	55 (0.18)	53 (0.17)	0	55 (0.18)	53 (0.17)	-0.002
**Social risk**						

© 2022 Keyhani S et al. *JAMA Network Open.*

	Propensity Weighted			Propensity Matched		
	Non-use	Cannabis use	Diff weighted	Non-use	Cannabis use	Diff matched
Homelessness/marginal housing	2495 (8.07)	2330 (7.64)	-0.004	2315 (7.59)	2330 (7.64)	0.002
Lack of social support	21 (0.07)	20 (0.07)	0	17 (0.06)	20 (0.07)	0.004
**VA Priority group**						
VA Priority group 1	5770 (18.66)	5770 (18.91)	0.003	5843 (19.15)	5770 (18.91)	-0.006
VA Priority group 2	1900 (6.14)	1889 (6.19)	0	1876 (6.15)	1889 (6.19)	0.002
VA Priority group 3	3188 (10.31)	3162 (10.36)	0.001	3212 (10.53)	3162 (10.36)	-0.005
VA Priority group 4	437 (1.41)	432 (1.42)	0	414 (1.36)	432 (1.42)	0.005
VA Priority group 5	11494 (37.17)	11187 (36.66)	-0.005	11116 (36.43)	11187 (36.66)	0.005
VA Priority group 6	659 (2.13)	661 (2.17)	0	654 (2.14)	661 (2.17)	0.002
VA Priority group 7	444 (1.44)	436 (1.43)	0	462 (1.51)	436 (1.43)	-0.007
VA Priority group 8	2316 (7.49)	2275 (7.46)	0	2303 (7.55)	2275 (7.46)	-0.003
VA Priority unknown	4715 (15.25)	4702 (15.41)	0.002	4634 (15.19)	4702 (15.41)	0.006
**Utilization in past year**						
ICU visits within six months						
ICU visits within six months (none)	30361 (98.18)	29972 (98.22)	0	29989 (98.28)	29972 (98.22)	-0.004
ICU visits within six months (one)	492 (1.59)	471 (1.54)	0	464 (1.52)	471 (1.54)	0.002
ICU visits within six months (two or more)	70 (0.23)	71 (0.23)	0	61 (0.2)	71 (0.23)	0.007
ICU visits within one year						
ICU visits within one year (none)	29997 (97)	29618 (97.06)	0.001	29653 (97.18)	29618 (97.06)	-0.007
ICU visits within one year (one)	750 (2.43)	728 (2.39)	0	699 (2.29)	728 (2.39)	0.006
ICU visits within one year (two or more)	176 (0.57)	168 (0.55)	0	162 (0.53)	168 (0.55)	0.003
**Hospitalized within one year**	4176 (13.5)	4084 (13.38)	-0.001	4050 (13.27)	4084 (13.38)	0.003
ED visits within six months						
ED visits within six months (none)	21778 (70.43)	21606 (70.81)	0.004	21613 (70.83)	21606 (70.81)	-0.001
ED visits within six months (one)	5031 (16.27)	4930 (16.16)	-0.001	4901 (16.06)	4930 (16.16)	0.003
ED visits within six months (two or more)	4114 (13.3)	3978 (13.04)	-0.003	4000 (13.11)	3978 (13.04)	-0.002
ED visits within one year						
ED visits within one year (none)	18536 (59.94)	18395 (60.28)	0.003	18391 (60.27)	18395 (60.28)	0
ED visits within one year (one)	5732 (18.54)	5627 (18.44)	-0.001	5611 (18.39)	5627 (18.44)	0.001
ED visits within one year (two or more)	6655 (21.52)	6492 (21.28)	-0.002	6512 (21.34)	6492 (21.28)	-0.002
VA visit count	31.3 (23.05)	31.3 (23.35)	0	31.23 (23.33)	31.3 (23.35)	0.003
**Psychoactive Drugs in past year**						

© 2022 Keyhani S et al. *JAMA Network Open.*

	Propensity Weighted			Propensity Matched		
	Non-use	Cannabis use	Diff weighted	Non-use	Cannabis use	Diff matched
Morphine Equivalents over prior 90-day period	191.96 (451.68)	194.22 (456.72)	0.005	191.98 (469.17)	194.22 (456.72)	0.005
Long-acting opioid	6424 (20.77)	6476 (21.22)	0.004	6354 (20.82)	6476 (21.22)	0.01
Alcohol use disorder treatment medication	53 (0.17)	53 (0.17)	0	48 (0.16)	53 (0.17)	0.004
Benzodiazepines	6675 (21.59)	6604 (21.64)	0.001	6583 (21.57)	6604 (21.64)	0.002
Gaba drugs	7202 (23.29)	7188 (23.56)	0.003	7134 (23.38)	7188 (23.56)	0.004
Muscle relaxants	6735 (21.78)	6668 (21.85)	0.001	6687 (21.91)	6668 (21.85)	-0.002
Antidepressants	9934 (32.13)	9913 (32.49)	0.004	9906 (32.46)	9913 (32.49)	0
Antipsychotics	2753 (8.9)	2691 (8.82)	-0.001	2705 (8.86)	2691 (8.82)	-0.002
Sedatives	62 (0.2)	61 (0.2)	0	52 (0.17)	61 (0.2)	0.007
**Number of psychoactive drugs in past year**						
1	11375 (36.78)	11145 (36.52)	-0.003	11179 (36.64)	11145 (36.52)	-0.002
2	9926 (32.1)	9796 (32.1)	0	9720 (31.85)	9796 (32.1)	0.005
3	6266 (20.26)	6230 (20.42)	0.002	6314 (20.69)	6230 (20.42)	-0.007
4	2595 (8.39)	2580 (8.46)	0.001	2548 (8.35)	2580 (8.46)	0.004
5 or more	762 (2.46)	763 (2.5)	0	753 (2.47)	763 (2.5)	0.002
**Care Assessments Need (CAN) score**						
CAN Score < 10	2021 (6.54)	2019 (6.62)	0.001	2055 (6.73)	2019 (6.62)	-0.005
CAN Score 10 - 19.9	2737 (8.85)	2733 (8.96)	0.001	2856 (9.36)	2733 (8.96)	-0.014
CAN Score 20 - 29.9	3063 (9.91)	3015 (9.88)	0	3038 (9.96)	3015 (9.88)	-0.003
CAN Score 30 - 39.9	3753 (12.14)	3700 (12.13)	0	3802 (12.46)	3700 (12.13)	-0.01
CAN Score 40 - 49.9	3859 (12.48)	3819 (12.52)	0	3860 (12.65)	3819 (12.52)	-0.004
CAN Score 50 - 59.9	3824 (12.36)	3774 (12.37)	0	3712 (12.16)	3774 (12.37)	0.006
CAN Score 60 - 69.9	3425 (11.07)	3372 (11.05)	0	3305 (10.83)	3372 (11.05)	0.007
CAN Score 70 - 79.9	3269 (10.57)	3231 (10.59)	0	3151 (10.33)	3231 (10.59)	0.009
CAN Score 80 - 89.9	2516 (8.14)	2472 (8.1)	0	2368 (7.76)	2472 (8.1)	0.013
CAN Score 90 – 98.9	1572 (5.08)	1522 (4.99)	-0.001	1477 (4.84)	1522 (4.99)	0.007
CAN Score Missing	885 (2.86)	857 (2.81)	-0.001	890 (2.92)	857 (2.81)	-0.006
**Year Index Urine Drug Screen was ordered**						
2014	16444 (53.18)	16400 (53.75)	0.006	16299 (53.41)	16400 (53.75)	0.007
2015	7633 (24.68)	7518 (24.64)	0	7506 (24.6)	7518 (24.64)	0.001

© 2022 Keyhani S et al. *JAMA Network Open.*

	Propensity Weighted			Propensity Matched		
	Non-use	Cannabis use	Diff weighted	Non-use	Cannabis use	Diff matched
2016	3521 (11.39)	3451 (11.31)	-0.001	3457 (11.33)	3451 (11.31)	-0.001
2017	1698 (5.49)	1633 (5.35)	-0.001	1696 (5.56)	1633 (5.35)	-0.009
2018	1000 (3.24)	928 (3.04)	-0.002	958 (3.14)	928 (3.04)	-0.006
2019	626 (2.03)	584 (1.91)	-0.001	598 (1.96)	584 (1.91)	-0.003

* Facility level variable not shown

© 2022 Keyhani S et al. *JAMA Network Open.*

eTable 4. Propensity-Weighted and Matched Adults Receiving Long-Term (>84 Days) Prescription Opioid Therapy in Past 90 Days (Full List Of Variables)*

	Propensity Weighted			Propensity Matched		
	**Non-use**	**Cannabis use**	**Diff weighted**	**Non-use**	**Cannabis use**	**Diff matched**
N	18583.95	18401		18401	18401	
Age	58.39 (9.49)	58.43 (9.53)	0.004	58.34 (9.58)	58.43 (9.53)	0.009
Male	17626 (94.84)	17449 (94.83)	0	17453 (94.85)	17449 (94.83)	-0.001
Female	958 (5.16)	952 (5.17)	0	948 (5.15)	952 (5.17)	0.001
Married	7139 (38.41)	7173 (38.98)	0.006	7130 (38.75)	7173 (38.98)	0.005
**Race**						
White	14405 (77.51)	14326 (77.85)	0.003	14278 (77.59)	14326 (77.85)	0.006
Black or African American	2493 (13.41)	2406 (13.08)	-0.003	2417 (13.14)	2406 (13.08)	-0.002
Asian	61 (0.33)	59 (0.32)	0	70 (0.38)	59 (0.32)	-0.01
Native Hawaiian or other Pacific Islander	165 (0.89)	161 (0.87)	0	173 (0.94)	161 (0.87)	-0.007
American Indian or Alaska Native	304 (1.63)	298 (1.62)	0	332 (1.8)	298 (1.62)	-0.014
Race unknown	1157 (6.23)	1151 (6.26)	0	1131 (6.15)	1151 (6.26)	0.005
**Hispanic**	739 (3.98)	722 (3.92)	-0.001	759 (4.12)	722 (3.92)	-0.01
**Clinical Conditions**						
Hypertension	10913 (58.72)	10785 (58.61)	-0.001	10805 (58.72)	10785 (58.61)	-0.002
Hyperlipidemia	9017 (48.52)	8985 (48.83)	0.003	9012 (48.98)	8985 (48.83)	-0.003
Diabetes	4346 (23.39)	4325 (23.5)	0.001	4347 (23.62)	4325 (23.5)	-0.003
Stroke	364 (1.96)	351 (1.91)	0	353 (1.92)	351 (1.91)	-0.001
Transient Ischemic Attack	183 (0.99)	182 (0.99)	0	182 (0.99)	182 (0.99)	0
Paralytic syndromes	501 (2.7)	492 (2.67)	0	489 (2.66)	492 (2.67)	0.001
Ischemic heart disease	2807 (15.11)	2792 (15.17)	0.001	2808 (15.26)	2792 (15.17)	-0.002
Severe ischemic heart disease	519 (2.79)	512 (2.78)	0	494 (2.68)	512 (2.78)	0.006
Myocardial infarction	930 (5.01)	917 (4.98)	0	937 (5.09)	917 (4.98)	-0.005
Coronary Artery Bypass Graft	91 (0.49)	88 (0.48)	0	85 (0.46)	88 (0.48)	0.002
Percutaneous Coronary Intervention	245 (1.32)	244 (1.33)	0	240 (1.3)	244 (1.33)	0.002
Peripheral vascular disease	512 (2.76)	504 (2.74)	0	529 (2.87)	504 (2.74)	-0.008
Abdominal aortic aneurysm	285 (1.54)	290 (1.58)	0	294 (1.6)	290 (1.58)	-0.002
Heart Failure (CHF)	946 (5.09)	938 (5.1)	0	983 (5.34)	938 (5.1)	-0.011
Severe CHF	137 (0.74)	136 (0.74)	0	145 (0.79)	136 (0.74)	-0.006
Atrial fibrillation	915 (4.92)	913 (4.96)	0	951 (5.17)	913 (4.96)	-0.009

© 2022 Keyhani S et al. *JAMA Network Open.*

	Propensity Weighted			Propensity Matched		
	Non-use	Cannabis use	Diff weighted	Non-use	Cannabis use	Diff matched
Cardiac arrhythmia	951 (5.12)	945 (5.14)	0	965 (5.24)	945 (5.14)	-0.005
Chronic Kidney Disease (CKD)	1468 (7.9)	1456 (7.91)	0	1425 (7.74)	1456 (7.91)	0.006
Severe CKD	81 (0.44)	79 (0.43)	0	89 (0.48)	79 (0.43)	-0.008
Dialysis	30 (0.16)	29 (0.16)	0	25 (0.14)	29 (0.16)	0.006
Asthma	982 (5.28)	982 (5.34)	0.001	966 (5.25)	982 (5.34)	0.004
Bronchiectasis	17 (0.09)	17 (0.09)	0	18 (0.1)	17 (0.09)	-0.002
Pulmonary embolism	191 (1.03)	186 (1.01)	0	185 (1.01)	186 (1.01)	0.001
Deep Venous Thrombosis	308 (1.66)	299 (1.62)	0	295 (1.6)	299 (1.62)	0.002
Cirrhosis	763 (4.11)	738 (4.01)	-0.001	733 (3.98)	738 (4.01)	0.001
Decompensated cirrhosis	224 (1.21)	211 (1.15)	-0.001	229 (1.24)	211 (1.15)	-0.009
Hepatitis	392 (2.11)	368 (2)	-0.001	377 (2.05)	368 (2)	-0.003
Parkinson's Disease	66 (0.35)	66 (0.36)	0	62 (0.34)	66 (0.36)	0.004
Extrapyramidal disease	517 (2.78)	511 (2.78)	0	510 (2.77)	511 (2.78)	0
Multiple Sclerosis	96 (0.52)	99 (0.54)	0	112 (0.61)	99 (0.54)	-0.009
Seizure	390 (2.1)	381 (2.07)	0	346 (1.88)	381 (2.07)	0.014
Falls	542 (2.92)	529 (2.87)	0	559 (3.04)	529 (2.87)	-0.01
Orthostatic hypotension	127 (0.68)	126 (0.68)	0	133 (0.72)	126 (0.68)	-0.005
Rheumatoid arthritis	383 (2.06)	370 (2.01)	0	416 (2.26)	370 (2.01)	-0.017
Other inflammatory conditions	573 (3.08)	576 (3.13)	0	611 (3.32)	576 (3.13)	-0.011
AIDS	197 (1.06)	179 (0.97)	-0.001	160 (0.87)	179 (0.97)	0.011
Defibrillator	50 (0.27)	50 (0.27)	0	48 (0.26)	50 (0.27)	0.002
External lung disease	687 (3.69)	658 (3.58)	-0.001	661 (3.59)	658 (3.58)	-0.001
Bronchitis	230 (1.24)	232 (1.26)	0	212 (1.15)	232 (1.26)	0.01
Lung resection	46 (0.25)	48 (0.26)	0	52 (0.28)	48 (0.26)	-0.004
Influenza	92 (0.5)	83 (0.45)	0	91 (0.49)	83 (0.45)	-0.006
Tracheostomy	41 (0.22)	40 (0.22)	0	34 (0.18)	40 (0.22)	0.007
Upper respiratory	1128 (6.07)	1118 (6.08)	0	1083 (5.89)	1118 (6.08)	0.008
Ventilation	784 (4.22)	774 (4.21)	0	774 (4.21)	774 (4.21)	0
Necrotic lung	79 (0.42)	75 (0.41)	0	68 (0.37)	75 (0.41)	0.006
Respiratory failure	833 (4.48)	811 (4.41)	-0.001	817 (4.44)	811 (4.41)	-0.002
Home oxygen within one year	321 (1.73)	318 (1.73)	0	316 (1.72)	318 (1.73)	0.001
Chronic Obstructive Lung Disease (COPD)	3834 (20.63)	3762 (20.44)	-0.002	3770 (20.49)	3762 (20.44)	-0.001
Severe COPD	421 (2.26)	408 (2.22)	0	422 (2.29)	408 (2.22)	-0.005

© 2022 Keyhani S et al. *JAMA Network Open.*

	Propensity Weighted			Propensity Matched		
	**Non-use**	**Cannabis use**	**Diff weighted**	**Non-use**	**Cannabis use**	**Diff matched**
Pulmonary fibrosis	81 (0.43)	79 (0.43)	0	78 (0.42)	79 (0.43)	0.001
Pneumonia	839 (4.51)	816 (4.43)	-0.001	825 (4.48)	816 (4.43)	-0.002
Sleep apnea	1555 (8.37)	1551 (8.43)	0.001	1566 (8.51)	1551 (8.43)	-0.003
Dementia	880 (4.74)	878 (4.77)	0	874 (4.75)	878 (4.77)	0.001
**Cancer**						
Head and neck	191 (1.03)	187 (1.02)	0	187 (1.02)	187 (1.02)	0
Lung respiratory tract	376 (2.02)	375 (2.04)	0	369 (2.01)	375 (2.04)	0.002
Gastrointestinal tract	217 (1.17)	209 (1.14)	0	221 (1.2)	209 (1.14)	-0.006
Other Gastrointestinal tract	194 (1.04)	179 (0.97)	-0.001	185 (1.01)	179 (0.97)	-0.003
Skin	405 (2.18)	410 (2.23)	0.001	378 (2.05)	410 (2.23)	0.012
Genitourinary	313 (1.69)	309 (1.68)	0	288 (1.57)	309 (1.68)	0.009
Prostate	566 (3.05)	553 (3.01)	0	586 (3.18)	553 (3.01)	-0.01
Central Nervous System	36 (0.2)	37 (0.2)	0	26 (0.14)	37 (0.2)	0.014
Bone limb connective tissue	78 (0.42)	79 (0.43)	0	71 (0.39)	79 (0.43)	0.007
Lymphoma/Leukemia	194 (1.04)	189 (1.03)	0	170 (0.92)	189 (1.03)	0.011
Other Cancer	484 (2.61)	461 (2.51)	-0.001	459 (2.49)	461 (2.51)	0.001
Charlson comorbidity index	2.56 (1.86)	2.55 (1.85)	-0.004	2.56 (1.85)	2.55 (1.85)	-0.002
Functional impairment	2403 (12.93)	2393 (13)	0.001	2415 (13.12)	2393 (13)	-0.004
**Body Mass Index (BMI)**						
High BMI	6610 (35.57)	6593 (35.83)	0.003	6700 (36.41)	6593 (35.83)	-0.012
Middle BMI	6465 (34.79)	6389 (34.72)	-0.001	6417 (34.87)	6389 (34.72)	-0.003
Low BMI	5366 (28.88)	5274 (28.66)	-0.002	5124 (27.85)	5274 (28.66)	0.018
Unknown BMI	143 (0.77)	145 (0.79)	0	160 (0.87)	145 (0.79)	-0.009
**Systolic blood pressure**						
Systolic blood pressure < 100	455 (2.45)	447 (2.43)	0	458 (2.49)	447 (2.43)	-0.004
Systolic blood pressure 100 - 119.9	4010 (21.58)	3974 (21.6)	0	4017 (21.83)	3974 (21.6)	-0.006
Systolic blood pressure 120 - 139.9	8733 (46.99)	8668 (47.11)	0.001	8547 (46.45)	8668 (47.11)	0.013
Systolic blood pressure 140 - 149.9	2288 (12.31)	2264 (12.3)	0	2226 (12.1)	2264 (12.3)	0.006
Systolic blood pressure 150 - 159.9	1338 (7.2)	1332 (7.24)	0	1391 (7.56)	1332 (7.24)	-0.012
Systolic blood pressure 160 - 179.9	1125 (6.05)	1101 (5.98)	-0.001	1112 (6.04)	1101 (5.98)	-0.003
Systolic blood pressure ≥ 180	238 (1.28)	229 (1.24)	0	255 (1.39)	229 (1.24)	-0.012
Systolic blood pressure Missing	397 (2.13)	386 (2.1)	0	395 (2.15)	386 (2.1)	-0.003
**Albumin**						

© 2022 Keyhani S et al. *JAMA Network Open.*

	Propensity Weighted			Propensity Matched		
	**Non-use**	**Cannabis use**	**Diff weighted**	**Non-use**	**Cannabis use**	**Diff matched**
Albumin < 2.5	27 (0.14)	28 (0.15)	0	25 (0.14)	28 (0.15)	0.004
Albumin 2.5 to 3.5	555 (2.99)	537 (2.92)	-0.001	549 (2.98)	537 (2.92)	-0.004
Albumin > 3.5	5501 (29.6)	5454 (29.64)	0	5303 (28.82)	5454 (29.64)	0.018
Albumin missing	12502 (67.27)	12382 (67.29)	0	12524 (68.06)	12382 (67.29)	-0.016
Low hemoglobin	224 (1.21)	215 (1.17)	0	214 (1.16)	215 (1.17)	0.001
**Mental Health Conditions**						
Psychosis	668 (3.6)	655 (3.56)	0	636 (3.46)	655 (3.56)	0.006
Depression	7496 (40.33)	7437 (40.42)	0.001	7383 (40.12)	7437 (40.42)	0.006
Bipolar	1100 (5.92)	1071 (5.82)	-0.001	1071 (5.82)	1071 (5.82)	0
PTSD	4500 (24.21)	4483 (24.36)	0.001	4482 (24.36)	4483 (24.36)	0
Anxiety	3973 (21.38)	3935 (21.38)	0	3933 (21.37)	3935 (21.38)	0
Self-harm	85 (0.46)	82 (0.45)	0	87 (0.47)	82 (0.45)	-0.004
**Health Behaviors**						
Opioid use disorder/dependence	900 (4.84)	872 (4.74)	-0.001	885 (4.81)	872 (4.74)	-0.003
Benzodiazepine use disorder/dependence	103 (0.55)	102 (0.55)	0	113 (0.61)	102 (0.55)	-0.008
Amphetamine use disorder/dependence	304 (1.64)	283 (1.54)	-0.001	274 (1.49)	283 (1.54)	0.004
Other drug use disorder/dependence	921 (4.95)	853 (4.64)	-0.003	860 (4.67)	853 (4.64)	-0.002
Alcohol use disorder	2092 (11.26)	1983 (10.78)	-0.005	1944 (10.56)	1983 (10.78)	0.007
Elevated audit C score	1082 (5.82)	1040 (5.65)	-0.002	1012 (5.5)	1040 (5.65)	0.007
Current tobacco use	10717 (57.67)	10485 (56.98)	-0.007	10426 (56.66)	10485 (56.98)	0.006
Positive amphetamine lab	564 (3.03)	543 (2.95)	-0.001	523 (2.84)	543 (2.95)	0.006
Positive benzodiazepine lab	2787 (15)	2737 (14.87)	-0.001	2787 (15.15)	2737 (14.87)	-0.008
Positive cocaine lab	369 (1.98)	334 (1.82)	-0.002	312 (1.7)	334 (1.82)	0.009
**Pain Disorders**						
Back and spine disorders	12170 (65.49)	12108 (65.8)	0.003	12033 (65.39)	12108 (65.8)	0.009
Neck and spine disorders	4354 (23.43)	4346 (23.62)	0.002	4317 (23.46)	4346 (23.62)	0.004
Osteoarthritis	5309 (28.57)	5282 (28.7)	0.001	5233 (28.44)	5282 (28.7)	0.006
Neuropathy	2581 (13.89)	2587 (14.06)	0.002	2571 (13.97)	2587 (14.06)	0.003
Headache	3396 (18.28)	3342 (18.16)	-0.001	3330 (18.1)	3342 (18.16)	0.002
Traumatic brain injury	34 (0.19)	33 (0.18)	0	53 (0.29)	33 (0.18)	-0.023
**Social Risk**						
Homelessness	1376 (7.4)	1320 (7.17)	-0.002	1333 (7.24)	1320 (7.17)	-0.003
Lack of social support	12 (0.06)	10 (0.05)	0	9 (0.05)	10 (0.05)	0.002

© 2022 Keyhani S et al. *JAMA Network Open.*

	Propensity Weighted			Propensity Matched		
	Non-use	Cannabis use	Diff weighted	Non-use	Cannabis use	Diff matched
**VA Priority group**						
VA Priority group 1	3439 (18.51)	3459 (18.8)	0.003	3448 (18.74)	3459 (18.8)	0.002
VA Priority group 2	1102 (5.93)	1103 (5.99)	0.001	1120 (6.09)	1103 (5.99)	-0.004
VA Priority group 3	1917 (10.32)	1896 (10.3)	0	1899 (10.32)	1896 (10.3)	-0.001
VA Priority group 4	299 (1.61)	295 (1.6)	0	286 (1.55)	295 (1.6)	0.004
VA Priority group 5	7078 (38.08)	6926 (37.64)	-0.004	6972 (37.89)	6926 (37.64)	-0.005
VA Priority group 6	315 (1.69)	312 (1.7)	0	305 (1.66)	312 (1.7)	0.003
VA Priority group 7	229 (1.23)	227 (1.23)	0	234 (1.27)	227 (1.23)	-0.003
VA Priority group 8	1350 (7.26)	1333 (7.24)	0	1273 (6.92)	1333 (7.24)	0.013
VA Priority unknown	2855 (15.36)	2850 (15.49)	0.001	2864 (15.56)	2850 (15.49)	-0.002
**Utilization in past year**						
ICU visits within six months						
ICU visits within six months (none)	18273 (98.32)	18098 (98.35)	0	18096 (98.34)	18098 (98.35)	0.001
ICU visits within six months (one)	264 (1.42)	258 (1.4)	0	263 (1.43)	258 (1.4)	-0.002
ICU visits within six months (two or more)	47 (0.26)	45 (0.24)	0	42 (0.23)	45 (0.24)	0.003
ICU visits within one year						
ICU visits within one year (none)	18048 (97.12)	17885 (97.2)	0.001	17885 (97.2)	17885 (97.2)	0
ICU visits within one year (one)	426 (2.29)	415 (2.26)	0	419 (2.28)	415 (2.26)	-0.001
ICU visits within one year (two or more)	109 (0.59)	101 (0.55)	0	97 (0.53)	101 (0.55)	0.003
**Hospitalized within one year**	2282 (12.28)	2243 (12.19)	-0.001	2274 (12.36)	2243 (12.19)	-0.005
ED visits within six months						
ED visits within six months (none)	14137 (76.07)	14015 (76.16)	0.001	13909 (75.59)	14015 (76.16)	0.013
ED visits within six months (one)	2666 (14.34)	2623 (14.25)	-0.001	2639 (14.34)	2623 (14.25)	-0.002
ED visits within six months (two or more)	1781 (9.58)	1763 (9.58)	0	1853 (10.07)	1763 (9.58)	-0.016
ED visits within one year						
ED visits within one year (none)	11987 (64.5)	11891 (64.62)	0.001	11800 (64.13)	11891 (64.62)	0.01
ED visits within one year (one)	3245 (17.46)	3199 (17.38)	-0.001	3176 (17.26)	3199 (17.38)	0.003
ED visits within one year (two or more)	3352 (18.04)	3311 (17.99)	0	3425 (18.61)	3311 (17.99)	-0.016
VA visit count	32.57 (22.66)	32.57 (23.27)	0	32.55 (22.56)	32.57 (23.27)	0.001
**Psychoactive Drugs in past year**						
Morphine Equivalents over prior 90-day period	275.09 (556.77)	277.67 (561.34)	0.005	275.99 (527.01)	277.67 (561.34)	0.003
Long-acting opioid	5493 (29.56)	5534 (30.07)	0.005	5469 (29.72)	5534 (30.07)	0.008

© 2022 Keyhani S et al. *JAMA Network Open.*

	Propensity Weighted			Propensity Matched		
	Non-use	Cannabis use	Diff weighted	Non-use	Cannabis use	Diff matched
Alcohol use disorder treatment medication	31 (0.17)	30 (0.16)	0	26 (0.14)	30 (0.16)	0.006
Benzodiazepines	4588 (24.69)	4549 (24.72)	0	4557 (24.76)	4549 (24.72)	-0.001
Gaba drugs	4484 (24.13)	4496 (24.43)	0.003	4480 (24.35)	4496 (24.43)	0.002
Muscle relaxants	3987 (21.45)	3983 (21.65)	0.002	3945 (21.44)	3983 (21.65)	0.005
Antidepressants	6292 (33.86)	6310 (34.29)	0.004	6209 (33.74)	6310 (34.29)	0.012
Antipsychotics	1792 (9.64)	1769 (9.61)	0	1786 (9.71)	1769 (9.61)	-0.003
Sedatives	27 (0.15)	27 (0.15)	0	20 (0.11)	27 (0.15)	0.011
**Number of psychoactive drugs in past year**						
1	6386 (34.36)	6262 (34.03)	-0.003	6343 (34.47)	6262 (34.03)	-0.009
2	6000 (32.29)	5942 (32.29)	0	5924 (32.19)	5942 (32.29)	0.002
3	3980 (21.42)	3968 (21.56)	0.001	3920 (21.3)	3968 (21.56)	0.006
4	1711 (9.21)	1715 (9.32)	0.001	1678 (9.12)	1715 (9.32)	0.007
5	506 (2.72)	514 (2.79)	0.001	536 (2.91)	514 (2.79)	-0.007
**Care Assessment Need (CAN) score**						
CAN score < 10	1067 (5.74)	1061 (5.77)	0	1062 (5.77)	1061 (5.77)	0
CAN score 10 - 19.9	1530 (8.23)	1527 (8.3)	0.001	1511 (8.21)	1527 (8.3)	0.003
CAN score 20 - 29.9	1784 (9.6)	1754 (9.53)	-0.001	1784 (9.7)	1754 (9.53)	-0.006
CAN score 30 - 39.9	2257 (12.15)	2249 (12.22)	0.001	2271 (12.34)	2249 (12.22)	-0.004
CAN score 40 - 49.9	2432 (13.09)	2410 (13.1)	0	2448 (13.3)	2410 (13.1)	-0.006
CAN score 50 - 59.9	2424 (13.04)	2417 (13.14)	0.001	2419 (13.15)	2417 (13.14)	0
CAN score 60 - 69.9	2207 (11.88)	2173 (11.81)	-0.001	2200 (11.96)	2173 (11.81)	-0.005
CAN score 70 - 79.9	2111 (11.36)	2097 (11.4)	0	2034 (11.05)	2097 (11.4)	0.011
CAN score 80 - 89.9	1575 (8.47)	1556 (8.46)	0	1528 (8.3)	1556 (8.46)	0.005
CAN score 90 - 98.9	918 (4.94)	886 (4.81)	-0.001	877 (4.77)	886 (4.81)	0.002
CAN score missing	280 (1.51)	271 (1.47)	0	267 (1.45)	271 (1.47)	0.002
**Year Index Urine Drug Screen was ordered**						
2014	11290 (60.75)	11264 (61.21)	0.005	11184 (60.78)	11264 (61.21)	0.009
2015	4589 (24.69)	4521 (24.57)	-0.001	4579 (24.88)	4521 (24.57)	-0.007
2016	1667 (8.97)	1637 (8.9)	-0.001	1634 (8.88)	1637 (8.9)	0.001
2017	598 (3.22)	563 (3.06)	-0.002	597 (3.24)	563 (3.06)	-0.011
2018	309 (1.66)	295 (1.6)	-0.001	269 (1.46)	295 (1.6)	0.012
2019	131 (0.7)	121 (0.66)	0	138 (0.75)	121 (0.66)	-0.011

© 2022 Keyhani S et al. *JAMA Network Open.*

* Facility level variable not shown

© 2022 Keyhani S et al. *JAMA Network Open.*

eFigure 1. Survival Probability Among Adults Followed up for 180 Days Receiving Prescription Opioid Therapy in Prior 90 Days by Cannabis Use

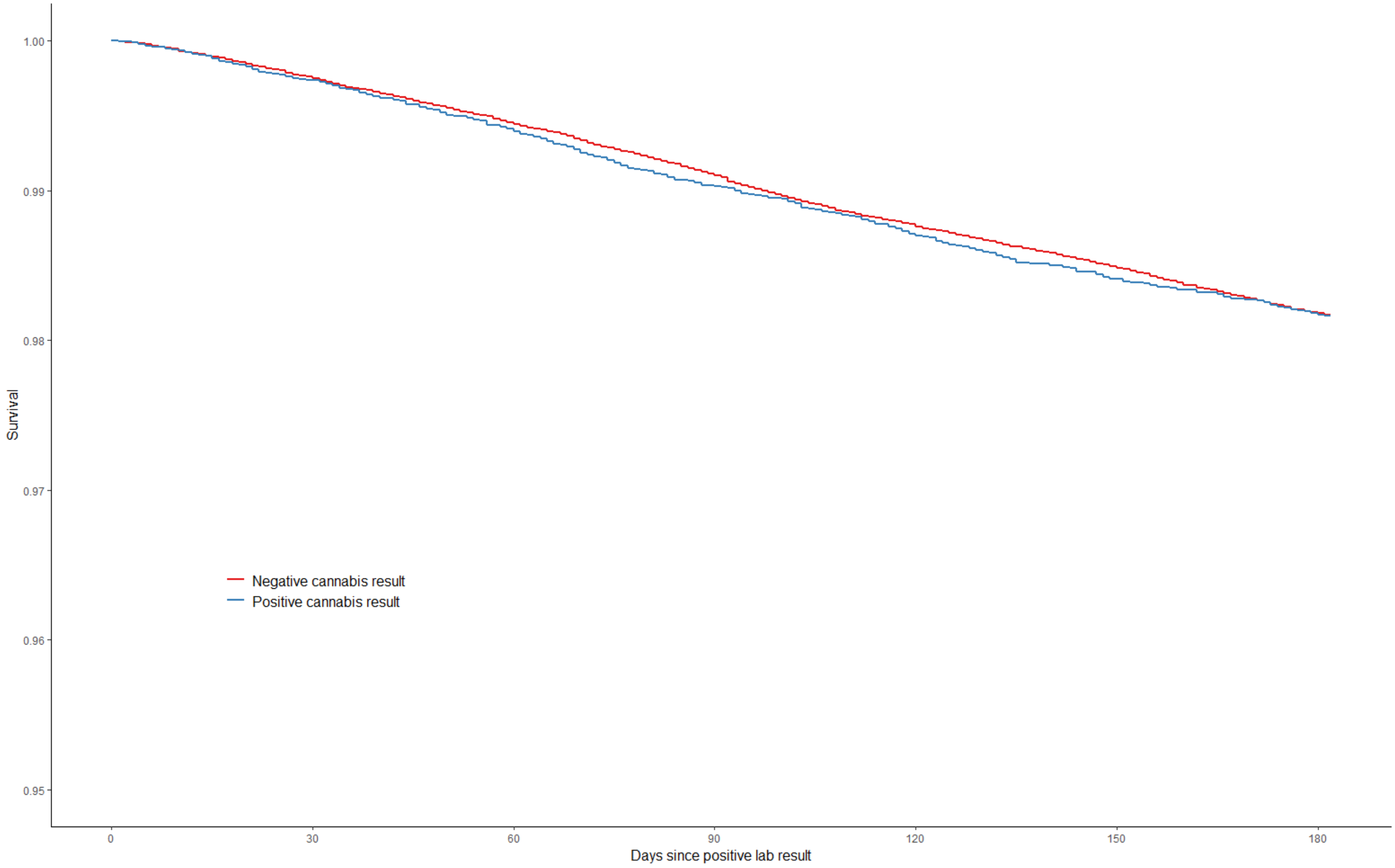

© 2022 Keyhani S et al. *JAMA Network Open.*

eFigure 2. Probability of Emergency Department Visits, Hospitalization, or Mortality Among Adults Followed up for 180 Days Receiving Any Prescription Opioid Therapy by Cannabis Use

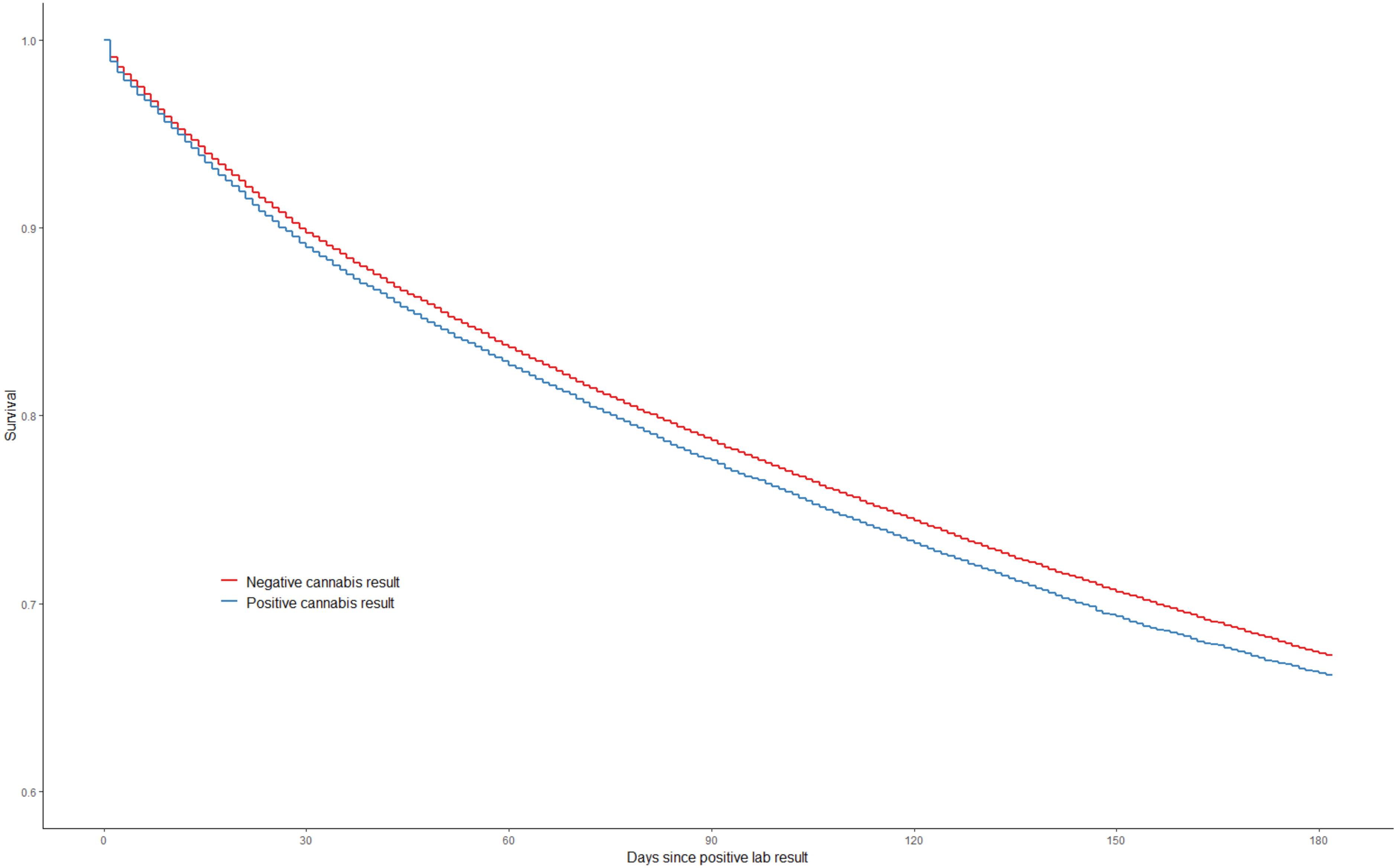

© 2022 Keyhani S et al. *JAMA Network Open.*

eTable 5. Association of Cannabis Use With 90- and 180-Day Adverse Events Among Adults Receiving Prescription Opioids (Propensity Matching Approach)

	**Unadjusted events by arm**	**90-day mortality**	**Unadjusted events by arm**	**180-day mortality**
	**N (%)**	**HR, 95%CI**	**N (%)**	**HR, 95%CI**
All adults on opioids in the past 90 days	Total = 564<br>Opioids only = 265 (0.87%)<br>Opioid and cannabis = 299 (0.98%)	Hazard ratio = 1.13 (0.95, 1.33)<br>p = 0.15	Total = 1055<br>Opioids only = 495 (1.62%)<br>Opioid and cannabis = 560 (1.84%)	**Hazard ratio = 1.13 (1.01, 1.27)<br>p = 0.04**
All adults on LTOT (opioids on >84 days out of the past 90 days)	Total = 284<br>Opioids only = 134 (0.73%)<br>Opioid and cannabis = 150 (0.82%)	Hazard ratio = 1.12 (0.88, 1.41)<br>p = 0.34	Total = 581<br>Opioids only = 291 (1.58%)<br>Opioid and cannabis = 290 (1.58%)	Hazard ratio = 1 (0.84, 1.17)<br>p = 0.97
	**Unadjusted events by arm**	**90-day ED visits, hospitalization, or mortality**	**Unadjusted events by arm**	**180-day ED visits, hospitalization, or mortality**
	**N (%)**	**HR, 95%CI**	**N (%)**	**HR, 95%CI**
All adults on opioids in the last 90 days	Total = 13017<br>Opioids only = 6350 (21.52%)<br>Opioid and cannabis = 6667 (22.59%)	**Hazard ratio = 1.06 (1.023, 1.095)<br>p = 0.001**	Total = 19600<br>Opioids only = 9622 (32.61%)<br>Opioid and cannabis = 9978 (33.81%)	**Hazard ratio = 1.05 (1.02, 1.078)<br>p = 0.001**
All adults on LTOT (opioids on >84 days out of the past 90 days)	Total = 1935<br>Opioids only = 929 (19.35%)<br>Opioid and cannabis = 1006 (20.97%)	**Hazard ratio = 1.09 (1.01, 1.19)<br>p = 0.05**	Total = 3024<br>Opioids only = 1465 (30.51%)<br>Opioid and cannabis = 1559 (32.5%)	**Hazard ratio = 1.08 (1.01, 1.16)<br>p = 0.03**

© 2022 Keyhani S et al. *JAMA Network Open.*

eTable 6. Baseline Characteristics of 135 413 Adults Aged 65 Years and Older Receiving Any Prescription Opioid in 90 Days Before Urine Drug Screening by Cannabis Use (All Baseline Variables)*

	**Non-Use**	**Cannabis Use**	**p-value**
N	127044	8369	
Age (SE)	71.95 (6.68)	68.33 (3.72)	<0.001
Male	123957 (97.6)	8274 (98.9)	<0.001
Female	3087 (2.4)	95 (1.1)	<0.001
Married	74823 (58.9)	3598 (43.0)	<0.001
**Race**			
White	105899 (83.4)	6490 (77.5)	<0.001
Black or African American	10933 (8.6)	1085 (13.0)	<0.001
Asian	271 (0.2)	21 (0.3)	0.551
Native Hawaiian/Pacific Islander	1052 (0.8)	78 (0.9)	0.342
American Indian or Alaska Native	1219 (1.0)	120 (1.4)	<0.001
Race unknown	7670 (6.0)	575 (6.9)	0.002
**Hispanic**	3567 (2.8)	316 (3.8)	<0.001
**Clinical Conditions**			
Hypertension	104026 (81.9)	5970 (71.3)	<0.001
Hyperlipidemia	90847 (71.5)	4847 (57.9)	<0.001
Diabetes	55235 (43.5)	2480 (29.6)	<0.001
Stroke	4702 (3.7)	240 (2.9)	<0.001
Transient Ischemic Attack	3409 (2.7)	134 (1.6)	<0.001
Paralytic syndromes	3868 (3.0)	216 (2.6)	0.018
Ischemic heart disease	46375 (36.5)	1986 (23.7)	<0.001
Severe ischemic heart disease	9482 (7.5)	443 (5.3)	<0.001
Myocardial infarction	13063 (10.3)	644 (7.7)	<0.001
Coronary Artery Bypass Graft	1095 (0.9)	63 (0.8)	0.323
Percutaneous Coronary Intervention	3400 (2.7)	168 (2.0)	<0.001
Peripheral vascular disease	7746 (6.1)	486 (5.8)	0.293
Abdominal aortic aneurysm	5965 (4.7)	301 (3.6)	<0.001
Heart Failure	19290 (15.2)	743 (8.9)	<0.001
Severe Heart Failure	3175 (2.5)	125 (1.5)	<0.001

© 2022 Keyhani S et al. *JAMA Network Open.*

Defibrillator	901 (0.7)	41 (0.5)	0.023
Atrial fibrillation	19975 (15.7)	840 (10.0)	<0.001
Cardiac arrhythmia	15113 (11.9)	677 (8.1)	<0.001
Chronic Kidney Disease (CKD)	28813 (22.7)	1171 (14.0)	<0.001
Severe CKD	2007 (1.6)	79 (0.9)	<0.001
Dialysis	461 (0.4)	19 (0.2)	0.054
Asthma	7880 (6.2)	395 (4.7)	<0.001
Bronchiectasis	620 (0.5)	19 (0.2)	0.001
Pulmonary embolism	2514 (2.0)	125 (1.5)	0.002
Deep Venous Thrombosis	3907 (3.1)	173 (2.1)	<0.001
Cirrhosis	3935 (3.1)	353 (4.2)	<0.001
Decompensated cirrhosis	1188 (0.9)	110 (1.3)	0.001
Hepatitis	3263 (2.6)	345 (4.1)	<0.001
Parkinson’s Disease	2067 (1.6)	65 (0.8)	<0.001
Extrapyramidal disease	6568 (5.2)	286 (3.4)	<0.001
Multiple Sclerosis	422 (0.3)	28 (0.3)	1.000
Seizure	1969 (1.5)	158 (1.9)	0.018
Falls	7238 (5.7)	336 (4.0)	<0.001
Orthostatic hypotension	2527 (2.0)	104 (1.2)	<0.001
Rheumatoid arthritis	4297 (3.4)	207 (2.5)	<0.001
Other inflammatory conditions	5619 (4.4)	254 (3.0)	<0.001
AIDS	287 (0.2)	59 (0.7)	<0.001
Defibrillator	901 (0.7)	41 (0.5)	0.023
External lung disease	3542 (2.8)	261 (3.1)	0.082
Bronchitis	2792 (2.2)	132 (1.6)	<0.001
Lung resection	437 (0.3)	28 (0.3)	0.963
Influenza	1328 (1.0)	61 (0.7)	0.006
Tracheostomy	607 (0.5)	28 (0.3)	0.076
Upper respiratory	12828 (10.1)	515 (6.2)	<0.001
Ventilation	9380 (7.4)	398 (4.8)	<0.001
Necrotic lung	428 (0.3)	41 (0.5)	0.027
Respiratory failure	12085 (9.5)	615 (7.3)	<0.001

© 2022 Keyhani S et al. *JAMA Network Open.*

Home oxygen within one year	5586 (4.4)	203 (2.4)	<0.001
Chronic Obstructive Lung Disease (COPD)	38214 (30.1)	2249 (26.9)	<0.001
Severe COPD	4496 (3.5)	283 (3.4)	0.468
Pulmonary fibrosis	1992 (1.6)	74 (0.9)	<0.001
Pneumonia	11529 (9.1)	516 (6.2)	<0.001
Sleep apnea	20917 (16.5)	817 (9.8)	<0.001
Dementia	13071 (10.3)	487 (5.8)	<0.001
**Cancer**			
Head and neck	1409 (1.1)	115 (1.4)	0.030
Respiratory tract	4124 (3.2)	279 (3.3)	0.685
Gastrointestinal tract	2478 (2.0)	161 (1.9)	0.896
Other Gastrointestinal tract	1160 (0.9)	139 (1.7)	<0.001
Skin	9225 (7.3)	356 (4.3)	<0.001
Genitourinary	4151 (3.3)	248 (3.0)	0.137
Prostate	10351 (8.1)	586 (7.0)	<0.001
Central Nervous System	299 (0.2)	18 (0.2)	0.799
Bone limb connective tissue	1251 (1.0)	56 (0.7)	0.005
Lymphoma/Leukemia	2216 (1.7)	130 (1.6)	0.210
Other Cancer	4264 (3.4)	365 (4.4)	<0.001
Charlson comorbidity index	4.73 (2.08)	3.83 (1.81)	<0.001
Functional impairment	16697 (13.1)	962 (11.5)	<0.001
**Body Mass Index (BMI)**			<0.001
High BMI	59351 (46.7)	2620 (31.3)	
Middle BMI	42431 (33.4)	3091 (36.9)	
Low BMI	23471 (18.5)	2573 (30.7)	
Unknown BMI	1791 (1.4)	85 (1.0)	
**Systolic blood pressure**			<0.001
Systolic blood pressure < 100	2989 (2.4)	206 (2.5)	
Systolic blood pressure 100 - 119.9	25173 (19.8)	1611 (19.2)	
Systolic blood pressure 120 - 139.9	58430 (46.0)	3709 (44.3)	
Systolic blood pressure 140 - 149.9	17147 (13.5)	1116 (13.3)	
Systolic blood pressure 150 - 159.9	10502 (8.3)	765 (9.1)	

© 2022 Keyhani S et al. *JAMA Network Open.*

Systolic blood pressure 160 - 179.9	8479 (6.7)	669 (8.0)	
Systolic blood pressure ≥180	2020 (1.6)	136 (1.6)	
Systolic blood pressure missing	2304 (1.8)	157 (1.9)	
**Albumin**			<0.001
Albumin < 2.5	155 (0.1)	15 (0.2)	
Albumin 2.5 to 3.5	5948 (4.7)	347 (4.1)	
Albumin > 3.5	37768 (29.7)	2421 (28.9)	
Albumin missing	83173 (65.5)	5586 (66.7)	
**Mental Health Conditions**			
Psychosis	3041 (2.4)	241 (2.9)	0.006
Depression	39027 (30.7)	2810 (33.6)	<0.001
Bipolar	2489 (2.0)	279 (3.3)	<0.001
PTSD	24247 (19.1)	2465 (29.5)	<0.001
Anxiety	21335 (16.8)	1353 (16.2)	0.141
Self-harm	133 (0.1)	21 (0.3)	<0.001
**Health Behaviors**			
Opioid use disorder/dependence	2914 (2.3)	324 (3.9)	<0.001
Benzodiazepines use disorder/dependence	320 (0.3)	36 (0.4)	0.003
Amphetamine use disorder/dependence	328 (0.3)	90 (1.1)	<0.001
Other drug use disorder use disorder/dependence	1445 (1.1)	267 (3.2)	<0.001
Alcohol use disorder/dependence	6526 (5.1)	856 (10.2)	<0.001
Elevated audit C score	3739 (2.9)	516 (6.2)	<0.001
Current tobacco use	37835 (29.8)	3651 (43.6)	<0.001
Positive amphetamine lab	1524 (1.2)	214 (2.6)	<0.001
Positive benzodiazepine lab	13319 (10.5)	1116 (13.3)	<0.001
Positive cocaine lab	397 (0.3)	135 (1.6)	<0.001
**Pain Disorders**			
Back and spine disorders	80960 (63.7)	4909 (58.7)	<0.001
Neck and spine disorders	26419 (20.8)	1731 (20.7)	0.818
Osteoarthritis	55191 (43.4)	2762 (33.0)	<0.001
Neuropathy	32468 (25.6)	1447 (17.3)	<0.001
Headache	12066 (9.5)	917 (11.0)	<0.001

© 2022 Keyhani S et al. *JAMA Network Open.*

Traumatic brain injury	125 (0.1)	8 (0.1)	1.000
**Social Risk**			
Homelessness/marginal housing	2068 (1.6)	307 (3.7)	<0.001
Lack of social support	94 (0.1)	8 (0.1)	0.623
**VA Priority group**			<0.001
VA Priority group 1	24081 (19.0)	1896 (22.7)	
VA Priority group 2	7166 (5.6)	458 (5.5)	
VA Priority group 3	10682 (8.4)	726 (8.7)	
VA Priority group 4	2673 (2.1)	138 (1.6)	
VA Priority group 5	36473 (28.7)	2625 (31.4)	
VA Priority group 6	3989 (3.1)	308 (3.7)	
VA Priority group 7	2170 (1.7)	119 (1.4)	
VA Priority group 8	16699 (13.1)	625 (7.5)	
VA Priority unknown	23111 (18.2)	1474 (17.6)	
**Utilization in past year**			
ICU visits within six months			0.009
ICU visits within six months (none)	122571 (96.5)	8127 (97.1)	
ICU visits within six months (one)	3861 (3.0)	206 (2.5)	
ICU visits within six months (two or more)	612 (0.5)	36 (0.4)	
ICU visits within one year			<0.001
ICU visits within one year (none)	119190 (93.8)	7972 (95.3)	
ICU visits within one year (one)	6416 (5.1)	313 (3.7)	
ICU visits within one year (two or more)	1438 (1.1)	84 (1.0)	
**Hospitalized within one year**	17148 (13.5)	1155 (13.8)	0.442
ED visits within six months			0.008
ED visits within six months (none)	97007 (76.4)	6292 (75.2)	
ED visits within six months (one)	16671 (13.1)	1197 (14.3)	
ED visits within six months (two or more)	13366 (10.5)	880 (10.5)	
ED visits within one year			<0.001
ED visits within one year (none)	84571 (66.6)	5456 (65.2)	
ED visits within one year (one)	19414 (15.3)	1429 (17.1)	
ED visits within one year (two or more)	23059 (18.2)	1484 (17.7)	

© 2022 Keyhani S et al. *JAMA Network Open.*

VA visit count	33.10 (24.07)	31.42 (22.71)	<0.001
**Psychoactive Drugs in past year**			
Morphine Equivalents per day	152.5 (348.4)	161.6 (341.01)	<0.02
Long-acting opioid	23332 (18.4)	1497 (17.9)	0.280
Alcohol use disorder treatment medication	65 (0.1)	14 (0.2)	<0.001
Benzodiazepines	24700 (19.4)	1729 (20.7)	0.007
Gaba drugs	33874 (26.7)	1852 (22.1)	<0.001
Muscle relaxants	19249 (15.2)	1323 (15.8)	0.108
Antidepressants	37244 (29.3)	2547 (30.4)	0.031
Antipsychotics	5963 (4.7)	558 (6.7)	<0.001
Sedatives	312 (0.2)	21 (0.3)	1.000
**Number of psychoactive drugs in past year**			0.376
1	51026 (40.2)	3342 (39.9)	
2	42749 (33.6)	2816 (33.6)	
3	23202 (18.3)	1565 (18.7)	
4	8235 (6.5)	511 (6.1)	
5 or more	1832 (1.4)	135 (1.6)	
**Care Assessment Need (CAN) score**			<0.001
CAN Score < 10	11 (0.0)	0 (0.0)	
CAN Score 10 - 19.9	421 (0.3)	27 (0.3)	
CAN Score 20 - 29.9	2518 (2.0)	192 (2.3)	
CAN Score 30 - 39.9	7429 (5.8)	622 (7.4)	
CAN Score 40 - 49.9	11792 (9.3)	1018 (12.2)	
CAN Score 50 - 59.9	15343 (12.1)	1215 (14.5)	
CAN Score 60 - 69.9	18126 (14.3)	1341 (16.0)	
CAN Score 70 - 79.9	22526 (17.7)	1519 (18.2)	
CAN Score 80 - 89.9	23669 (18.6)	1357 (16.2)	
CAN Score 90 - 98.9	22954 (18.1)	923 (11.0)	
CAN Score Missing	2255 (1.8)	155 (1.9)	
**Year Index Urine Drug Screen was ordered**			<0.001
2014	67344 (53.0)	3737 (44.7)	
2015	32677 (25.7)	2268 (27.1)	

© 2022 Keyhani S et al. *JAMA Network Open.*

2016	14304 (11.3)	1159 (13.8)	
2017	6607 (5.2)	586 (7.0)	
2018	4084 (3.2)	385 (4.6)	
2019	2028 (1.6)	234 (2.8)	

* Facility level variable not shown

© 2022 Keyhani S et al. *JAMA Network Open.*

eTable 7. Baseline Characteristics of 77 791 Adults Aged 65 Years and Older Receiving Long-Term Prescription Opioid Therapy (>84 Days) in 90 Days Before Urine Drug Screening by Cannabis Use (All Baseline Variables)*

	**Non-Use**	**Cannabis use**	**P<0.001**
N	72877	4914	
Age (SE)	71.37 (6.37)	68.06 (3.47)	<0.001
Male	71189 (97.7)	4861 (98.9)	<0.001
Female	1689 (2.3)	53 (1.1)	<0.001
Married	41746 (57.3)	2076 (42.2)	<0.001
**Race**			
White	61426 (84.3)	3891 (79.2)	<0.001
Black or African American	5712 (7.8)	547 (11.1)	<0.001
Asian	125 (0.2)	12 (0.2)	0.317
Native Hawaiian/Pacific Islander	579 (0.8)	49 (1.0)	0.146
American Indian or Alaska Native	715 (1.0)	75 (1.5)	<0.001
Race unknown	4320 (5.9)	340 (6.9)	0.005
**Hispanic**	1891 (2.6)	180 (3.7)	<0.001
**Clinical Conditions**			
Hypertension	59272 (81.3)	3509 (71.4)	<0.001
Hyperlipidemia	51378 (70.5)	2851 (58.0)	<0.001
Diabetes	31152 (42.7)	1455 (29.6)	<0.001
Stroke	2498 (3.4)	145 (3.0)	0.081
Transient Ischemic Attack	1925 (2.6)	72 (1.5)	<0.001
Paralytic syndromes	2185 (3.0)	132 (2.7)	0.229
Ischemic heart disease	26003 (35.7)	1161 (23.6)	<0.001
Severe ischemic heart disease	4814 (6.6)	228 (4.6)	<0.001
Myocardial infarction	7287 (10.0)	375 (7.6)	<0.001
Coronary Artery Bypass Graft	552 (0.8)	33 (0.7)	0.556
Percutaneous Coronary Intervention	1866 (2.6)	91 (1.9)	0.003
Peripheral vascular disease	3916 (5.4)	236 (4.8)	0.091
Abdominal aortic aneurysm	3376 (4.6)	165 (3.4)	<0.001
Heart Failure (CHF)	10678 (14.7)	415 (8.4)	<0.001
Severe CHF	1555 (2.1)	57 (1.2)	<0.001

© 2022 Keyhani S et al. *JAMA Network Open.*

Defibrillator	484 (0.7)	24 (0.5)	0.165
Atrial fibrillation	10615 (14.6)	448 (9.1)	<0.001
Cardiac arrhythmia	8313 (11.4)	382 (7.8)	<0.001
Chronic Kidney Disease (CKD)	15698 (21.5)	691 (14.1)	<0.001
Severe CKD	966 (1.3)	41 (0.8)	0.004
Dialysis	226 (0.3)	10 (0.2)	0.237
Asthma	4478 (6.1)	242 (4.9)	0.001
Bronchiectasis	357 (0.5)	10 (0.2)	0.006
Pulmonary embolism	1374 (1.9)	72 (1.5)	0.040
Deep Venous Thrombosis	2045 (2.8)	90 (1.8)	<0.001
Cirrhosis	2263 (3.1)	207 (4.2)	<0.001
Decompensated cirrhosis	641 (0.9)	67 (1.4)	0.001
Hepatitis	1588 (2.2)	136 (2.8)	0.008
Parkinson’s Disease	1088 (1.5)	32 (0.7)	<0.001
Extrapyramidal disease	3698 (5.1)	178 (3.6)	<0.001
Multiple Sclerosis	239 (0.3)	12 (0.2)	0.383
Seizure	1133 (1.6)	94 (1.9)	0.059
Falls	3841 (5.3)	192 (3.9)	<0.001
Orthostatic hypotension	1361 (1.9)	53 (1.1)	<0.001
Rheumatoid arthritis	2518 (3.5)	119 (2.4)	<0.001
Other inflammatory conditions	3355 (4.6)	153 (3.1)	<0.001
AIDS	175 (0.2)	34 (0.7)	<0.001
Defibrillator	484 (0.7)	24 (0.5)	0.165
External lung disease	2007 (2.8)	147 (3.0)	0.349
Bronchitis	1496 (2.1)	72 (1.5)	0.005
Lung resection	242 (0.3)	14 (0.3)	0.667
Influenza	718 (1.0)	35 (0.7)	0.069
Tracheostomy	322 (0.4)	13 (0.3)	0.085
Upper respiratory	7237 (9.9)	316 (6.4)	<0.001
Ventilation	5033 (6.9)	208 (4.2)	<0.001
Necrotic lung	248 (0.3)	26 (0.5)	0.042
Respiratory failure	6950 (9.5)	379 (7.7)	<0.001

© 2022 Keyhani S et al. *JAMA Network Open.*

Home oxygen within one year	3273 (4.5)	120 (2.4)	<0.001
Chronic Obstructive Lung Disease (COPD)	23035 (31.6)	1363 (27.7)	<0.001
Severe COPD	2460 (3.4)	148 (3.0)	0.183
Pulmonary fibrosis	1095 (1.5)	38 (0.8)	<0.001
Pneumonia	6798 (9.3)	302 (6.1)	<0.001
Sleep apnea	11347 (15.6)	439 (8.9)	<0.001
Dementia	7385 (10.1)	296 (6.0)	<0.001
**Cancer**			
Head and neck	809 (1.1)	65 (1.3)	0.194
Respiratory tract	2326 (3.2)	149 (3.0)	0.565
Gastrointestinal tract	1358 (1.9)	91 (1.9)	0.997
Other Gastrointestinal tract	606 (0.8)	67 (1.4)	<0.001
Skin	4882 (6.7)	190 (3.9)	<0.001
Genitourinary	2294 (3.1)	134 (2.7)	0.110
Prostate	5538 (7.6)	322 (6.6)	0.008
Central Nervous System	150 (0.2)	10 (0.2)	1.000
Bone limb connective tissue	673 (0.9)	30 (0.6)	0.030
Lymphoma/Leukemia	1102 (1.5)	66 (1.3)	0.378
Other Cancer	2276 (3.1)	194 (3.9)	0.002
Charlson comorbidity index	4.62 (2.04)	3.78 (1.79)	<0.001
Functional impairment	9626 (13.2)	560 (11.4)	<0.001
**Body Mass Index (BMI)**			<0.001
High BMI	33424 (45.9)	1540 (31.3)	
Middle BMI	24390 (33.5)	1789 (36.4)	
Low BMI	14154 (19.4)	1545 (31.4)	
Unknown BMI	909 (1.2)	40 (0.8)	
**Systolic blood pressure**			<0.001
Systolic blood pressure < 100	1737 (2.4)	124 (2.5)	
Systolic blood pressure 100 - 119.9	14441 (19.8)	930 (18.9)	
Systolic blood pressure 120 - 139.9	33815 (46.4)	2204 (44.9)	
Systolic blood pressure 140 - 149.9	9678 (13.3)	648 (13.2)	
Systolic blood pressure 150 - 159.9	5964 (8.2)	444 (9.0)	

© 2022 Keyhani S et al. *JAMA Network Open.*

Systolic blood pressure 160 - 179.9	4857 (6.7)	398 (8.1)	
Systolic blood pressure ≥ 180	1155 (1.6)	71 (1.4)	
Systolic blood pressure missing	1230 (1.7)	95 (1.9)	
**Albumin**			<0.001
Albumin < 2.5	69 (0.1)	7 (0.1)	
Albumin 2.5 to 3.5	3240 (4.4)	189 (3.8)	
Albumin > 3.5	22064 (30.3)	1433 (29.2)	
Albumin missing	47504 (65.2)	3285 (66.8)	
**Mental Health Conditions**			
Psychosis	1845 (2.5)	149 (3.0)	0.036
Depression	23697 (32.5)	1706 (34.7)	0.002
Bipolar	1513 (2.1)	173 (3.5)	<0.001
PTSD	14651 (20.1)	1493 (30.4)	<0.001
Anxiety	13150 (18.0)	810 (16.5)	0.006
Self-harm	83 (0.1)	10 (0.2)	0.122
**Health Behaviors**			
Opioid use disorder/dependence	2107 (2.9)	227 (4.6)	<0.001
Benzodiazepines use disorder/dependence	220 (0.3)	22 (0.4)	0.100
Amphetamine use disorder/dependence	205 (0.3)	51 (1.0)	<0.001
Other drug use disorder use disorder/dependence	987 (1.4)	169 (3.4)	<0.001
Alcohol use disorder/dependence	3812 (5.2)	467 (9.5)	<0.001
Elevated audit C score	2055 (2.8)	279 (5.7)	<0.001
Current tobacco use	24043 (33.0)	2239 (45.6)	<0.001
Positive amphetamine lab	960 (1.3)	130 (2.6)	<0.001
Positive benzodiazepine lab	8567 (11.8)	745 (15.2)	<0.001
Positive cocaine lab	215 (0.3)	62 (1.3)	<0.001
**Pain Disorders**			
Back and spine disorders	48205 (66.1)	3032 (61.7)	<0.001
Neck and spine disorders	15671 (21.5)	1044 (21.2)	0.683
Osteoarthritis	31590 (43.3)	1639 (33.4)	<0.001
Neuropathy	18738 (25.7)	845 (17.2)	<0.001
Headache	7024 (9.6)	539 (11.0)	0.003

© 2022 Keyhani S et al. *JAMA Network Open.*

Traumatic brain injury	64 (0.1)	4 (0.1)	1.000
**Social Risk**			
Homelessness/marginal housing	1178 (1.6)	164 (3.3)	<0.001
Lack of social support	31 (0.0)	3 (0.1)	0.804
**VA Priority group**			<0.001
VA Priority group 1	13905 (19.1)	1123 (22.9)	
VA Priority group 2	4019 (5.5)	255 (5.2)	
VA Priority group 3	5972 (8.2)	427 (8.7)	
VA Priority group 4	1622 (2.2)	83 (1.7)	
VA Priority group 5	21909 (30.1)	1566 (31.9)	
VA Priority group 6	2061 (2.8)	156 (3.2)	
VA Priority group 7	1101 (1.5)	64 (1.3)	
VA Priority group 8	8887 (12.2)	363 (7.4)	
VA Priority unknown	13401 (18.4)	877 (17.8)	
**Utilization in past year**			
ICU visits within six months			0.073
ICU visits within six months (none)	70623 (96.9)	4779 (97.3)	
ICU visits within six months (one)	1981 (2.7)	111 (2.3)	
ICU visits within six months (two or more)	273 (0.4)	24 (0.5)	
ICU visits within one year			<0.001
ICU visits within one year (none)	68679 (94.2)	4697 (95.6)	
ICU visits within one year (one)	3455 (4.7)	166 (3.4)	
ICU visits within one year (two or more)	743 (1.0)	51 (1.0)	
**Hospitalized within one year**	9239 (12.7)	607 (12.4)	0.521
ED visits within six months			0.001
ED visits within six months (none)	56802 (77.9)	3887 (79.1)	
ED visits within six months (one)	9136 (12.5)	636 (12.9)	
ED visits within six months (two or more)	6939 (9.5)	391 (8.0)	
ED visits within one year			0.002
ED visits within one year (none)	49257 (67.6)	3381 (68.8)	
ED visits within one year (one)	11029 (15.1)	778 (15.8)	
ED visits within one year (two or more)	12591 (17.3)	755 (15.4)	

© 2022 Keyhani S et al. *JAMA Network Open.*

VA visit count	34.19 (23.75)	31.96 (22.54)	<0.001
**Psychoactive Drugs in past year**			
Morphine Equivalents per day	222.3 (434.81)	229.1 (418.20)	0.256
Long-acting opioid	19848 (27.2)	1247 (25.4)	0.005
Alcohol use disorder treatment medication	35 (0.0)	9 (0.2)	<0.001
Benzodiazepines	16309 (22.4)	1161 (23.6)	0.044
Gaba drugs	20532 (28.2)	1122 (22.8)	<0.001
Muscle relaxants	11458 (15.7)	759 (15.4)	0.620
Antidepressants	22728 (31.2)	1575 (32.1)	0.211
Antipsychotics	3822 (5.2)	361 (7.3)	<0.001
Sedatives	142 (0.2)	10 (0.2)	1.000
**Number of psychoactive drugs in past year**			0.627
1	26942 (37.0)	1853 (37.7)	
2	24903 (34.2)	1672 (34.0)	
3	14387 (19.7)	953 (19.4)	
4	5377 (7.4)	343 (7.0)	
5 or more	1268 (1.7)	93 (1.9)	
**Care Assessment Need (CAN) score**			<0.001
CAN Score < 10	7 (0.0)	0 (0.0)	
CAN Score 10 - 19.9	222 (0.3)	12 (0.2)	
CAN Score 20 - 29.9	1318 (1.8)	102 (2.1)	
CAN Score 30 - 39.9	4206 (5.8)	362 (7.4)	
CAN Score 40 - 49.9	6803 (9.3)	590 (12.0)	
CAN Score 50 - 59.9	8982 (12.3)	729 (14.8)	
CAN Score 60 - 69.9	10646 (14.6)	812 (16.5)	
CAN Score 70 - 79.9	13295 (18.2)	899 (18.3)	
CAN Score 80 - 89.9	13681 (18.8)	818 (16.6)	
CAN Score 90 - 98.9	12744 (17.5)	518 (10.5)	
CAN Score Missing	973 (1.3)	72 (1.5)	
**Year Index Urine Drug Screen was ordered**			<0.001
2014	44264 (60.7)	2533 (51.5)	
2015	17760 (24.4)	1385 (28.2)	

© 2022 Keyhani S et al. *JAMA Network Open.*

2016	6631 (9.1)	563 (11.5)	
2017	2421 (3.3)	242 (4.9)	
2018	1382 (1.9)	137 (2.8)	
2019	419 (0.6)	54 (1.1)	

* Facility level variable not shown

© 2022 Keyhani S et al. *JAMA Network Open.*

eTable 8. Propensity-Weighted and Matched Adults Aged 65 Years and Older Receiving Any Prescription Opioid Therapy in Past 90 Days (Full List of Variables)*

	Propensity Weighted			Propensity Matched		
	**Non-use**	**Cannabis use**	**Diff weighted**	**Non-use**	**Cannabis use**	**Diff matched**
N	8494.63	8369		8369	8369	
Age	68.29 (3.7)	68.33 (3.72)	0.01	68.29 (3.72)	68.33 (3.72)	0.011
Male	8400 (98.89)	8274 (98.86)	0	8270 (98.82)	8274 (98.86)	0.004
Female	95 (1.11)	95 (1.14)	0	99 (1.18)	95 (1.14)	-0.004
Married	3583 (42.18)	3598 (42.99)	0.008	3625 (43.31)	3598 (42.99)	-0.007
**Race**						
White	6503 (76.55)	6490 (77.55)	0.01	6466 (77.26)	6490 (77.55)	0.007
Black or African American	1181 (13.9)	1085 (12.96)	-0.009	1113 (13.3)	1085 (12.96)	-0.01
Asian	21 (0.24)	21 (0.25)	0	17 (0.2)	21 (0.25)	0.01
Native Hawaiian or other Pacific Islander	82 (0.96)	78 (0.93)	0	73 (0.87)	78 (0.93)	0.006
American Indian or Alaska Native	122 (1.43)	120 (1.43)	0	127 (1.52)	120 (1.43)	-0.007
Race unknown	587 (6.91)	575 (6.87)	0	573 (6.85)	575 (6.87)	0.001
**Hispanic**	324 (3.81)	316 (3.78)	0	331 (3.96)	316 (3.78)	-0.009
**Clinical Conditions**						
Hypertension	6080 (71.57)	5970 (71.33)	-0.002	5923 (70.77)	5970 (71.33)	0.012
Hyperlipidemia	4861 (57.22)	4847 (57.92)	0.007	4839 (57.82)	4847 (57.92)	0.002
Diabetes	2526 (29.74)	2480 (29.63)	-0.001	2426 (28.99)	2480 (29.63)	0.014
Stroke	247 (2.91)	240 (2.87)	0	238 (2.84)	240 (2.87)	0.001
Transient Ischemic Attack	136 (1.6)	134 (1.6)	0	131 (1.57)	134 (1.6)	0.003
Paralytic syndromes	218 (2.56)	216 (2.58)	0	205 (2.45)	216 (2.58)	0.008
Ischemic heart disease	1996 (23.5)	1986 (23.73)	0.002	1998 (23.87)	1986 (23.73)	-0.003
Severe ischemic heart disease	452 (5.32)	443 (5.29)	0	424 (5.07)	443 (5.29)	0.01
Myocardial infarction	650 (7.65)	644 (7.7)	0	650 (7.77)	644 (7.7)	-0.003
Coronary Artery Bypass Graft	63 (0.74)	63 (0.75)	0	62 (0.74)	63 (0.75)	0.001
Percutaneous Coronary Intervention	165 (1.94)	168 (2.01)	0.001	171 (2.04)	168 (2.01)	-0.003
Peripheral vascular disease	500 (5.88)	486 (5.81)	-0.001	503 (6.01)	486 (5.81)	-0.009
Abdominal aortic aneurysm	306 (3.6)	301 (3.6)	0	300 (3.58)	301 (3.6)	0.001
Heart Failure (CHF)	756 (8.9)	743 (8.88)	0	759 (9.07)	743 (8.88)	-0.007
Severe CHF	126 (1.49)	125 (1.49)	0	115 (1.37)	125 (1.49)	0.01
Atrial fibrillation	851 (10.02)	840 (10.04)	0	833 (9.95)	840 (10.04)	0.003

© 2022 Keyhani S et al. *JAMA Network Open.*

	Propensity Weighted			Propensity Matched		
	**Non-use**	**Cannabis use**	**Diff weighted**	**Non-use**	**Cannabis use**	**Diff matched**
Cardiac arrhythmia	688 (8.1)	677 (8.09)	0	667 (7.97)	677 (8.09)	0.004
Chronic Kidney Disease (CKD)	1178 (13.87)	1171 (13.99)	0.001	1140 (13.62)	1171 (13.99)	0.011
Severe CKD	80 (0.94)	79 (0.94)	0	80 (0.96)	79 (0.94)	-0.001
Dialysis	19 (0.22)	19 (0.23)	0	19 (0.23)	19 (0.23)	0
Asthma	405 (4.77)	395 (4.72)	-0.001	376 (4.49)	395 (4.72)	0.011
Bronchiectasis	20 (0.23)	19 (0.23)	0	13 (0.16)	19 (0.23)	0.016
Pulmonary embolism	127 (1.5)	125 (1.49)	0	119 (1.42)	125 (1.49)	0.006
Deep Venous Thrombosis	177 (2.08)	173 (2.07)	0	184 (2.2)	173 (2.07)	-0.009
Cirrhosis	379 (4.46)	353 (4.22)	-0.002	327 (3.91)	353 (4.22)	0.016
Decompensated cirrhosis	113 (1.33)	110 (1.31)	0	94 (1.12)	110 (1.31)	0.017
Hepatitis	363 (4.28)	345 (4.12)	-0.002	313 (3.74)	345 (4.12)	0.02
Parkinson's Disease	65 (0.77)	65 (0.78)	0	59 (0.7)	65 (0.78)	0.008
Extrapyramidal disease	297 (3.5)	286 (3.42)	-0.001	315 (3.76)	286 (3.42)	-0.019
Multiple Sclerosis	28 (0.33)	28 (0.33)	0	29 (0.35)	28 (0.33)	-0.002
Seizure	170 (2)	158 (1.89)	-0.001	140 (1.67)	158 (1.89)	0.016
Falls	361 (4.25)	336 (4.01)	-0.002	327 (3.91)	336 (4.01)	0.006
Orthostatic hypotension	109 (1.28)	104 (1.24)	0	97 (1.16)	104 (1.24)	0.008
Rheumatoid arthritis	210 (2.47)	207 (2.47)	0	209 (2.5)	207 (2.47)	-0.002
Other inflammatory conditions	254 (2.99)	254 (3.04)	0	222 (2.65)	254 (3.04)	0.023
AIDS	72 (0.85)	59 (0.7)	-0.001	65 (0.78)	59 (0.7)	-0.008
Defibrillator	42 (0.49)	41 (0.49)	0	47 (0.56)	41 (0.49)	-0.01
External lung disease	274 (3.22)	261 (3.12)	-0.001	244 (2.92)	261 (3.12)	0.012
Bronchitis	131 (1.54)	132 (1.58)	0	131 (1.57)	132 (1.58)	0.001
Lung resection	30 (0.35)	28 (0.33)	0	32 (0.38)	28 (0.33)	-0.008
Influenza	63 (0.74)	61 (0.73)	0	61 (0.73)	61 (0.73)	0
Tracheostomy	30 (0.35)	28 (0.33)	0	23 (0.27)	28 (0.33)	0.011
Upper respiratory	524 (6.17)	515 (6.15)	0	510 (6.09)	515 (6.15)	0.002
Ventilation	405 (4.77)	398 (4.76)	0	403 (4.82)	398 (4.76)	-0.003
Necrotic lung	39 (0.46)	41 (0.49)	0	43 (0.51)	41 (0.49)	-0.003
Respiratory failure	653 (7.69)	615 (7.35)	-0.003	612 (7.31)	615 (7.35)	0.001
Home oxygen within one year	207 (2.44)	203 (2.43)	0	221 (2.64)	203 (2.43)	-0.014
Chronic Obstructive Lung Disease (COPD)	2310 (27.2)	2249 (26.87)	-0.003	2207 (26.37)	2249 (26.87)	0.011
Severe COPD	296 (3.49)	283 (3.38)	-0.001	284 (3.39)	283 (3.38)	-0.001

© 2022 Keyhani S et al. *JAMA Network Open.*

	Propensity Weighted			Propensity Matched		
	**Non-use**	**Cannabis use**	**Diff weighted**	**Non-use**	**Cannabis use**	**Diff matched**
Pulmonary fibrosis	77 (0.91)	74 (0.88)	0	75 (0.9)	74 (0.88)	-0.001
Pneumonia	532 (6.27)	516 (6.17)	-0.001	530 (6.33)	516 (6.17)	-0.007
Sleep apnea	829 (9.76)	817 (9.76)	0	806 (9.63)	817 (9.76)	0.004
Dementia	491 (5.77)	487 (5.82)	0	452 (5.4)	487 (5.82)	0.018
**Cancer**						
Head and neck	114 (1.35)	115 (1.37)	0	90 (1.08)	115 (1.37)	0.027
Lung respiratory tract	284 (3.34)	279 (3.33)	0	268 (3.2)	279 (3.33)	0.007
Gastrointestinal tract	166 (1.95)	161 (1.92)	0	173 (2.07)	161 (1.92)	-0.01
Other Gastrointestinal tract	148 (1.74)	139 (1.66)	-0.001	132 (1.58)	139 (1.66)	0.007
Skin	356 (4.19)	356 (4.25)	0.001	343 (4.1)	356 (4.25)	0.008
Genitourinary	248 (2.91)	248 (2.96)	0	224 (2.68)	248 (2.96)	0.017
Prostate	615 (7.23)	586 (7)	-0.002	592 (7.07)	586 (7)	-0.003
Central Nervous System	18 (0.21)	18 (0.22)	0	20 (0.24)	18 (0.22)	-0.005
Bone limb connective tissue	58 (0.68)	56 (0.67)	0	67 (0.8)	56 (0.67)	-0.015
Lymphoma/Leukemia	131 (1.54)	130 (1.55)	0	132 (1.58)	130 (1.55)	-0.002
Other Cancer	375 (4.42)	365 (4.36)	-0.001	349 (4.17)	365 (4.36)	0.009
Charlson comorbidity index	3.84 (1.83)	3.83 (1.81)	-0.008	3.8 (1.79)	3.83 (1.81)	0.017
Functional impairment	983 (11.57)	962 (11.49)	-0.001	971 (11.6)	962 (11.49)	-0.003
**Body Mass Index (BMI)**						
High BMI	2622 (30.87)	2620 (31.31)	0.004	2624 (31.35)	2620 (31.31)	-0.001
Middle BMI	3149 (37.06)	3091 (36.93)	-0.001	3116 (37.23)	3091 (36.93)	-0.006
Low BMI	2635 (31.01)	2573 (30.74)	-0.003	2546 (30.42)	2573 (30.74)	0.007
Unknown BMI	89 (1.05)	85 (1.02)	0	83 (0.99)	85 (1.02)	0.002
**Systolic Blood Pressure**						
Systolic Blood Pressure < 100	215 (2.54)	206 (2.46)	-0.001	210 (2.51)	206 (2.46)	-0.003
Systolic Blood Pressure 100 - 119.9	1637 (19.27)	1611 (19.25)	0	1553 (18.56)	1611 (19.25)	0.018
Systolic Blood Pressure 120 - 139.9	3765 (44.33)	3709 (44.32)	0	3824 (45.69)	3709 (44.32)	-0.028
Systolic Blood Pressure 140 - 149.9	1136 (13.37)	1116 (13.33)	0	1101 (13.16)	1116 (13.33)	0.005
Systolic Blood Pressure 150 - 159.9	751 (8.84)	765 (9.14)	0.003	762 (9.11)	765 (9.14)	0.001
Systolic Blood Pressure 160 - 179.9	683 (8.04)	669 (7.99)	0	629 (7.52)	669 (7.99)	0.018
Systolic Blood Pressure ≥ 180	147 (1.73)	136 (1.63)	-0.001	139 (1.66)	136 (1.63)	-0.003
Systolic Blood Pressure Missing	160 (1.88)	157 (1.88)	0	151 (1.8)	157 (1.88)	0.005
**Albumin**						

© 2022 Keyhani S et al. *JAMA Network Open.*

	Propensity Weighted			Propensity Matched		
	**Non-use**	**Cannabis use**	**Diff weighted**	**Non-use**	**Cannabis use**	**Diff matched**
Albumin < 2.5	15 (0.18)	15 (0.18)	0	11 (0.13)	15 (0.18)	0.012
Albumin 2.5 to 3.5	350 (4.12)	347 (4.15)	0	323 (3.86)	347 (4.15)	0.015
Albumin > 3.5	2460 (28.96)	2421 (28.93)	0	2453 (29.31)	2421 (28.93)	-0.008
Albumin missing	5669 (66.74)	5586 (66.75)	0	5582 (66.7)	5586 (66.75)	0.001
Low hemoglobin	174 (2.04)	166 (1.98)	-0.001	157 (1.88)	166 (1.98)	0.008
**Mental Health Conditions**						
Psychosis	248 (2.92)	241 (2.88)	0	245 (2.93)	241 (2.88)	-0.003
Depression	2855 (33.61)	2810 (33.58)	0	2807 (33.54)	2810 (33.58)	0.001
Bipolar	287 (3.38)	279 (3.33)	0	293 (3.5)	279 (3.33)	-0.009
PTSD	2490 (29.31)	2465 (29.45)	0.001	2484 (29.68)	2465 (29.45)	-0.005
Anxiety	1370 (16.12)	1353 (16.17)	0	1366 (16.32)	1353 (16.17)	-0.004
Self-harm	26 (0.3)	21 (0.25)	-0.001	26 (0.31)	21 (0.25)	-0.011
**Health Behaviors**						
Opioid use disorder/dependence	351 (4.13)	324 (3.87)	-0.003	338 (4.04)	324 (3.87)	-0.009
Benzodiazepine use disorder/dependence	47 (0.55)	36 (0.43)	-0.001	38 (0.45)	36 (0.43)	-0.004
Amphetamine use disorder/dependence	117 (1.38)	90 (1.08)	-0.003	90 (1.08)	90 (1.08)	0
Other drug use disorder/dependence	317 (3.74)	267 (3.19)	-0.005	268 (3.2)	267 (3.19)	-0.001
Alcohol use disorder	922 (10.86)	856 (10.23)	-0.006	834 (9.97)	856 (10.23)	0.009
Elevated audit C score	542 (6.38)	516 (6.17)	-0.002	487 (5.82)	516 (6.17)	0.015
Current tobacco use	3757 (44.23)	3651 (43.63)	-0.006	3631 (43.39)	3651 (43.63)	0.005
Positive amphetamine lab	238 (2.8)	214 (2.56)	-0.002	206 (2.46)	214 (2.56)	0.006
Positive benzodiazepine lab	1144 (13.47)	1116 (13.33)	-0.001	1172 (14)	1116 (13.33)	-0.019
Positive cocaine lab	187 (2.21)	135 (1.61)	-0.006	118 (1.41)	135 (1.61)	0.017
**Pain Disorders**						
Back and spine disorders	4970 (58.51)	4909 (58.66)	0.001	4897 (58.51)	4909 (58.66)	0.003
Neck and spine disorders	1746 (20.55)	1731 (20.68)	0.001	1731 (20.68)	1731 (20.68)	0
Osteoarthritis	2776 (32.68)	2762 (33)	0.003	2725 (32.56)	2762 (33)	0.009
Neuropathy	1455 (17.13)	1447 (17.29)	0.002	1443 (17.24)	1447 (17.29)	0.001
Headache	970 (11.42)	917 (10.96)	-0.005	891 (10.65)	917 (10.96)	0.01
Traumatic brain injury	7 (0.09)	8 (0.1)	0	3 (0.04)	8 (0.1)	0.023
**Social Risk**						
Homelessness	356 (4.19)	307 (3.67)	-0.005	306 (3.66)	307 (3.67)	0.001
Lack of social support	8 (0.09)	8 (0.1)	0	16 (0.19)	8 (0.1)	-0.025

© 2022 Keyhani S et al. *JAMA Network Open.*

	Propensity Weighted			Propensity Matched		
	**Non-use**	**Cannabis use**	**Diff weighted**	**Non-use**	**Cannabis use**	**Diff matched**
**VA Priority group**						
VA Priority group 1	1909 (22.47)	1896 (22.66)	0.002	1928 (23.04)	1896 (22.66)	-0.009
VA Priority group 2	470 (5.53)	458 (5.47)	-0.001	497 (5.94)	458 (5.47)	-0.02
VA Priority group 3	737 (8.68)	726 (8.67)	0	709 (8.47)	726 (8.67)	0.007
VA Priority group 4	140 (1.65)	138 (1.65)	0	137 (1.64)	138 (1.65)	0.001
VA Priority group 5	2693 (31.71)	2625 (31.37)	-0.003	2602 (31.09)	2625 (31.37)	0.006
VA Priority group 6	310 (3.64)	308 (3.68)	0	321 (3.84)	308 (3.68)	-0.008
VA Priority group 7	117 (1.38)	119 (1.42)	0	115 (1.37)	119 (1.42)	0.004
VA Priority group 8	634 (7.46)	625 (7.47)	0	617 (7.37)	625 (7.47)	0.004
VA Priority unknown	1485 (17.48)	1474 (17.61)	0.001	1443 (17.24)	1474 (17.61)	0.01
**Utilization in past year**						
ICU visits within six months						
ICU visits within six months (none)	8249 (97.1)	8127 (97.11)	0	8124 (97.07)	8127 (97.11)	0.002
ICU visits within six months (one)	210 (2.47)	206 (2.46)	0	207 (2.47)	206 (2.46)	-0.001
ICU visits within six months (two or more)	36 (0.42)	36 (0.43)	0	38 (0.45)	36 (0.43)	-0.004
ICU visits within one year						
ICU visits within one year (none)	8093 (95.28)	7972 (95.26)	0	7984 (95.4)	7972 (95.26)	-0.007
ICU visits within one year (one)	314 (3.7)	313 (3.74)	0	291 (3.48)	313 (3.74)	0.014
ICU visits within one year (two or more)	87 (1.03)	84 (1)	0	94 (1.12)	84 (1)	-0.012
**Hospitalized within one year**	1182 (13.91)	1155 (13.8)	-0.001	1137 (13.59)	1155 (13.8)	0.006
ED visits within six months						
ED visits within six months (none)	6342 (74.65)	6292 (75.18)	0.005	6276 (74.99)	6292 (75.18)	0.004
ED visits within six months (one)	1235 (14.54)	1197 (14.3)	-0.002	1214 (14.51)	1197 (14.3)	-0.006
ED visits within six months (two or more)	918 (10.81)	880 (10.51)	-0.003	879 (10.5)	880 (10.51)	0
ED visits within one year						
ED visits within one year (none)	5508 (64.84)	5456 (65.19)	0.004	5440 (65)	5456 (65.19)	0.004
ED visits within one year (one)	1453 (17.11)	1429 (17.07)	0	1428 (17.06)	1429 (17.07)	0
ED visits within one year (two or more)	1533 (18.05)	1484 (17.73)	-0.003	1501 (17.94)	1484 (17.73)	-0.005
VA visit count	31.48 (22.52)	31.42 (22.71)	-0.002	31.63 (22.46)	31.42 (22.71)	-0.009
**Psychoactive Drugs in past year**						
Morphine Equivalents over prior 90-day period	161.86 (346.93)	161.64 (341.01)	-0.001	158.93 (330.42)	161.64 (341.01)	0.008
Long-acting opioid	1493 (17.57)	1497 (17.89)	0.003	1471 (17.58)	1497 (17.89)	0.008

© 2022 Keyhani S et al. *JAMA Network Open.*

	Propensity Weighted			Propensity Matched		
	**Non-use**	**Cannabis use**	**Diff weighted**	**Non-use**	**Cannabis use**	**Diff matched**
Alcohol use disorder treatment medication	12 (0.14)	14 (0.17)	0	17 (0.2)	14 (0.17)	-0.008
Benzodiazepines	1757 (20.68)	1729 (20.66)	0	1808 (21.6)	1729 (20.66)	-0.023
Gaba drugs	1873 (22.04)	1852 (22.13)	0.001	1911 (22.83)	1852 (22.13)	-0.017
Muscle relaxants	1341 (15.78)	1323 (15.81)	0	1335 (15.95)	1323 (15.81)	-0.004
Antidepressants	2566 (30.2)	2547 (30.43)	0.002	2537 (30.31)	2547 (30.43)	0.003
Antipsychotics	561 (6.61)	558 (6.67)	0.001	557 (6.66)	558 (6.67)	0
Sedatives	21 (0.25)	21 (0.25)	0	17 (0.2)	21 (0.25)	0.01
**Number of psychoactive drugs in past year**						
1	3408 (40.12)	3342 (39.93)	-0.002	3306 (39.5)	3342 (39.93)	0.009
2	2857 (33.63)	2816 (33.65)	0	2799 (33.44)	2816 (33.65)	0.004
3	1574 (18.53)	1565 (18.7)	0.002	1582 (18.9)	1565 (18.7)	-0.005
4	520 (6.12)	511 (6.11)	0	535 (6.39)	511 (6.11)	-0.012
5	136 (1.6)	135 (1.61)	0	147 (1.76)	135 (1.61)	-0.011
**Care Assessment Need (CAN) score**						
Care Assessment Need (CAN) score < 10	0 (0)	0 (0)	0			
CAN score 10 - 19.9	27 (0.31)	27 (0.32)	0	24 (0.29)	27 (0.32)	0.007
CAN score 20 - 29.9	196 (2.3)	192 (2.29)	0	215 (2.57)	192 (2.29)	-0.018
CAN score 30 - 39.9	625 (7.35)	622 (7.43)	0.001	636 (7.6)	622 (7.43)	-0.006
CAN score 40 - 49.9	1021 (12.01)	1018 (12.16)	0.001	1006 (12.02)	1018 (12.16)	0.004
CAN score 50 - 59.9	1224 (14.41)	1215 (14.52)	0.001	1218 (14.55)	1215 (14.52)	-0.001
CAN score 60 - 69.9	1355 (15.95)	1341 (16.02)	0.001	1321 (15.78)	1341 (16.02)	0.007
CAN score 70 - 79.9	1550 (18.25)	1519 (18.15)	-0.001	1513 (18.08)	1519 (18.15)	0.002
CAN score 80 - 89.9	1380 (16.25)	1357 (16.21)	0	1334 (15.94)	1357 (16.21)	0.007
CAN score 90 - 98.9	955 (11.24)	923 (11.03)	-0.002	950 (11.35)	923 (11.03)	-0.01
CAN score missing	164 (1.93)	155 (1.85)	-0.001	152 (1.82)	155 (1.85)	0.003
**Year Index Urine Drug Screen was ordered**						
2014	3772 (44.4)	3737 (44.65)	0.002	3753 (44.84)	3737 (44.65)	-0.004
2015	2283 (26.87)	2268 (27.1)	0.002	2244 (26.81)	2268 (27.1)	0.006
2016	1192 (14.03)	1159 (13.85)	-0.002	1153 (13.78)	1159 (13.85)	0.002
2017	607 (7.14)	586 (7)	-0.001	591 (7.06)	586 (7)	-0.002
2018	391 (4.61)	385 (4.6)	0	387 (4.62)	385 (4.6)	-0.001
2019	250 (2.94)	234 (2.8)	-0.001	241 (2.88)	234 (2.8)	-0.005

© 2022 Keyhani S et al. *JAMA Network Open.*

* Facility level variable not shown

© 2022 Keyhani S et al. *JAMA Network Open.*

eTable 9. Propensity-Weighted and Matched Veterans Aged 65 Years and Older Receiving Long-Term (>84 Days) Prescription Opioid Therapy in Past 90 Days (Full List of Variables)*

	Propensity Weighted			Propensity Matched		
	**Non-use**	**Cannabis use**	**Diff weighted**	**Non-use**	**Cannabis use**	**Diff matched**
N	4970.58	4914		4914	4914	
Age	68.04 (3.45)	68.06 (3.47)	0.007	68 (3.37)	68.06 (3.47)	0.018
Male	4918 (98.94)	4861 (98.92)	0	4860 (98.9)	4861 (98.92)	0.002
Female	53 (1.06)	53 (1.08)	0	54 (1.1)	53 (1.08)	-0.002
Married	2061 (41.47)	2076 (42.25)	0.008	2096 (42.65)	2076 (42.25)	-0.008
**Race**						
White	3910 (78.66)	3891 (79.18)	0.005	3865 (78.65)	3891 (79.18)	0.013
Black or African American	575 (11.56)	547 (11.13)	-0.004	563 (11.46)	547 (11.13)	-0.01
Asian	12 (0.24)	12 (0.24)	0	12 (0.24)	12 (0.24)	0
Native Hawaiian or other Pacific Islander	52 (1.04)	49 (1)	0	49 (1)	49 (1)	0
American Indian or Alaska Native	76 (1.54)	75 (1.53)	0	84 (1.71)	75 (1.53)	-0.015
Race unknown	346 (6.96)	340 (6.92)	0	341 (6.94)	340 (6.92)	-0.001
**Hispanic**	184 (3.7)	180 (3.66)	0	177 (3.6)	180 (3.66)	0.003
**Clinical Conditions**						
Hypertension	3567 (71.77)	3509 (71.41)	-0.004	3478 (70.78)	3509 (71.41)	0.014
Hyperlipidemia	2858 (57.5)	2851 (58.02)	0.005	2873 (58.47)	2851 (58.02)	-0.009
Diabetes	1478 (29.74)	1455 (29.61)	-0.001	1512 (30.77)	1455 (29.61)	-0.025
Stroke	150 (3.01)	145 (2.95)	-0.001	156 (3.17)	145 (2.95)	-0.013
Transient Ischemic Attack	72 (1.45)	72 (1.47)	0	81 (1.65)	72 (1.47)	-0.015
Paralytic syndromes	132 (2.66)	132 (2.69)	0	124 (2.52)	132 (2.69)	0.01
Ischemic heart disease	1166 (23.45)	1161 (23.63)	0.002	1192 (24.26)	1161 (23.63)	-0.015
Severe ischemic heart disease	233 (4.68)	228 (4.64)	0	241 (4.9)	228 (4.64)	-0.012
Myocardial infarction	378 (7.61)	375 (7.63)	0	356 (7.24)	375 (7.63)	0.015
Coronary Artery Bypass Graft	33 (0.66)	33 (0.67)	0	37 (0.75)	33 (0.67)	-0.01
Percutaneous Coronary Intervention	88 (1.78)	91 (1.85)	0.001	90 (1.83)	91 (1.85)	0.002
Peripheral vascular disease	247 (4.97)	236 (4.8)	-0.002	245 (4.99)	236 (4.8)	-0.008
Abdominal aortic aneurysm	165 (3.32)	165 (3.36)	0	163 (3.32)	165 (3.36)	0.002
Heart Failure (CHF)	426 (8.56)	415 (8.45)	-0.001	415 (8.45)	415 (8.45)	0
Severe CHF	58 (1.17)	57 (1.16)	0	56 (1.14)	57 (1.16)	0.002
Atrial fibrillation	455 (9.16)	448 (9.12)	0	459 (9.34)	448 (9.12)	-0.008

© 2022 Keyhani S et al. *JAMA Network Open.*

	Propensity Weighted			Propensity Matched		
	Non-use	Cannabis use	Diff weighted	Non-use	Cannabis use	Diff matched
Cardiac arrhythmia	389 (7.82)	382 (7.77)	-0.001	382 (7.77)	382 (7.77)	0
Chronic Kidney Disease (CKD)	696 (14.01)	691 (14.06)	0.001	679 (13.82)	691 (14.06)	0.007
Severe CKD	43 (0.87)	41 (0.83)	0	47 (0.96)	41 (0.83)	-0.013
Dialysis	10 (0.2)	10 (0.2)	0	6 (0.12)	10 (0.2)	0.02
Asthma	256 (5.14)	242 (4.92)	-0.002	210 (4.27)	242 (4.92)	0.031
Bronchiectasis	9 (0.19)	10 (0.2)	0	5 (0.1)	10 (0.2)	0.026
Pulmonary embolism	72 (1.45)	72 (1.47)	0	50 (1.02)	72 (1.47)	0.04
Deep Venous Thrombosis	94 (1.9)	90 (1.83)	-0.001	97 (1.97)	90 (1.83)	-0.01
Cirrhosis	218 (4.39)	207 (4.21)	-0.002	211 (4.29)	207 (4.21)	-0.004
Decompensated cirrhosis	72 (1.44)	67 (1.36)	-0.001	69 (1.4)	67 (1.36)	-0.003
Hepatitis	143 (2.88)	136 (2.77)	-0.001	127 (2.58)	136 (2.77)	0.011
Parkinson's Disease	32 (0.65)	32 (0.65)	0	27 (0.55)	32 (0.65)	0.013
Extrapyramidal disease	187 (3.77)	178 (3.62)	-0.001	175 (3.56)	178 (3.62)	0.003
Multiple Sclerosis	12 (0.24)	12 (0.24)	0	15 (0.31)	12 (0.24)	-0.012
Seizure	96 (1.93)	94 (1.91)	0	85 (1.73)	94 (1.91)	0.014
Falls	198 (3.99)	192 (3.91)	-0.001	201 (4.09)	192 (3.91)	-0.009
Orthostatic hypotension	56 (1.13)	53 (1.08)	-0.001	62 (1.26)	53 (1.08)	-0.017
Rheumatoid arthritis	122 (2.46)	119 (2.42)	0	124 (2.52)	119 (2.42)	-0.007
Other inflammatory conditions	153 (3.07)	153 (3.11)	0	169 (3.44)	153 (3.11)	-0.018
AIDS	43 (0.87)	34 (0.69)	-0.002	32 (0.65)	34 (0.69)	0.005
Defibrillator	24 (0.47)	24 (0.49)	0	25 (0.51)	24 (0.49)	-0.003
External lung disease	160 (3.21)	147 (2.99)	-0.002	144 (2.93)	147 (2.99)	0.004
Bronchitis	67 (1.35)	72 (1.47)	0.001	80 (1.63)	72 (1.47)	-0.013
Lung resection	14 (0.29)	14 (0.28)	0	12 (0.24)	14 (0.28)	0.008
Influenza	37 (0.74)	35 (0.71)	0	38 (0.77)	35 (0.71)	-0.007
Tracheostomy	13 (0.27)	13 (0.26)	0	7 (0.14)	13 (0.26)	0.027
Upper respiratory	318 (6.4)	316 (6.43)	0	345 (7.02)	316 (6.43)	-0.024
Ventilation	211 (4.24)	208 (4.23)	0	190 (3.87)	208 (4.23)	0.019
Necrotic lung	25 (0.49)	26 (0.53)	0	24 (0.49)	26 (0.53)	0.006
Respiratory failure	403 (8.11)	379 (7.71)	-0.004	374 (7.61)	379 (7.71)	0.004
Home oxygen within one year	121 (2.43)	120 (2.44)	0	109 (2.22)	120 (2.44)	0.015
Chronic Obstructive Lung Disease (COPD)	1396 (28.09)	1363 (27.74)	-0.004	1393 (28.35)	1363 (27.74)	-0.014
Severe COPD	159 (3.19)	148 (3.01)	-0.002	147 (2.99)	148 (3.01)	0.001

© 2022 Keyhani S et al. *JAMA Network Open.*

	Propensity Weighted			Propensity Matched		
	**Non-use**	**Cannabis use**	**Diff weighted**	**Non-use**	**Cannabis use**	**Diff matched**
Pulmonary fibrosis	39 (0.79)	38 (0.77)	0	53 (1.08)	38 (0.77)	-0.032
Pneumonia	313 (6.29)	302 (6.15)	-0.001	315 (6.41)	302 (6.15)	-0.011
Sleep apnea	444 (8.93)	439 (8.93)	0	413 (8.4)	439 (8.93)	0.019
Dementia	299 (6.02)	296 (6.02)	0	273 (5.56)	296 (6.02)	0.02
**Cancer**						
Head and neck	66 (1.33)	65 (1.32)	0	70 (1.42)	65 (1.32)	-0.009
Lung respiratory tract	151 (3.03)	149 (3.03)	0	144 (2.93)	149 (3.03)	0.006
Gastrointestinal tract	97 (1.96)	91 (1.85)	-0.001	99 (2.01)	91 (1.85)	-0.012
Other Gastrointestinal tract	68 (1.37)	67 (1.36)	0	79 (1.61)	67 (1.36)	-0.02
Skin	189 (3.79)	190 (3.87)	0.001	182 (3.7)	190 (3.87)	0.009
Genitourinary	135 (2.72)	134 (2.73)	0	130 (2.65)	134 (2.73)	0.005
Prostate	333 (6.69)	322 (6.55)	-0.001	308 (6.27)	322 (6.55)	0.012
Central Nervous System	9 (0.19)	10 (0.2)	0	9 (0.18)	10 (0.2)	0.005
Bone limb connective tissue	30 (0.6)	30 (0.61)	0	36 (0.73)	30 (0.61)	-0.015
Lymphoma/Leukemia	66 (1.32)	66 (1.34)	0	67 (1.36)	66 (1.34)	-0.002
Other Cancer	200 (4.02)	194 (3.95)	-0.001	193 (3.93)	194 (3.95)	0.001
Charlson comorbidity index	3.81 (1.83)	3.78 (1.79)	-0.012	3.79 (1.77)	3.78 (1.79)	-0.001
Functional impairment	574 (11.55)	560 (11.4)	-0.002	581 (11.82)	560 (11.4)	-0.013
**Body Mass Index (BMI)**						
High BMI	1540 (30.97)	1540 (31.34)	0.004	1501 (30.55)	1540 (31.34)	0.017
Middle BMI	1818 (36.57)	1789 (36.41)	-0.002	1808 (36.79)	1789 (36.41)	-0.008
Low BMI	1572 (31.63)	1545 (31.44)	-0.002	1579 (32.13)	1545 (31.44)	-0.015
Unknown BMI	41 (0.82)	40 (0.81)	0	26 (0.53)	40 (0.81)	0.035
**Systolic Blood Pressure**						
Systolic Blood Pressure < 100	132 (2.66)	124 (2.52)	-0.001	117 (2.38)	124 (2.52)	0.009
Systolic Blood Pressure 100 - 119.9	942 (18.96)	930 (18.93)	0	939 (19.11)	930 (18.93)	-0.005
Systolic Blood Pressure 120 - 139.9	2220 (44.66)	2204 (44.85)	0.002	2172 (44.2)	2204 (44.85)	0.013
Systolic Blood Pressure 140 - 149.9	655 (13.18)	648 (13.19)	0	642 (13.06)	648 (13.19)	0.004
Systolic Blood Pressure 150 - 159.9	435 (8.75)	444 (9.04)	0.003	447 (9.1)	444 (9.04)	-0.002
Systolic Blood Pressure 160 - 179.9	412 (8.28)	398 (8.1)	-0.002	414 (8.42)	398 (8.1)	-0.012
Systolic Blood Pressure ≥ 180	78 (1.58)	71 (1.44)	-0.001	74 (1.51)	71 (1.44)	-0.005
Systolic Blood Pressure Missing	96 (1.94)	95 (1.93)	0	109 (2.22)	95 (1.93)	-0.02
**Albumin**						

© 2022 Keyhani S et al. *JAMA Network Open.*

	Propensity Weighted			Propensity Matched		
	**Non-use**	**Cannabis use**	**Diff weighted**	**Non-use**	**Cannabis use**	**Diff matched**
Albumin < 2.5	7 (0.14)	7 (0.14)	0	7 (0.14)	7 (0.14)	0
Albumin 2.5 to 3.5	194 (3.9)	189 (3.85)	0	184 (3.74)	189 (3.85)	0.005
Albumin > 3.5	1451 (29.19)	1433 (29.16)	0	1450 (29.51)	1433 (29.16)	-0.008
Albumin missing	3319 (66.78)	3285 (66.85)	0.001	3273 (66.61)	3285 (66.85)	0.005
Low hemoglobin	93 (1.87)	88 (1.79)	-0.001	91 (1.85)	88 (1.79)	-0.005
**Mental Health Conditions**						
Psychosis	152 (3.06)	149 (3.03)	0	130 (2.65)	149 (3.03)	0.023
Depression	1724 (34.69)	1706 (34.72)	0	1668 (33.94)	1706 (34.72)	0.016
Bipolar	175 (3.53)	173 (3.52)	0	178 (3.62)	173 (3.52)	-0.005
PTSD	1498 (30.13)	1493 (30.38)	0.003	1457 (29.65)	1493 (30.38)	0.016
Anxiety	820 (16.5)	810 (16.48)	0	820 (16.69)	810 (16.48)	-0.005
Self-harm	12 (0.24)	10 (0.2)	0	12 (0.24)	10 (0.2)	-0.009
**Health Behaviors**						
Opioid use disorder/dependence	244 (4.92)	227 (4.62)	-0.003	218 (4.44)	227 (4.62)	0.009
Benzodiazepine use disorder/dependence	27 (0.54)	22 (0.45)	-0.001	20 (0.41)	22 (0.45)	0.006
Amphetamine use disorder/dependence	65 (1.32)	51 (1.04)	-0.003	51 (1.04)	51 (1.04)	0
Other drug use disorder/dependence	193 (3.88)	169 (3.44)	-0.004	152 (3.09)	169 (3.44)	0.019
Alcohol use disorder	495 (9.97)	467 (9.5)	-0.005	446 (9.08)	467 (9.5)	0.015
Elevated audit C score	291 (5.85)	279 (5.68)	-0.002	281 (5.72)	279 (5.68)	-0.002
Current tobacco use	2299 (46.26)	2239 (45.56)	-0.007	2265 (46.09)	2239 (45.56)	-0.011
Positive amphetamine lab	136 (2.74)	130 (2.65)	-0.001	126 (2.56)	130 (2.65)	0.005
Positive benzodiazepine lab	761 (15.31)	745 (15.16)	-0.001	722 (14.69)	745 (15.16)	0.013
Positive cocaine lab	74 (1.48)	62 (1.26)	-0.002	49 (1)	62 (1.26)	0.025
**Pain Disorders**						
Back and spine disorders	3058 (61.52)	3032 (61.7)	0.002	3012 (61.29)	3032 (61.7)	0.008
Neck and spine disorders	1038 (20.89)	1044 (21.25)	0.004	1044 (21.25)	1044 (21.25)	0
Osteoarthritis	1650 (33.19)	1639 (33.35)	0.002	1634 (33.25)	1639 (33.35)	0.002
Neuropathy	848 (17.07)	845 (17.2)	0.001	869 (17.68)	845 (17.2)	-0.013
Headache	552 (11.1)	539 (10.97)	-0.001	545 (11.09)	539 (10.97)	-0.004
Trumatic brain injury	3 (0.07)	4 (0.08)	0	2 (0.04)	4 (0.08)	0.016
**Social Risk**						
Homelessness	181 (3.64)	164 (3.34)	-0.003	181 (3.68)	164 (3.34)	-0.019
Lack of social support	3 (0.06)	3 (0.06)	0	3 (0.06)	3 (0.06)	0

© 2022 Keyhani S et al. *JAMA Network Open.*

	Propensity Weighted			Propensity Matched		
	Non-use	Cannabis use	Diff weighted	Non-use	Cannabis use	Diff matched
**VA Priority group**						
VA Priority group 1	1111 (22.35)	1123 (22.85)	0.005	1105 (22.49)	1123 (22.85)	0.009
VA Priority group 2	258 (5.2)	255 (5.19)	0	264 (5.37)	255 (5.19)	-0.008
VA Priority group 3	434 (8.73)	427 (8.69)	0	457 (9.3)	427 (8.69)	-0.021
VA Priority group 4	83 (1.67)	83 (1.69)	0	80 (1.63)	83 (1.69)	0.005
VA Priority group 5	1607 (32.34)	1566 (31.87)	-0.005	1570 (31.95)	1566 (31.87)	-0.002
VA Priority group 6	157 (3.15)	156 (3.17)	0	163 (3.32)	156 (3.17)	-0.008
VA Priority group 7	62 (1.24)	64 (1.3)	0.001	56 (1.14)	64 (1.3)	0.015
VA Priority group 8	370 (7.45)	363 (7.39)	-0.001	356 (7.24)	363 (7.39)	0.005
VA Priority unknown	888 (17.87)	877 (17.85)	0	863 (17.56)	877 (17.85)	0.007
**Utilization in past year**						
ICU visits within six months						
ICU visits within six months (none)	4829 (97.15)	4779 (97.25)	0.001	4787 (97.42)	4779 (97.25)	-0.01
ICU visits within six months (one)	117 (2.35)	111 (2.26)	-0.001	106 (2.16)	111 (2.26)	0.007
ICU visits within six months (two or more)	25 (0.51)	24 (0.49)	0	21 (0.43)	24 (0.49)	0.009
ICU visits within one year						
ICU visits within one year (none)	4748 (95.52)	4697 (95.58)	0.001	4699 (95.62)	4697 (95.58)	-0.002
ICU visits within one year (one)	163 (3.29)	166 (3.38)	0.001	166 (3.38)	166 (3.38)	0
ICU visits within one year (two or more)	59 (1.19)	51 (1.04)	-0.002	49 (1)	51 (1.04)	0.004
**Hospitalized within one year**	614 (12.36)	607 (12.35)	0	596 (12.13)	607 (12.35)	0.007
ED visits within six months						
ED visits within six months (none)	3914 (78.75)	3887 (79.1)	0.003	3941 (80.2)	3887 (79.1)	-0.027
ED visits within six months (one)	660 (13.27)	636 (12.94)	-0.003	597 (12.15)	636 (12.94)	0.024
ED visits within six months (two or more)	396 (7.97)	391 (7.96)	0	376 (7.65)	391 (7.96)	0.011
ED visits within one year						
ED visits within one year (none)	3408 (68.57)	3381 (68.8)	0.002	3419 (69.58)	3381 (68.8)	-0.017
ED visits within one year (one)	798 (16.06)	778 (15.83)	-0.002	780 (15.87)	778 (15.83)	-0.001
ED visits within one year (two or more)	764 (15.37)	755 (15.36)	0	715 (14.55)	755 (15.36)	0.023
VA visit count	32.06 (22.23)	31.96 (22.54)	-0.004	31.84 (21.35)	31.96 (22.54)	0.006
**Psychoactive Drugs in past year**						
Morphine Equivalents over prior 90-day period	230.14 (428.51)	229.61 (418.2)	-0.001	226.28 (406.07)	229.61 (418.2)	0.008
Long-acting opioid	1246 (25.06)	1247 (25.38)	0.003	1215 (24.73)	1247 (25.38)	0.015

© 2022 Keyhani S et al. *JAMA Network Open.*

	Propensity Weighted			Propensity Matched		
	**Non-use**	**Cannabis use**	**Diff weighted**	**Non-use**	**Cannabis use**	**Diff matched**
Alcohol use disorder treatment medication	7 (0.14)	9 (0.18)	0	9 (0.18)	9 (0.18)	0
Benzodiazepines	1175 (23.64)	1161 (23.63)	0	1150 (23.4)	1161 (23.63)	0.005
Gaba drugs	1125 (22.63)	1122 (22.83)	0.002	1097 (22.32)	1122 (22.83)	0.012
Muscle relaxants	761 (15.32)	759 (15.45)	0.001	745 (15.16)	759 (15.45)	0.008
Antidepressants	1579 (31.77)	1575 (32.05)	0.003	1585 (32.25)	1575 (32.05)	-0.004
Antipsychotics	357 (7.18)	361 (7.35)	0.002	340 (6.92)	361 (7.35)	0.017
Sedatives	10 (0.2)	10 (0.2)	0	14 (0.28)	10 (0.2)	-0.016
**Number of psychoactive drugs in past year**						
1	1882 (37.87)	1853 (37.71)	-0.002	1865 (37.95)	1853 (37.71)	-0.005
2	1702 (34.24)	1672 (34.03)	-0.002	1671 (34)	1672 (34.03)	0
3	952 (19.14)	953 (19.39)	0.002	961 (19.56)	953 (19.39)	-0.004
4	345 (6.94)	343 (6.98)	0	333 (6.78)	343 (6.98)	0.008
5	90 (1.81)	93 (1.89)	0.001	84 (1.71)	93 (1.89)	0.014
**Care Assessment Need (CAN) score**						
CAN score < 10	0 (0)	0 (0)	0			
CAN score 10 - 19.9	12 (0.24)	12 (0.24)	0	12 (0.24)	12 (0.24)	0
CAN score 20 - 29.9	104 (2.1)	102 (2.08)	0	101 (2.06)	102 (2.08)	0.001
CAN score 30 - 39.9	365 (7.34)	362 (7.37)	0	366 (7.45)	362 (7.37)	-0.003
CAN score 40 - 49.9	591 (11.88)	590 (12.01)	0.001	583 (11.86)	590 (12.01)	0.004
CAN score 50 - 59.9	728 (14.65)	729 (14.84)	0.002	697 (14.18)	729 (14.84)	0.018
CAN score 60 - 69.9	818 (16.46)	812 (16.52)	0.001	840 (17.09)	812 (16.52)	-0.015
CAN score 70 - 79.9	914 (18.38)	899 (18.29)	-0.001	922 (18.76)	899 (18.29)	-0.012
CAN score 80 - 89.9	833 (16.76)	818 (16.65)	-0.001	785 (15.97)	818 (16.65)	0.018
CAN score 90 - 98.9	536 (10.78)	518 (10.54)	-0.002	537 (10.93)	518 (10.54)	-0.012
CAN score missing	70 (1.4)	72 (1.47)	0.001	71 (1.44)	72 (1.47)	0.002
**Year Index Urine Drug Screen was ordered**						
2014	2544 (51.17)	2533 (51.55)	0.004	2521 (51.3)	2533 (51.55)	0.005
2015	1384 (27.84)	1385 (28.18)	0.003	1402 (28.53)	1385 (28.18)	-0.008
2016	591 (11.9)	563 (11.46)	-0.004	563 (11.46)	563 (11.46)	0
2017	255 (5.12)	242 (4.92)	-0.002	242 (4.92)	242 (4.92)	0
2018	138 (2.78)	137 (2.79)	0	131 (2.67)	137 (2.79)	0.007
2019	59 (1.19)	54 (1.1)	-0.001	55 (1.12)	54 (1.1)	-0.002

© 2022 Keyhani S et al. *JAMA Network Open.*

* Facility level variable not shown

© 2022 Keyhani S et al. *JAMA Network Open.*

eFigure 3. Survival Probability Among Adults Ages 65 Years and Older Followed up for 180 Days Receiving Prescription Opioid Therapy in Prior 90 Days By Cannabis Use

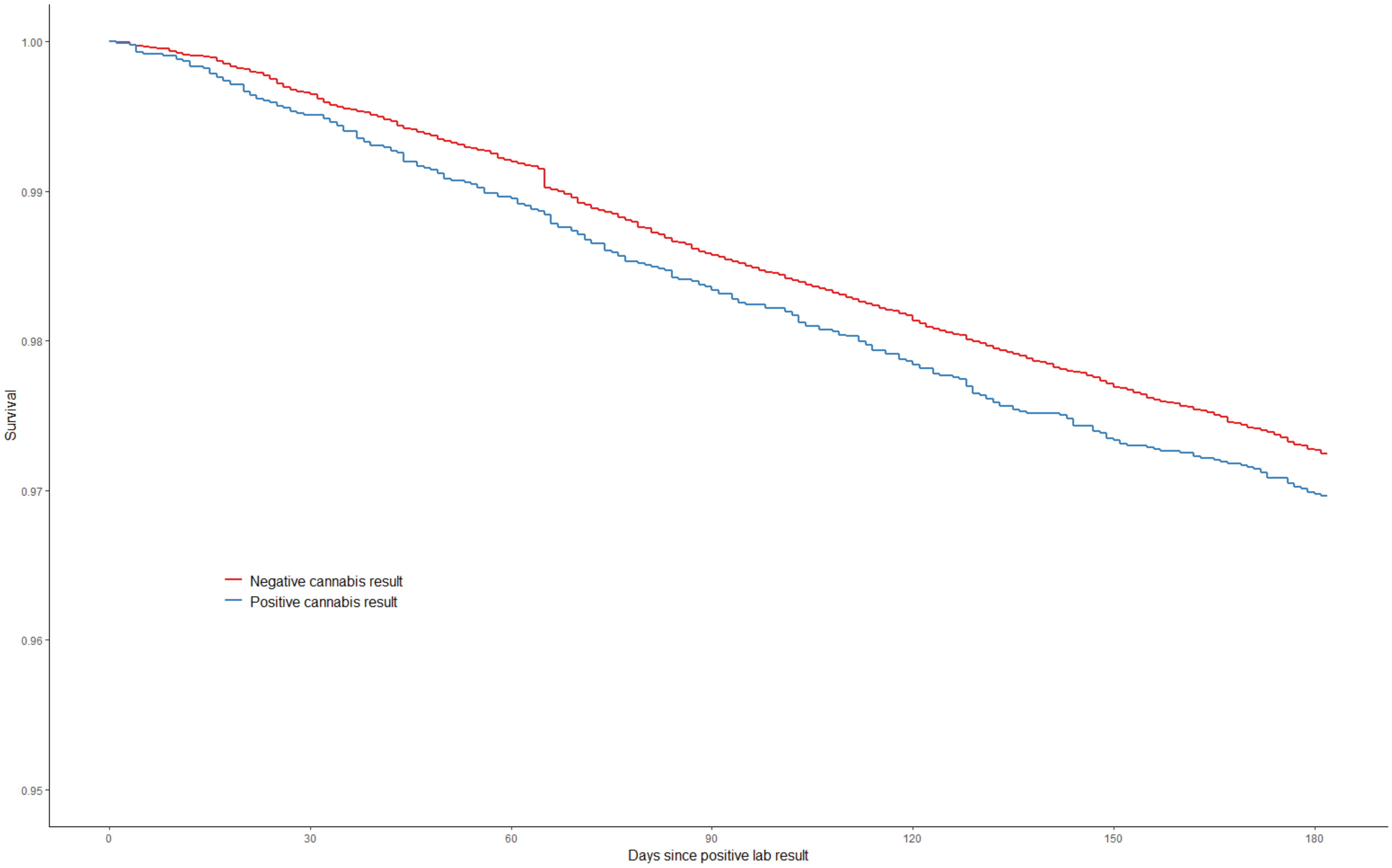

© 2022 Keyhani S et al. *JAMA Network Open.*

eFigure 4. Probability of Emergency Department Visits, Hospitalization, or Mortality Among Adults Aged 65 Years and Older Followed up for 180 Days Receiving Any Prescription Opioid Therapy By Cannabis Use

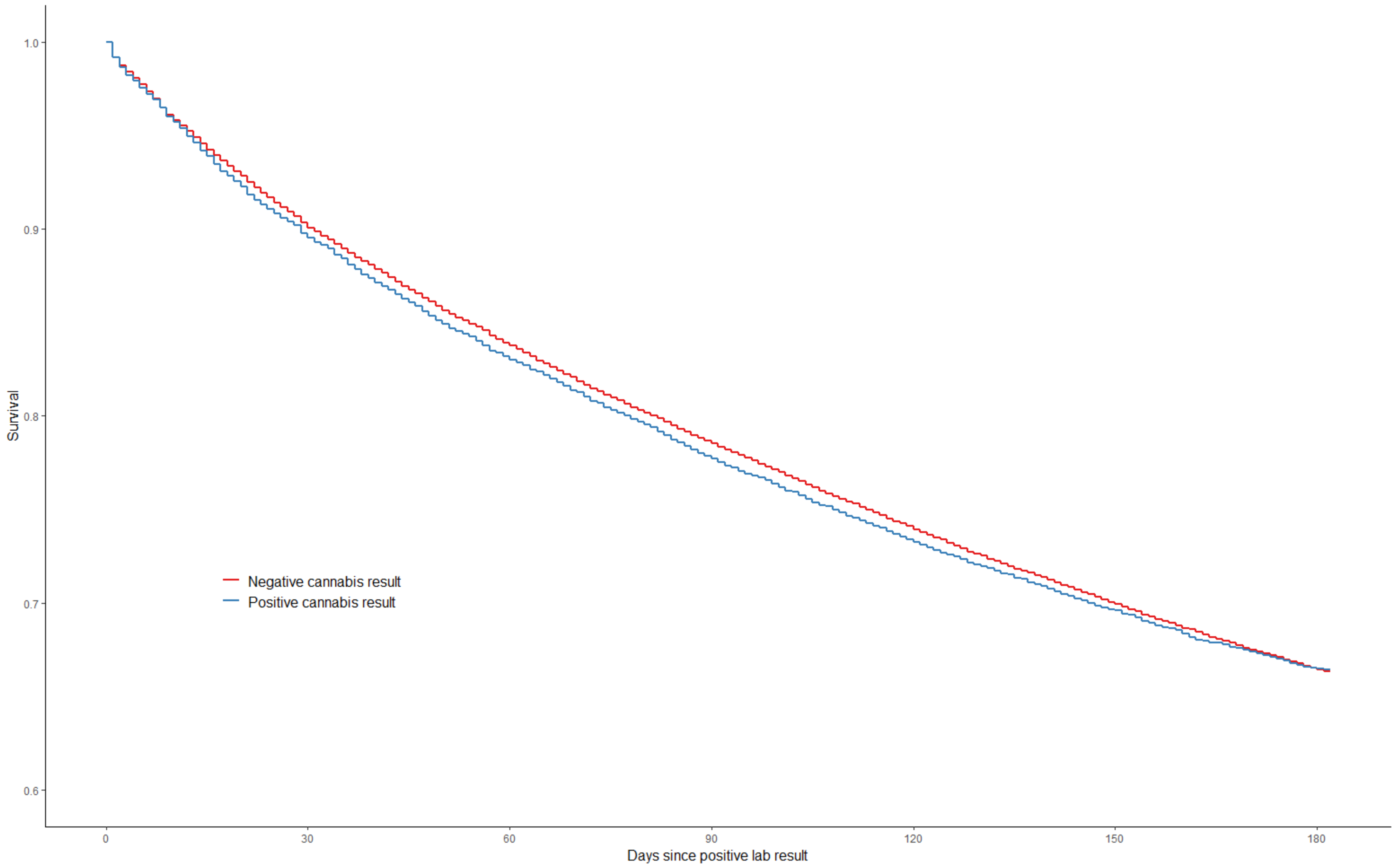

© 2022 Keyhani S et al. *JAMA Network Open.*

eTable 10. Association of Cannabis Use With 90- and 180-Day Adverse Events Among Adults Ages 65 Years and Older Receiving Prescription Opioids (Propensity-Weighting and Matching Approach)

	Propensity Weighted Sample				Propensity Matched Sample			
	**Unadjusted events by arm**	**90-day mortality**	**Unadjusted events by arm**	**180-day mortality**	**Unadjusted events by arm**	**90-day mortality**	**Unadjusted events by arm**	**180-day mortality**
	N (%)	HR, 95%CI	N (%)	HR, 95%CI	N (%)	HR, 95%CI	N (%)	HR, 95%CI
All adults 65 and older on opioids in the past 90 days	Total = 1835 Opioids only = 1694 (1.33%) Opioid and cannabis = 141 (1.68%)	Hazard ratio = 1.17 (0.91, 1.49) p = 0.20	Total = 3805 Opioids only = 3551 (2.8%) Opioid and cannabis = 254 (3.04%)	Hazard ratio = 1.10 (0.93, 1.29) p = 0.23	Total = 241 Opioids only = 100 (1.19%) Opioid and cannabis = 141 (1.68%)	Hazard ratio = 1.41 (1.09, 1.82) p = 0.008	Total = 471 Opioids only = 217 (2.59%) Opioid and cannabis = 254 (3.04%)	Hazard ratio = 1.18 (0.98, 1.40) p = 0.08
All adults 65 and older on LTOT (opioids on >84 days out of the past 90 days)	Total = 856 Opioids only = 787 (1.08%) Opioid and cannabis = 69 (1.4%)	Hazard ratio = 1.55 (1.17, 2.04) p = 0.002	Total = 1921 Opioids only = 1799 (2.47%) Opioid and cannabis = 122 (2.48%)	Hazard ratio = 1.17 (0.95, 1.44) p = 0.13	Total = 113 Opioids only = 44 (0.9%) Opioid and cannabis = 69 (1.4%)	Hazard ratio = 1.57 (1.07, 2.29) p = 0.02	Total = 226 Opioids only = 104 (2.12%) Opioid and cannabis = 122 (2.48%)	Hazard ratio = 1.18 (0.90, 1.5) p = 0.22
	**Propensity Weighted Sample**				**Propensity Matched Sample**			
	**Unadjusted events by arm**	**90-day ED visits, hospitalization or mortality**	**Unadjusted events by arm**	**180-day ED visits, hospitalization or mortality**	**Unadjusted events by arm**	**90-day ED visits, hospitalization or mortality**	**Unadjusted events by arm**	**180-day ED visits, hospitalization or mortality**
	N (%)	HR, 95%CI	N (%)	HR, 95%CI	N (%)	HR, 95%CI	N (%)	HR, 95%CI
All adults 65 or older on opioids in the last 90 days	Total = 29965 Opioids only = 28175 (22.88%) Opioid and cannabis = 1790 (22.5%)	Hazard ratio = 1.03 (0.98, 1.09) p = 0.21	Total = 46912 Opioids only = 44240 (35.92%) Opioid and cannabis = 2672 (33.58%)	Hazard ratio = 1.00 (0.95, 1.04) p = 0.97	Total = 3481 Opioids only = 1691 (21.2%) Opioid and cannabis = 1790 (22.5%)	Hazard ratio = 1.07 (1, 1.14) p = 0.05	Total = 5339 Opioids only = 2667 (33.43%) Opioid and cannabis = 2672 (33.58%)	Hazard ratio = 1.01 (0.95, 1.06) p = 0.65
All adults 65 and older on LTOT (opioids on >84 days out of the past 90 days)	Total = 16784 Opioids only = 15778 (21.96%) Opioid and cannabis = 1006 (20.97%)	Hazard ratio = 1.05 (0.98, 1.13) p = 0.15	Total = 26795 Opioids only = 25236 (35.13%) Opioid and cannabis = 1559 (32.5%)	Hazard ratio = 1.03 (0.97, 1.08) p = 0.36	Total = 1935 Opioids only = 929 (19.35%) Opioid and cannabis = 1006 (20.97%)	Hazard ratio = 1.09 (1.01, 1.19) p = 0.05	Total = 3024 Opioids only = 1465 (30.51%) Opioid and cannabis = 1559 (32.5%)	Hazard ratio = 1.08 (1.01, 1.16) p = 0.03

eTable 11. Abstraction Tool for Chart Review

© 2022 Keyhani S et al. *JAMA Network Open.*

Search for evidence of behaviors/characteristics in the following notes:	Labs, Primary Care, Psychiatric/Mental Health, Pharmacy (exclude Clinical Pharmacy notes), Pain clinic-related, Social Work, Emergency Department, Urgent Care
Is there any mention of cannabis use in notes within the abstraction period?	1, Yes<br>2, No<br>100, Don’t know<br>Note: Use search terms: marij, mj, THC, cannabis
Is there any evidence of a cannabis use disorder diagnosis, abuse, addiction, or dependence within the abstraction period?	1, Yes<br>2, No<br>100, Don’t know
Is there any evidence that the provider took action regarding polydrug use? (e.g. recommended reducing or ceasing use of one drug, referral to substance abuse treatment)	1, Yes<br>2, No<br>100, Don’t know
Is there any evidence of other substance use within the abstraction period?	1, Active illicit drug use in the past year (other than cannabis)<br>2, Active drug use disorder diagnosis, abuse, addiction, or dependence documented by providers in the past year (other than cannabis)<br>3, Active alcohol use disorder or dependence documented by the providers in the past year<br>4, DUI, trauma, crash, or arrest related to intoxication or substance use<br>5, Using a substance not prescribed by a provider to control pain (besides cannabis, e.g. alcohol, other illicit substances, benzos)<br>6, Using cannabis to control pain<br>7, None of the above<br>100, Don't know
Is there any evidence of current tobacco use within the abstraction period?	Note: Use search terms: tob, tobacco, cig<br><br>1, Yes<br>2, No<br>100, Don’t know
Is there any evidence of alcohol use within the abstraction period?	Note: Use search terms: alcohol, etoh, audit, drink<br><br>1, Yes<br>2, No<br>100, Don’t know

© 2022 Keyhani S et al. *JAMA Network Open.*

Is there any evidence of multiple missed PCP appointments (more than two) within the abstraction period?	1, Yes<br>2, No<br>100, Don't know
Is there any evidence of the following patient behaviors related to opioids within the abstraction period?	1, Resisted therapy changes/alternative therapy (refuses direct treatment for pain)<br>2, Resisted referrals to other pain specialists<br>3, Refused the request to taper<br>4, Unauthorized/unsanctioned dose escalation<br>5, Obtaining opioids from non-VA source providers<br>6, Obtaining opioids from multiple non-VA source providers (multiple hospitals, providers)<br>7, Solicited opioids from multiple providers in VA<br>8, Obtaining opioids from street/another non-medical source<br>9, Multiple phone calls/visits requesting opioids (three or more requests within 30 days)<br>10, Requests for specific opioids or dosages (other than as prescribed)<br>11, Running out of opioids and/or requesting early refills (excluding travel)<br>12, Reported lost, ruined, or stolen prescriptions<br>13, Injury or intoxication from opioids (any visit related to opioids, patient/family reports)<br>14, Emergency room visits to get opioids<br>15, Saved/hoarded unused medication<br>16, Selling prescription drugs (diversion)<br>17, Patient threat related to opioids (threat of violence, lawsuit, disruption, disengagement from care)<br>18, Patient seeking new provider due to disagreement over opioid prescription<br>19, Patient/family reports problems with opioid addiction<br>20, Legal issues related to opioids<br>21, Requesting non-VA opioid script<br>22, Other: _____<br>23, None of the above<br>100, Don't know
Is there any evidence of intentional self-inflicted harm or threats of self-inflicted harm related to opioids?	1, Yes<br>2, No<br>100, Don't know
Is there any evidence of the following provider/clinic actions within the abstraction period?	1, Provider documented plans to terminate or reduce opioids (dose/days supply; refer to opioid conversion table)<br>2, Provider documented an opioid use disorder<br>3, Provider documented an opioid overdose, or use of Naloxone

© 2022 Keyhani S et al. *JAMA Network Open.*

	4, Patient has been told to leave a clinic, hospital, or ER, or been banned from one of these places due to behavior related to an opioid<br>5, Patient was discharged (released) from practice for aberrant behavior or placed on a watch list<br>6, Patient was referred to the Disruptive Behavior Committee (DBC)<br>7, Patient was referred to the Prescription Opioid Safety Team (POST)<br>8, Patient has a Stratification Tool for Opioid Risk Mitigation (STORM) note from pharmacy indicating moderate or high risk of harm from opioid use?<br>9, Other: ____<br>10, None of the above<br>100, Don't know

© 2022 Keyhani S et al. *JAMA Network Open.*

eTable 12. High-Risk Behaviors, Adverse Outcomes, and Clinician Actions Associated With Opioid Use Among Adults Aged 65 Years and Older

	Opioid Use (N=285)	Opioid & Cannabis Use (N=142)	Total (N=427)	P-Value
**Evidence of Current Tobacco Use**	**90 (31.57%)**	**55 (38.7%)**	**148 (34.66%)**	**0.17**
**Substance use related findings and behaviors**	**19 (6.67%)**	**23 (16.2%)**	**42 (9.8%)**	0.003
Cannabis use disorder diagnosis, abuse, addiction, or dependence	0 (0.0%)	15 (10.6%)	15 (3.5%)	
UDT positive for another substance other than cannabis	8 (2.8%)	5 (3.5%)	13 (3.0%)	
Active drug use disorder diagnosis, abuse, addiction, or dependence	4 (1.4%)	3 (2.1%)	7 (1.6%)	
Using substance not prescribed by provider to control pain	7 (2.5%)	32 (22.5%)	39 (9.1%)	
Active illicit drug use in past year (other than cannabis)	1 (0.35%)	3 (2.1%)	4 (.94%)	
DUI, trauma, crash, or arrest related to intoxication or substance use	0 (0.0%)	0 (0.0%)	0 (0.0%)	
**Alcohol use related behaviors**	**131 (45.96%)**	**79 (55.6%)**	**210 (49.18%)**	0.07
Alcohol use within abstraction period	131 (46.0%)	79 (55.6%)	210 (49.2%)	
Active alcohol use disorder or dependence	6 (2.1%)	2 (1.41%)	8 (1.87%)	
**Treatment related behaviors**	**88 (30.87%)**	**71 (50.0%)**	**159 (37.2%)**	<0.001
Requesting for specific opioids or dosages	27 (0.95%)	28 (19.7%)	55 (12.9%)	
Running out of opioids, requesting early refills	33 (11.6%)	24 (16.9%)	57 (13.3%)	
Unauthorized/unsanctioned dose escalation	26 (9.12%)	23 (16.2%)	49 (11.5%)	
Emergency room visits to get opioids	12 (4.21%)	8 (5.63%)	20 (4.68%)	
Resisted therapy changes/alternative therapy	13 (4.56%)	15 (10.6%)	28 (6.56%)	
Obtaining opioids from multiple non-VA source providers	2 (0.70%)	3 (2.11%)	5 (1.17%)	
Obtaining opioids from non-VA source providers	13 (4.56%)	12 (8.45%)	25 (5.85%)	
Multiple phone calls/visits requesting opioids	10 (3.51%)	7 (4.93%)	17 (3.98%)	
Reported lost, ruined, or stolen prescriptions	9 (3.16%)	2 (1.41%)	11 (2.58%)	
Patient seeking new provider due to disagreement over opioid prescription	0 (0.0%)	1 (0.70%)	1 (0.23%)	
Refused the request to taper	2 (0.70%)	9 (6.34%)	11 (2.58%)	
Saved/hoarded unused medication	11 (3.86%)	4 (2.82%)	15 (3.5%)	
Obtaining opioids from street/another non-medical source	2 (0.70%)	3 (2.11%)	5 (1.17%)	
Resisting referrals to other pain specialists	5 (1.75%)	4 (2.82%)	9 (2.11%)	
Solicited opioids from multiple providers in VA	0 (0.0%)	1 (0.70%)	1 (0.23%)	
Diagnosed with Opioid Use disorder	2 (0.70%)	3 (2.11%)	5 (1.17%)	
Patient has a Stratification Tool for Opioid Risk Mitigation (STORM) note from pharmacy indicating moderate or high risk of harm from opioid use	2 (0.70%)	0 (0.0%)	2 (0.47%)	

© 2022 Keyhani S et al. *JAMA Network Open.*

**Adverse Outcomes Related to Opioid Use**	**6 (2.1%)**	**7 (4.9%)**	**13 (3.0%)**	0.19
Evidence of self-inflicted harm or threats or self-inflicted harm related to opioids	3 (1.05%)	3 (2.11%)	6 (1.41%)	
Opioid overdose or use of Naloxone	2 (0.70%)	2 (1.41%)	4 (.94%)	
Injury or intoxication from opioids	3 (1.05%)	2 (1.41%)	5 (1.17%)	
Legal issues related to opioids	0 (0.0%)	1 (0.70%)	1 (0.23%)	
**Provider Actions Related to Opioid Use**	**28 (9.8%)**	**72 (50.7%)**	**100 (23.4%)**	<0.001
Provider took action regarding polydrug use	0 (0.0%)	60 (42.3%)	60 (14.1%)	
Provider documented plans to terminate or reduce opioids	26 (9.12%)	46 (32.4%)	72 (16.9%)	
Patient was discharged from practice for aberrant behavior or placed on a watch list	1 (0.35%)	3 (2.11%)	4 (.94%)	
Patient was referred to the Disruptive Behavior Committee (DBC)	1 (0.35%)	2 (1.41%)	3 (0.70%)	
Patient has been told to leave a clinic, hospital, or ER, or been banned from one of these places due to behavior related to an opioid	0 (0.0%)	0 (0.0%)	0 (0.0%)	
**Other High-Risk behaviors**	**9 (3.16%)**	**9 (6.3%)**	**18 (4.2%)**	0.2
Patient threat related to opioids	2 (0.70%)	5 (3.52%)	7 (1.6%)	
Multiple missed primary care appointments (more than 2)	3 (1.05%)	1 (0.70%)	4 (.94%)	
Patient/family reports problems with opioids addiction	5 (1.75%)	4 (2.82%)	9 (2.11%)	
Requesting non-VA opioids script	0 (0.0%)	1 (0.70%)	1 (0.23%)	
Selling or giving away prescription drugs	0 (0.0%)	0 (0.0%)	0 (0.0%)	

© 2022 Keyhani S et al. *JAMA Network Open.*
