# Supplementary material for: Association of a Positive Drug Screening for Cannabis With Mortality and Hospital Visits Among Veterans Affairs Enrollees Prescribed Opioids
Source: JAMA Netw Open. 2022 Dec 16;5(12):e2247201. doi: 10.1001/jamanetworkopen.2022.47201 (PMC9856228; doi:10.1001/jamanetworkopen.2022.47201)
Supplement: Supplement 2. — Data Sharing Statement [file jamanetwopen-e2247201-s002.pdf]

Data Sharing Statement

Keyhani S, Leonard S, Byers A, et al. Association of a positive drug screening for cannabis with mortality and hospital visits among veterans affairs enrollees prescribed opioids. *JAMA Netw Open*. 2022;5(12):e2247201. doi:10.1001/jamanetworkopen.2022.47201

Data

Data available: Yes
Data types: Other (please specify)
Additional Information: The dataset can be made available in accordance with VA policy and procedures
How to access data: The dataset is available on the VA Informatics Computing Infrastructure. Investigators interested in the data have to comply with VA policies and procedures to gain access.
When available: With publication

Supporting Documents

Document types: None

Additional Information

Who can access the data: researchers whose proposed use of the data has been approved
Types of analyses: For analyses that are approved by an VA approved IRB
Mechanisms of data availability: with a VA approved data access agreement
Any additional restrictions: The data is on VINCI. Interested investigators need to access VINCI

© 2022 Keyhani S et al. *JAMA Network Open*.
